# Correction: Isoform-specific disruption of the *TP73* gene reveals a critical role for TAp73γ in tumorigenesis via leptin

**DOI:** 10.7554/eLife.107690

**Published:** 2025-05-19

**Authors:** Xiangmudong Kong, Wensheng Yan, Wenqiang Sun, Yanhong Zhang, Hee Jung Yang, Mingyi Chen, Hongwu Chen, Ralph W de Vere White, Jin Zhang, Xinbin Chen

**Keywords:** Mouse

Kong X, Yan W, Sun W, Zhang Y, Yang HJ, Chen M, Chen H, de Vere White RW, Zhang J, Chen X. 2023. Isoform-specific disruption of the TP73 gene reveals a critical role for TAp73γ in tumorigenesis via leptin. *eLife* **12**:e82115. doi: 10.7554/eLife.82115.

Published 31 August 2023

Several images were identified to be duplicated by PubPeer and eLife: (1) In Figure 3F, the image of ΔNP73-KO panel (0h) duplicated the image of E11-KO#25 panel (0h); the image of ΔN73/E11-DKO#12 panel (0h) duplicated the image of ΔN73/E11-DKO#13 panel (0h); (2) In Figure 6J, the GAPDH bands duplicated the GAPDH bands in Figure 1G from a Cancers paper (https://doi.org/10.3390/cancers16010229); (3) In Figure 7D, the actin bands duplicated the actin bands in Figure 7—figure supplement 1A.

We apologize for the mistakes. Prior to submitting the manuscript to eLife, we have identified the duplicated images ourselves and corrected them. The correct figures were used for the initial submission. Per eLife request, the initial submission was deposited to BioRxiv on August 7, 2022 (https://www.biorxiv.org/content/10.1101/2022.08.07.503085v1.full), which is still available on BioRxiv portal. However, during the revision process, an incorrect version of the files was used for editing, which resulted in these errors. All the authors agree to the wording of the correction notice. The article has been corrected accordingly.

The corrected Figure 3 is shown here, with updated panel F:

**Figure 1:**
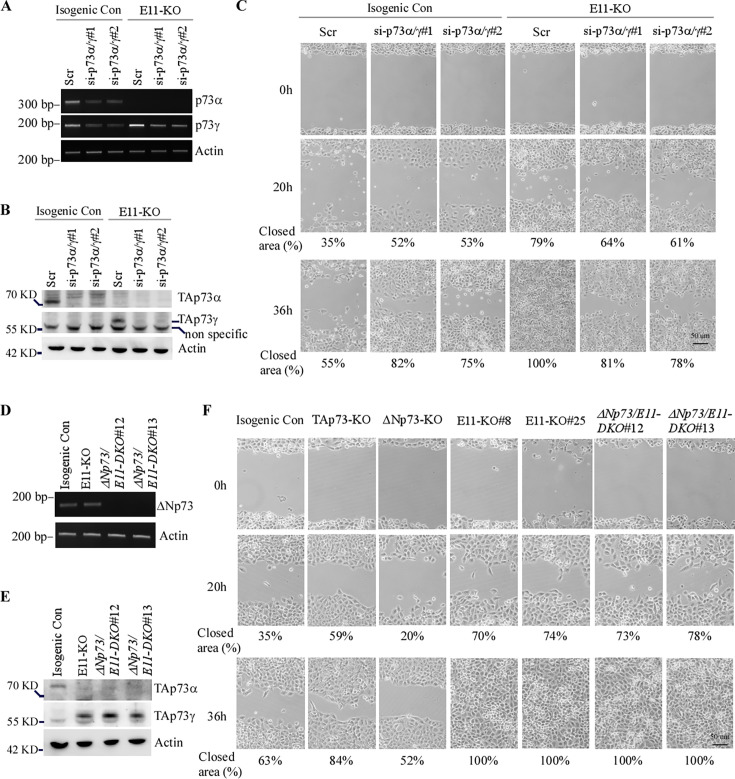


The originally published Figure 3 is shown for reference:

**Figure 2:**
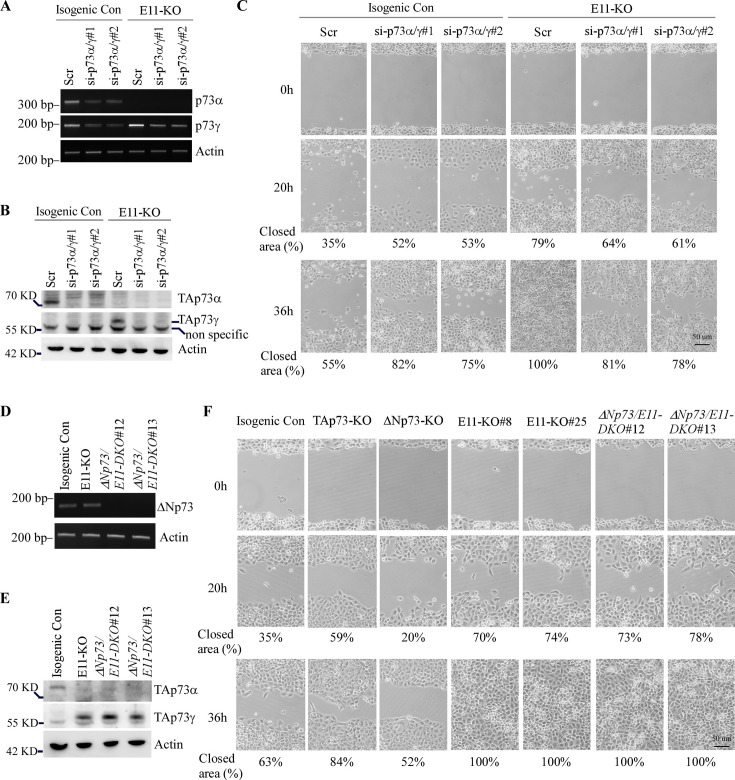


The corrected Figure 6 is shown here, with updated panel J:

**Figure 3:**
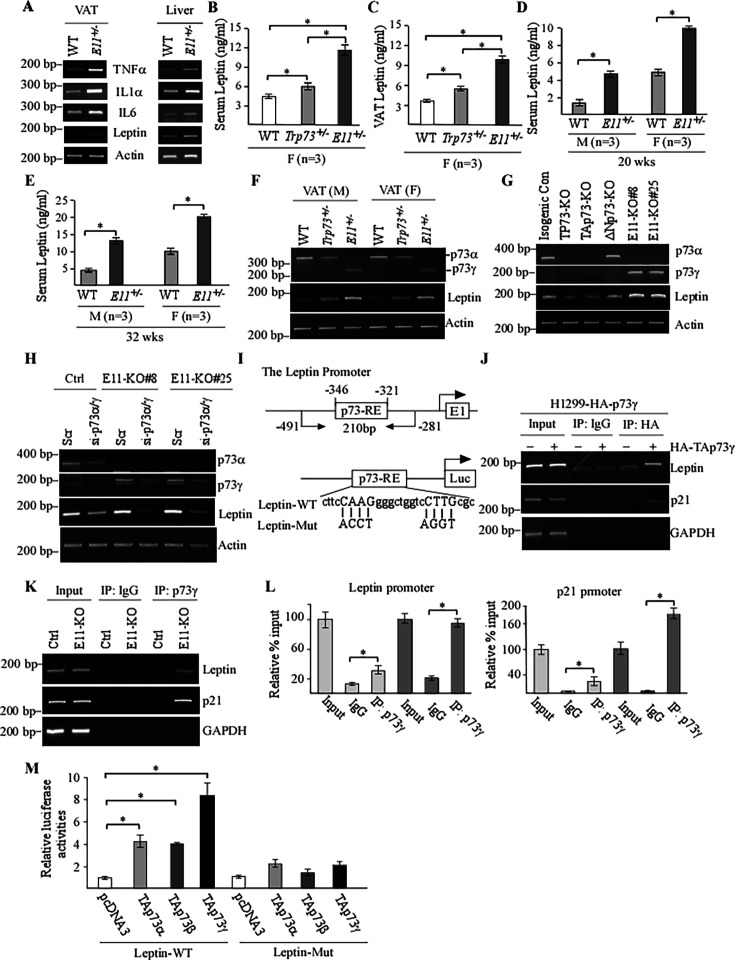


The originally published Figure 6 is shown for reference:

**Figure 4:**
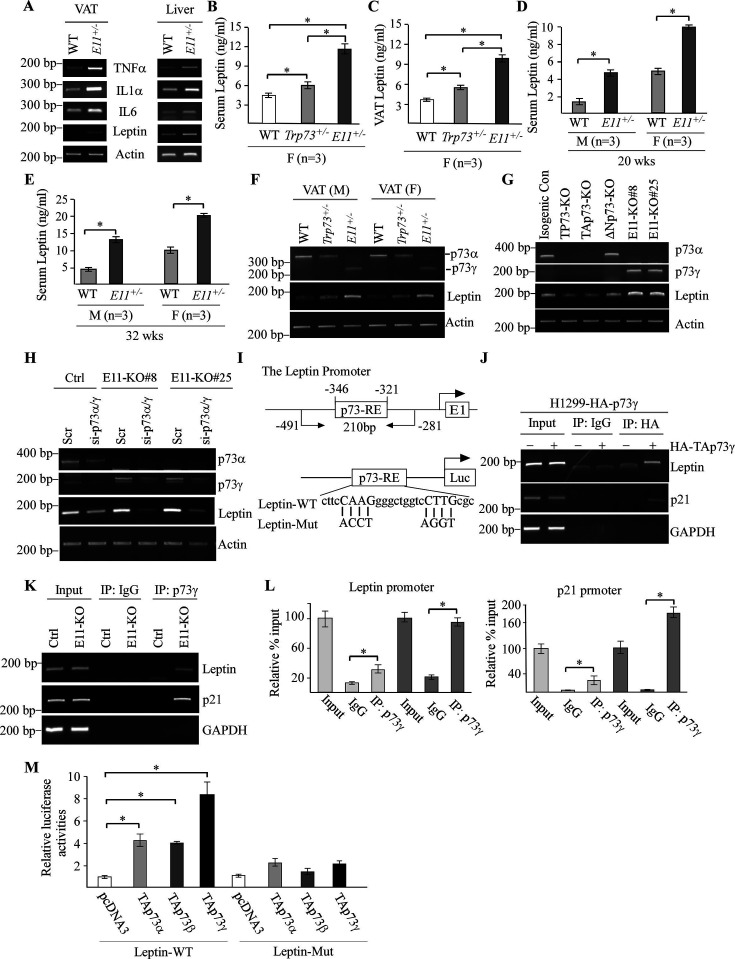


The corrected Figure 7—figure supplement 1 is shown here, with updated panel A:

**Figure 5:**
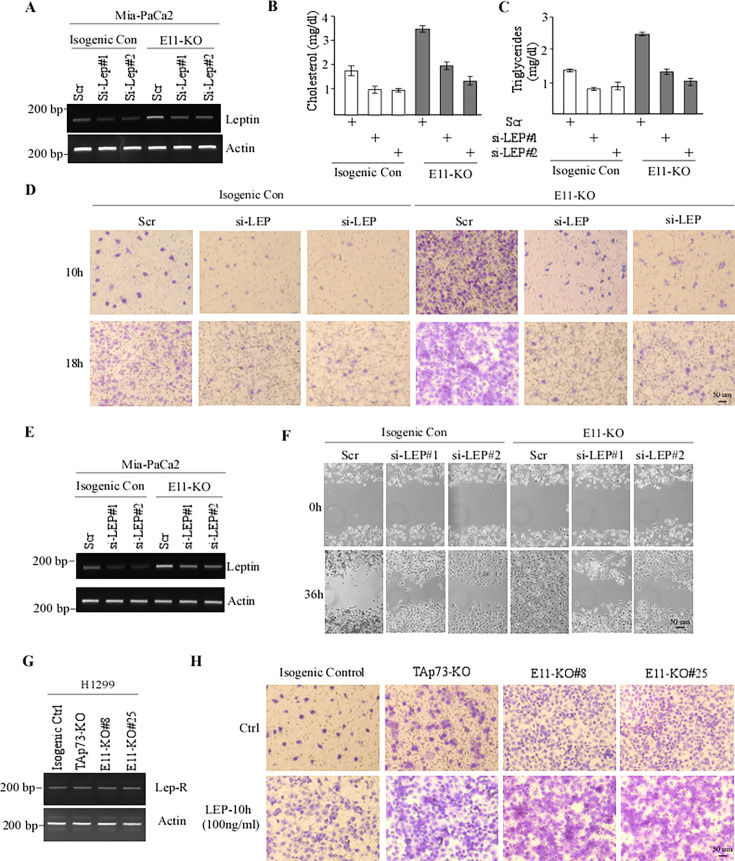


The originally published Figure 7—figure supplement 1 is shown for reference:

**Figure 6:**
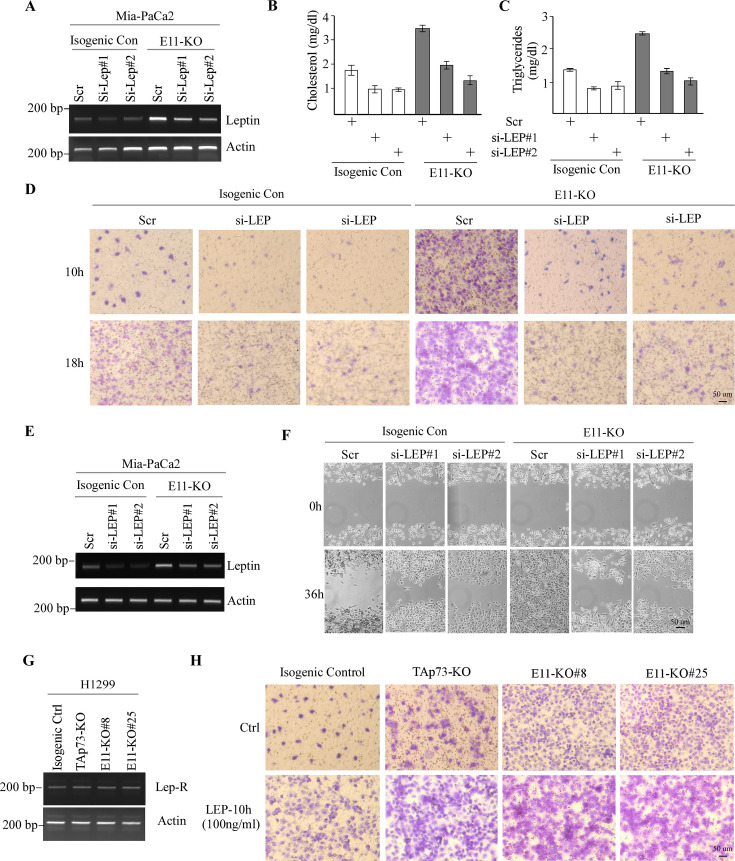


Whilst the published data in Figure 7—figure supplement 1—source data 1 are correct, we corrected this file to address a minor labelling error where the labels for panels 7A and 7E were switched. The article has been corrected accordingly.

