# Supplementary figures and images for: Isoform-specific disruption of the TP73 gene reveals a critical role for TAp73γ in tumorigenesis via leptin
Source: eLife. 2023 Aug 31;12:e82115. doi: 10.7554/eLife.82115 (PMC10471163; doi:10.7554/eLife.82115)

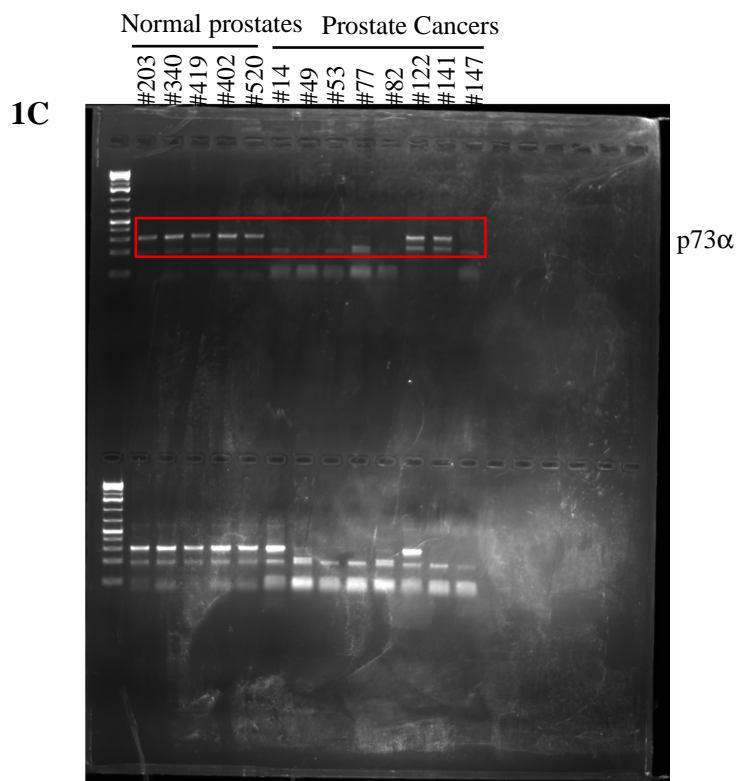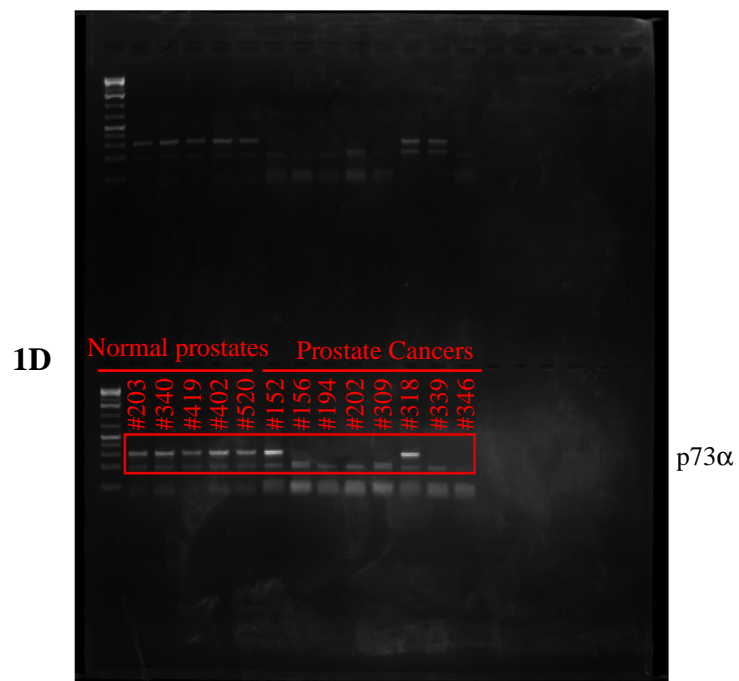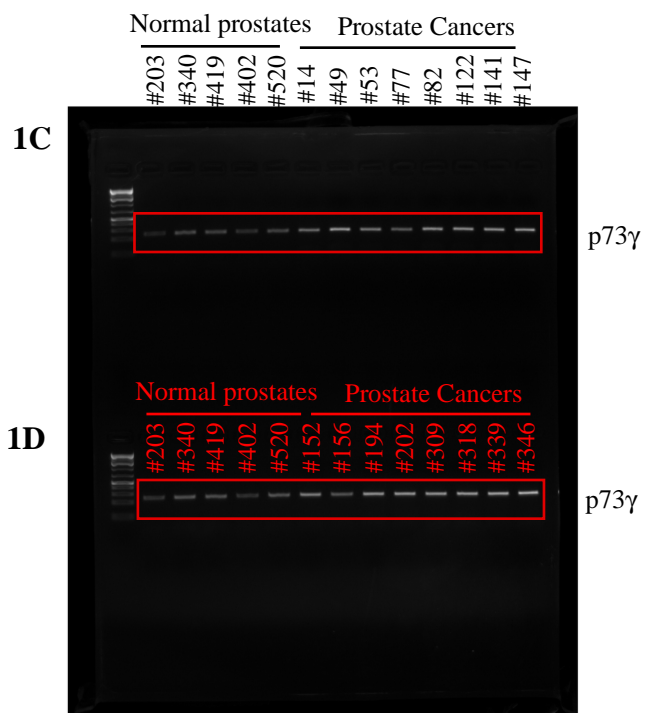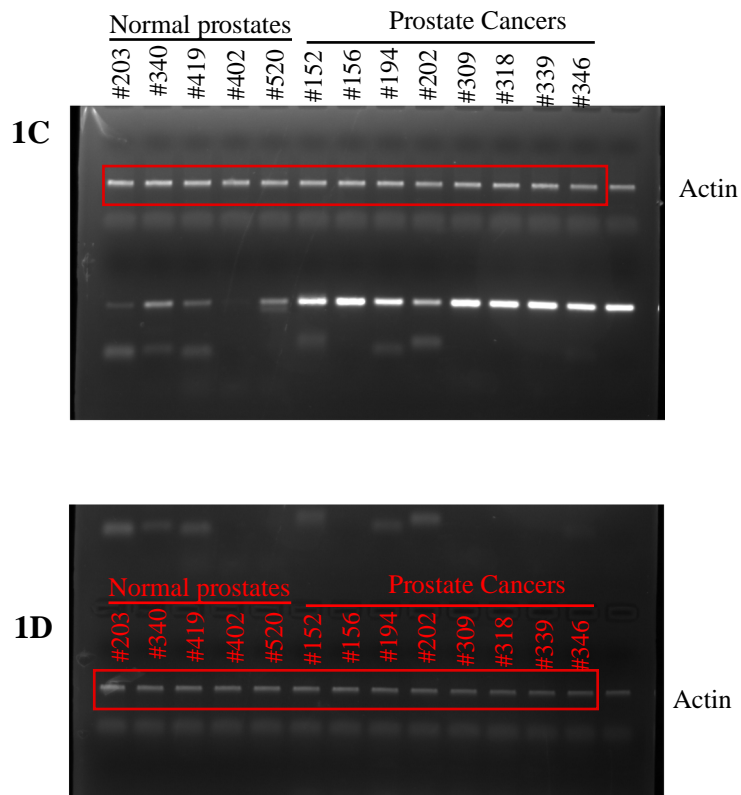

1E

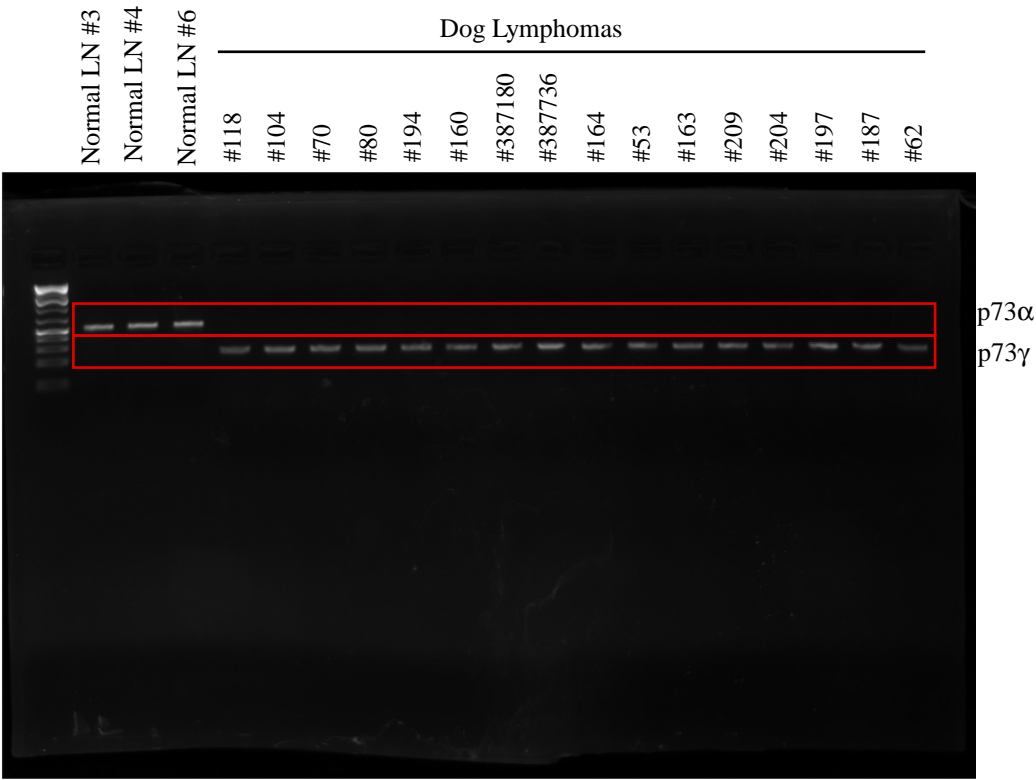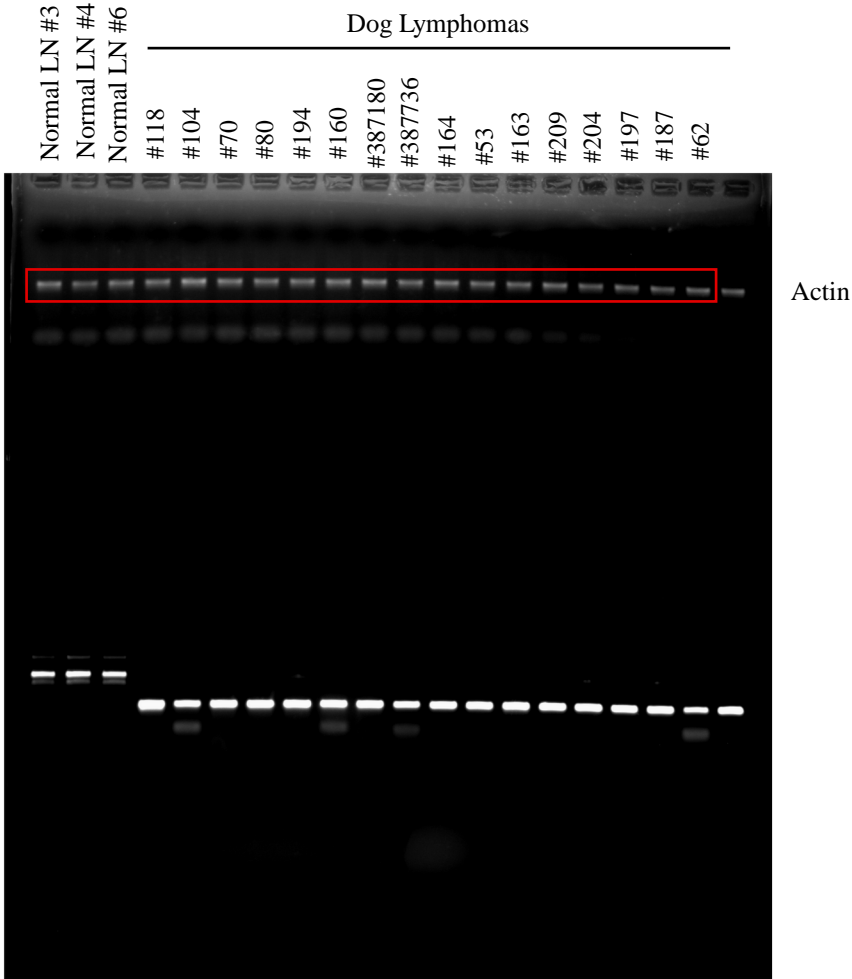

Supplement: Figure 1—source data 1. [file elife-82115-fig1-data1.pdf]

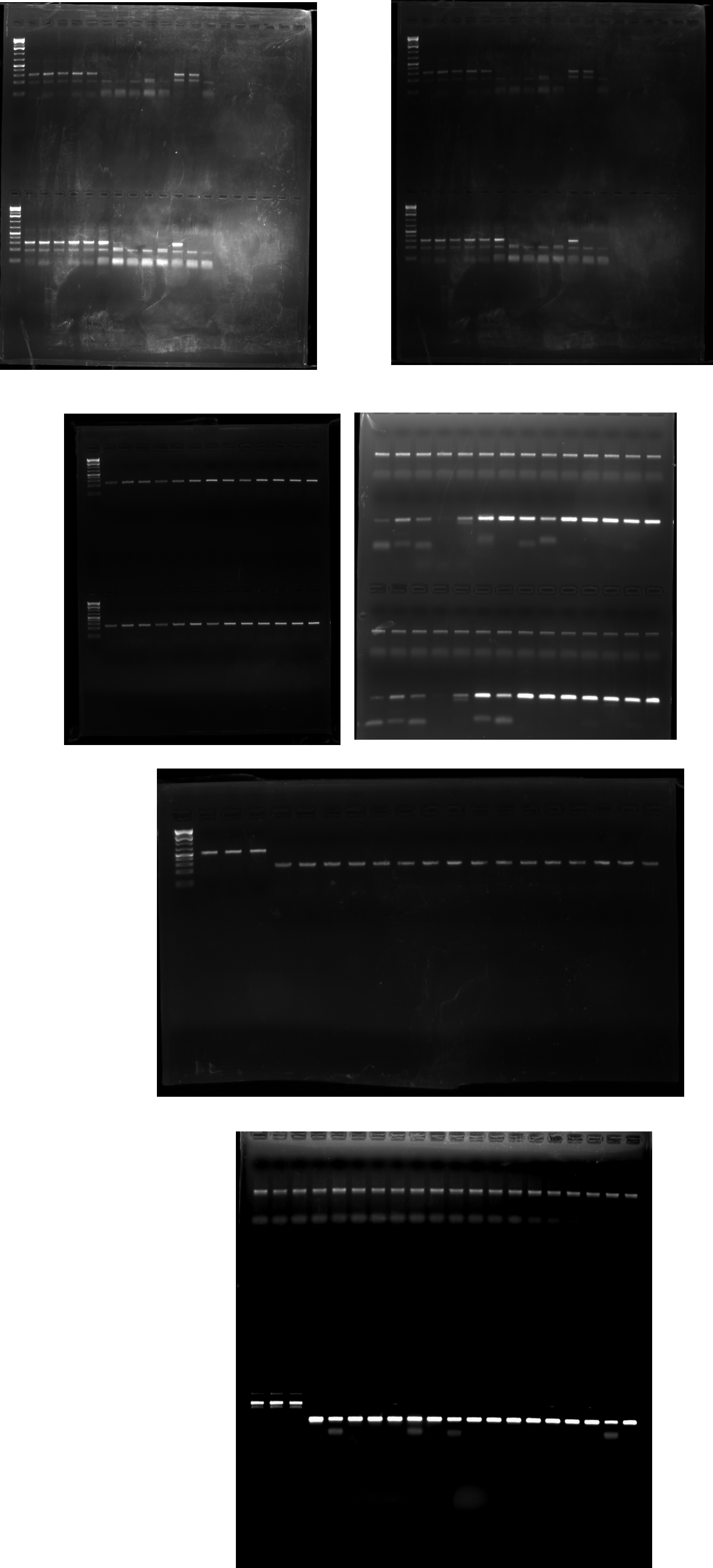

Supplement: Figure 1—source data 2. [file elife-82115-fig1-data2.tif]

2B

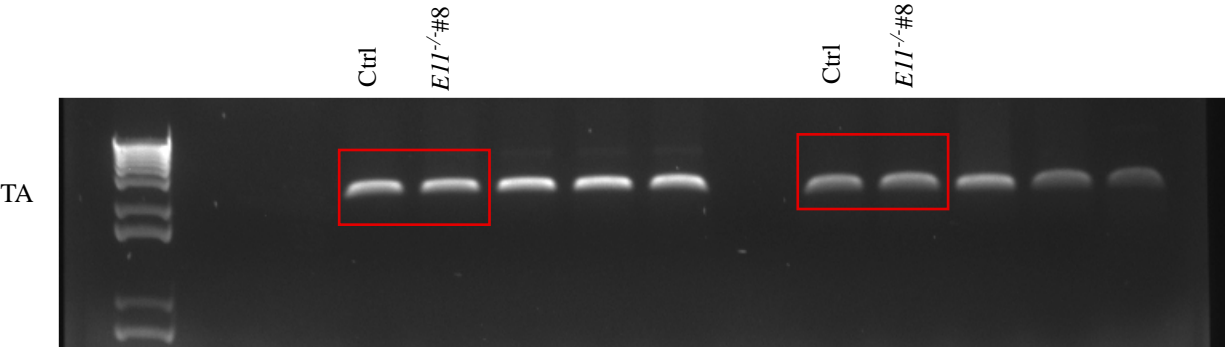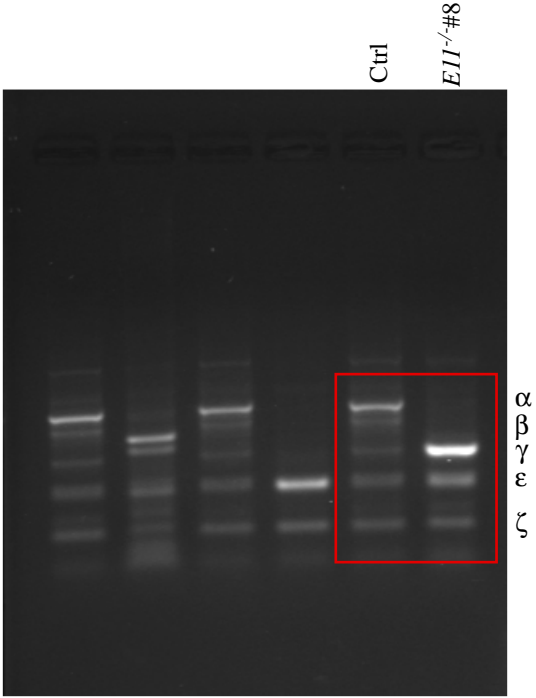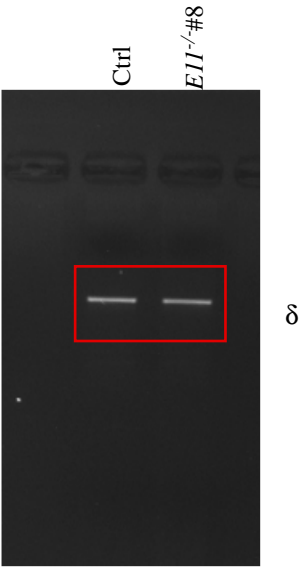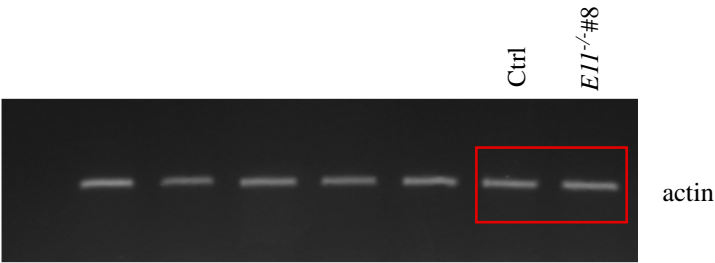

2C

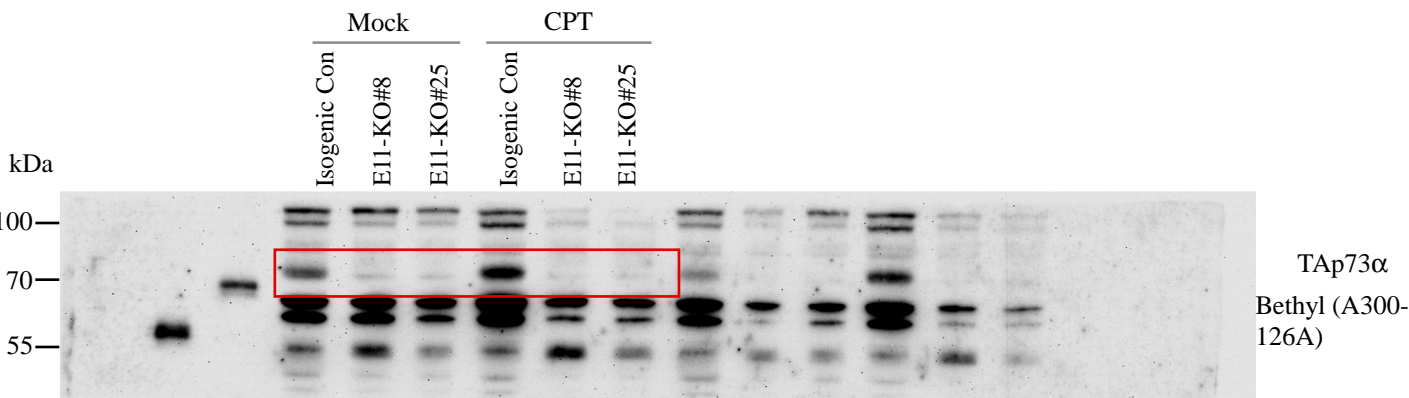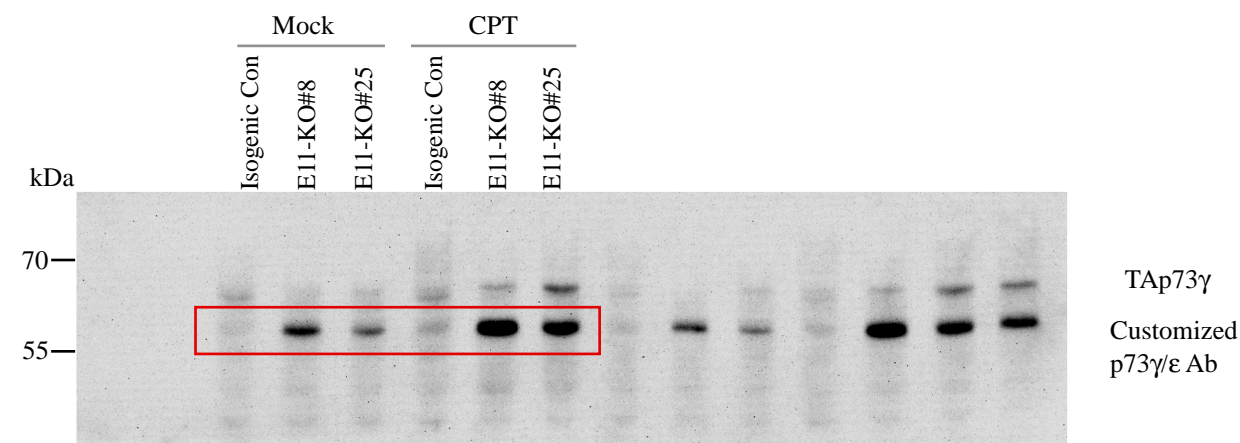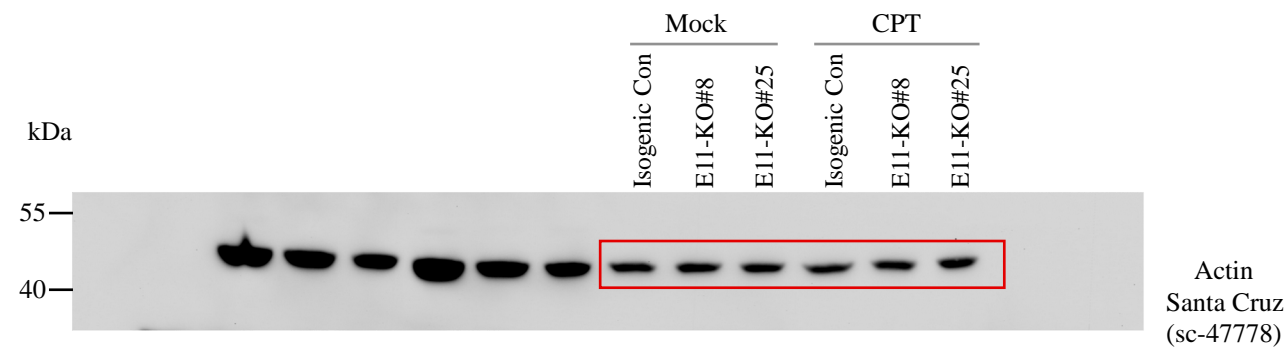

2D

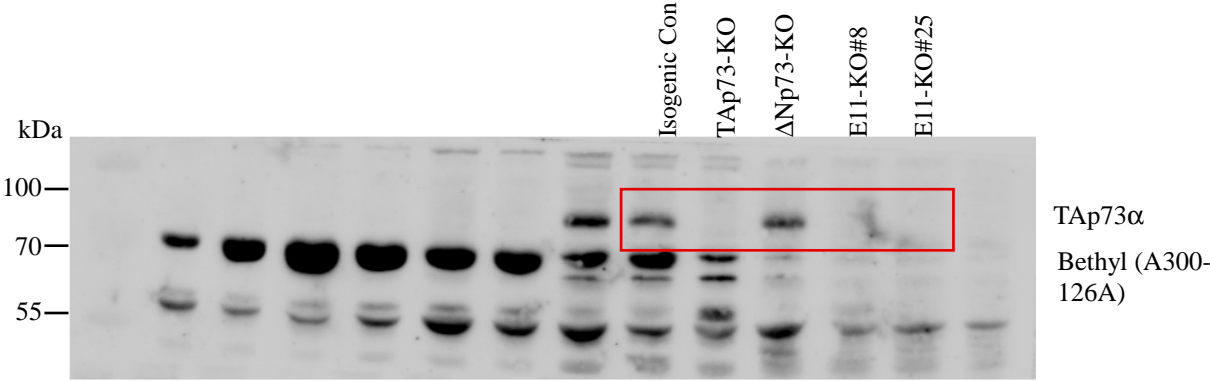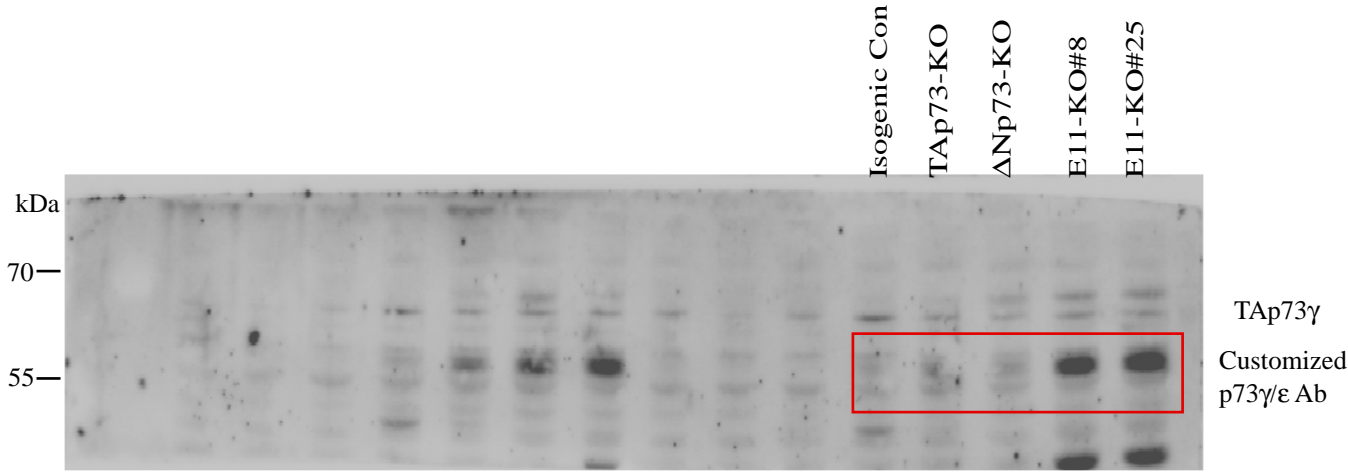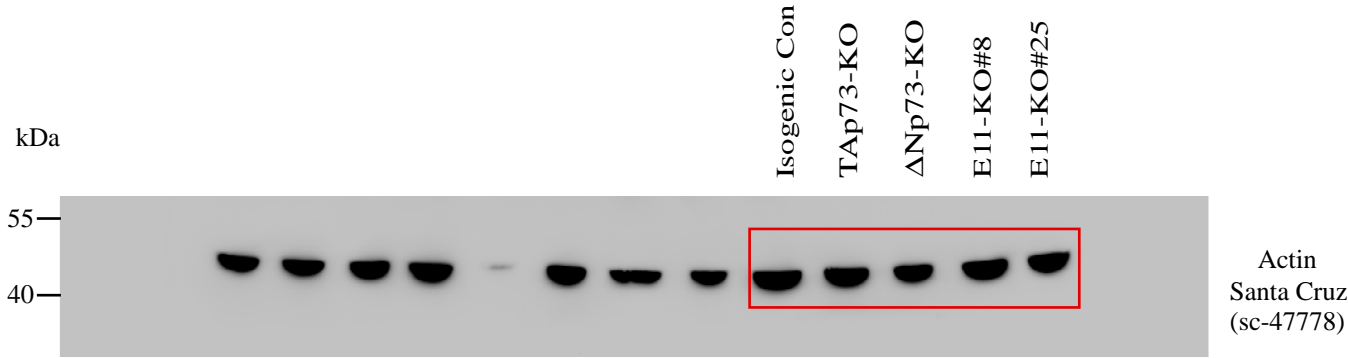

Supplement: Figure 2—source data 1. [file elife-82115-fig2-data1.pdf]

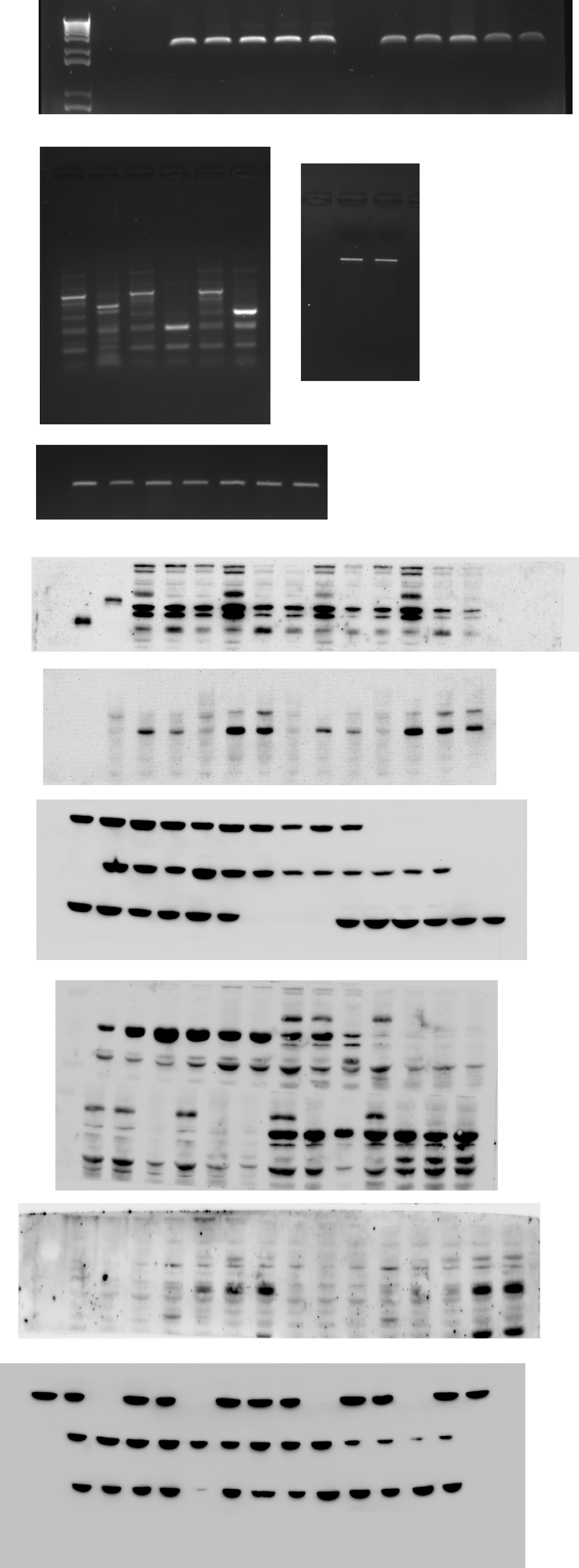

Supplement: Figure 2—source data 2. [file elife-82115-fig2-data2.tif]

1B

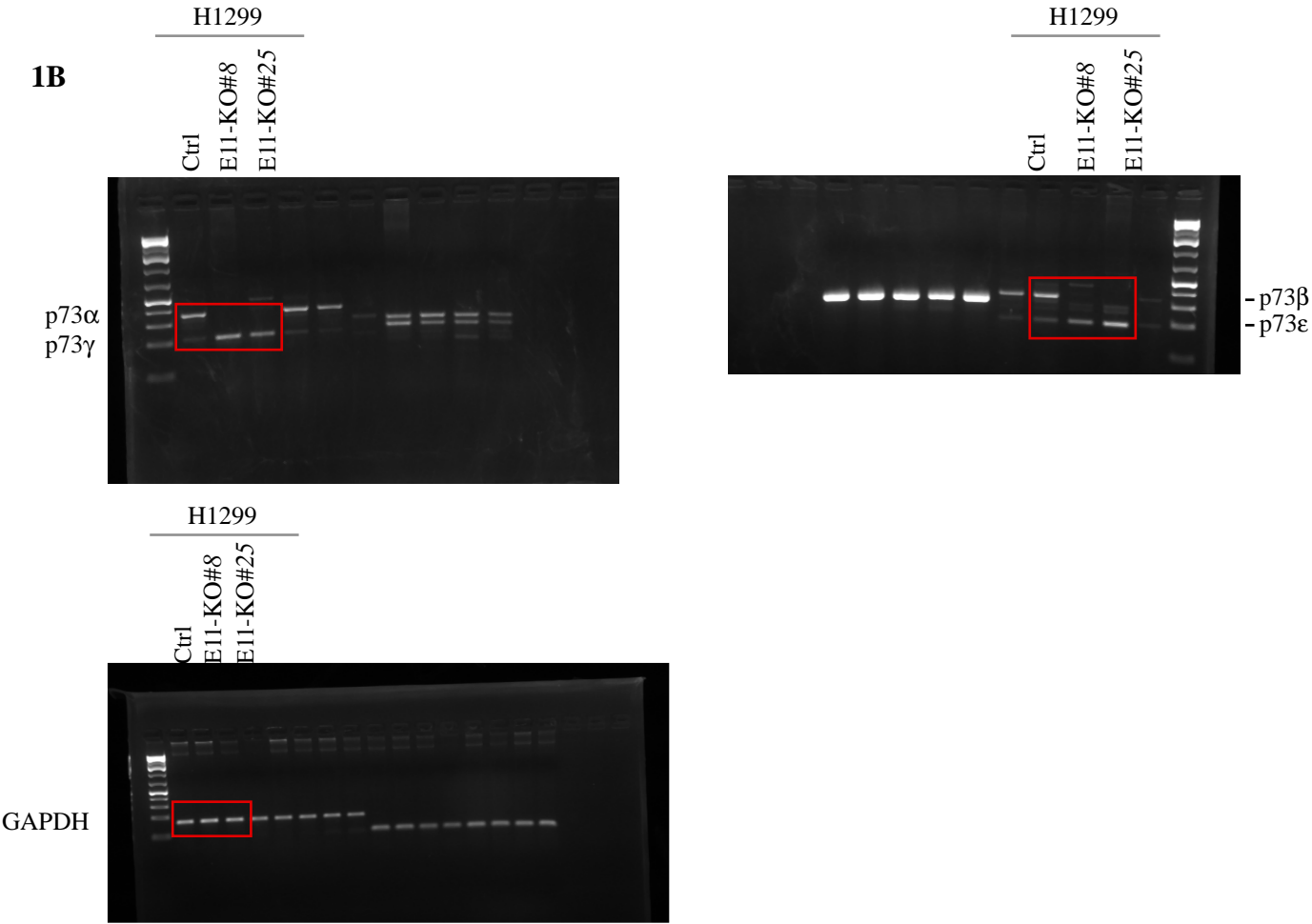

1C

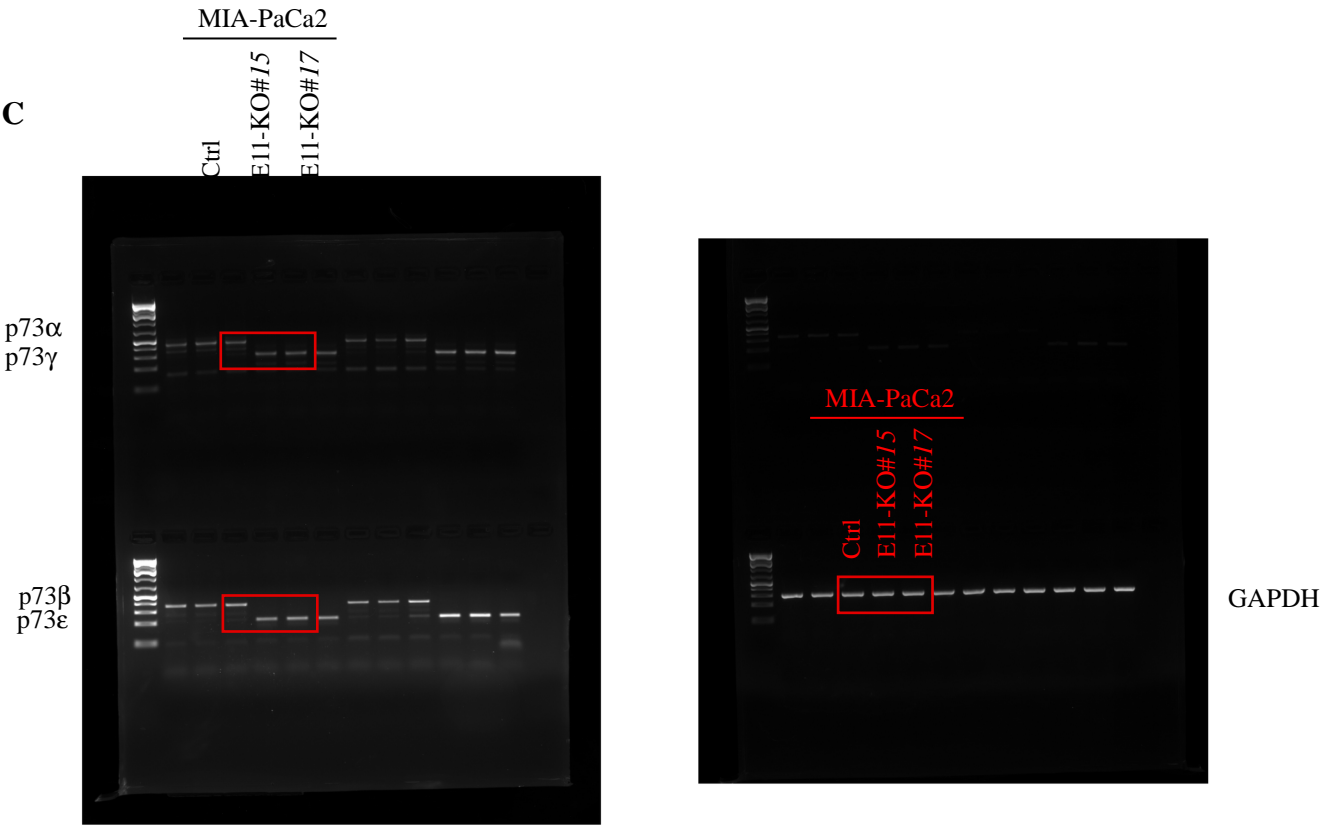

**1D**

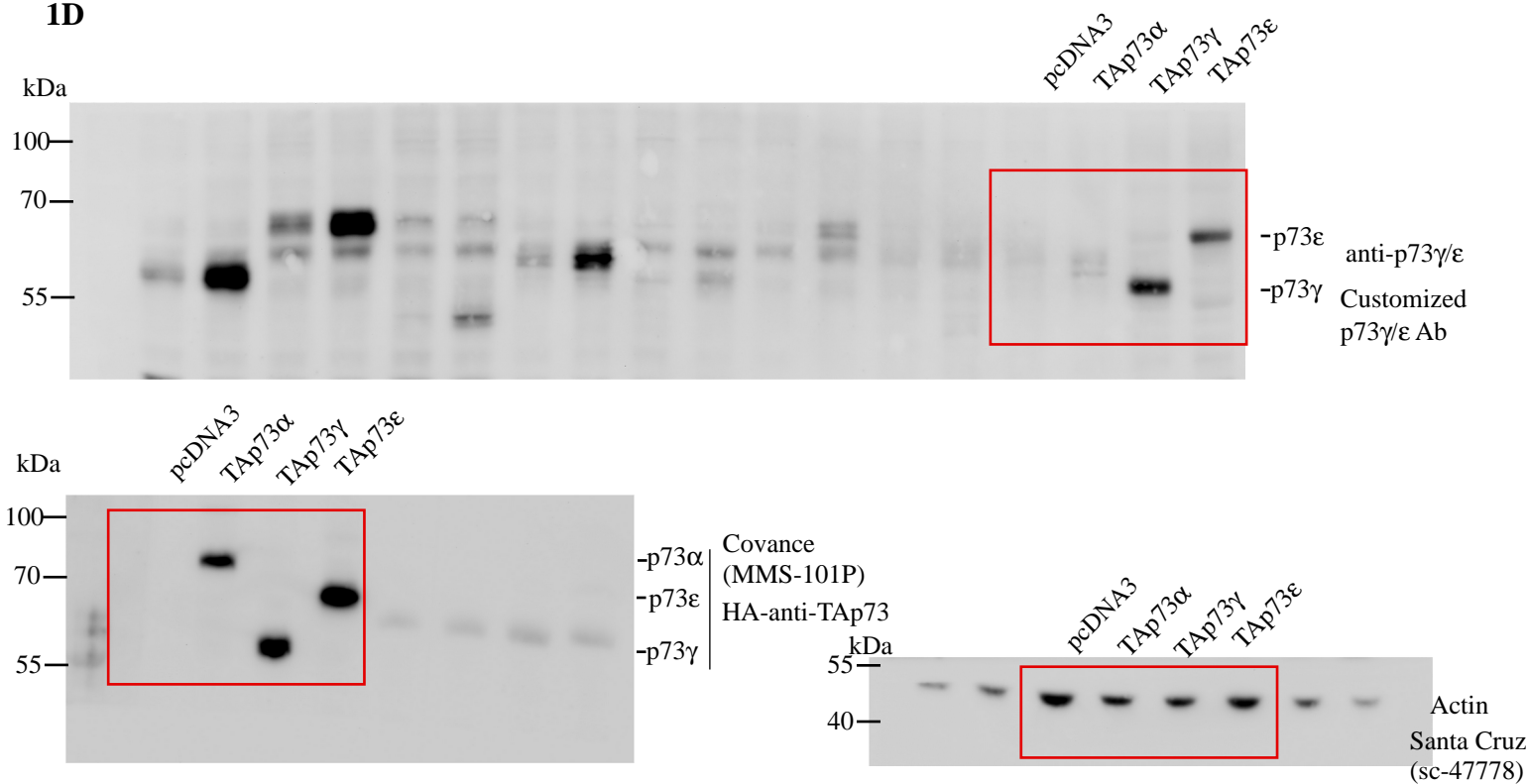

**1E**

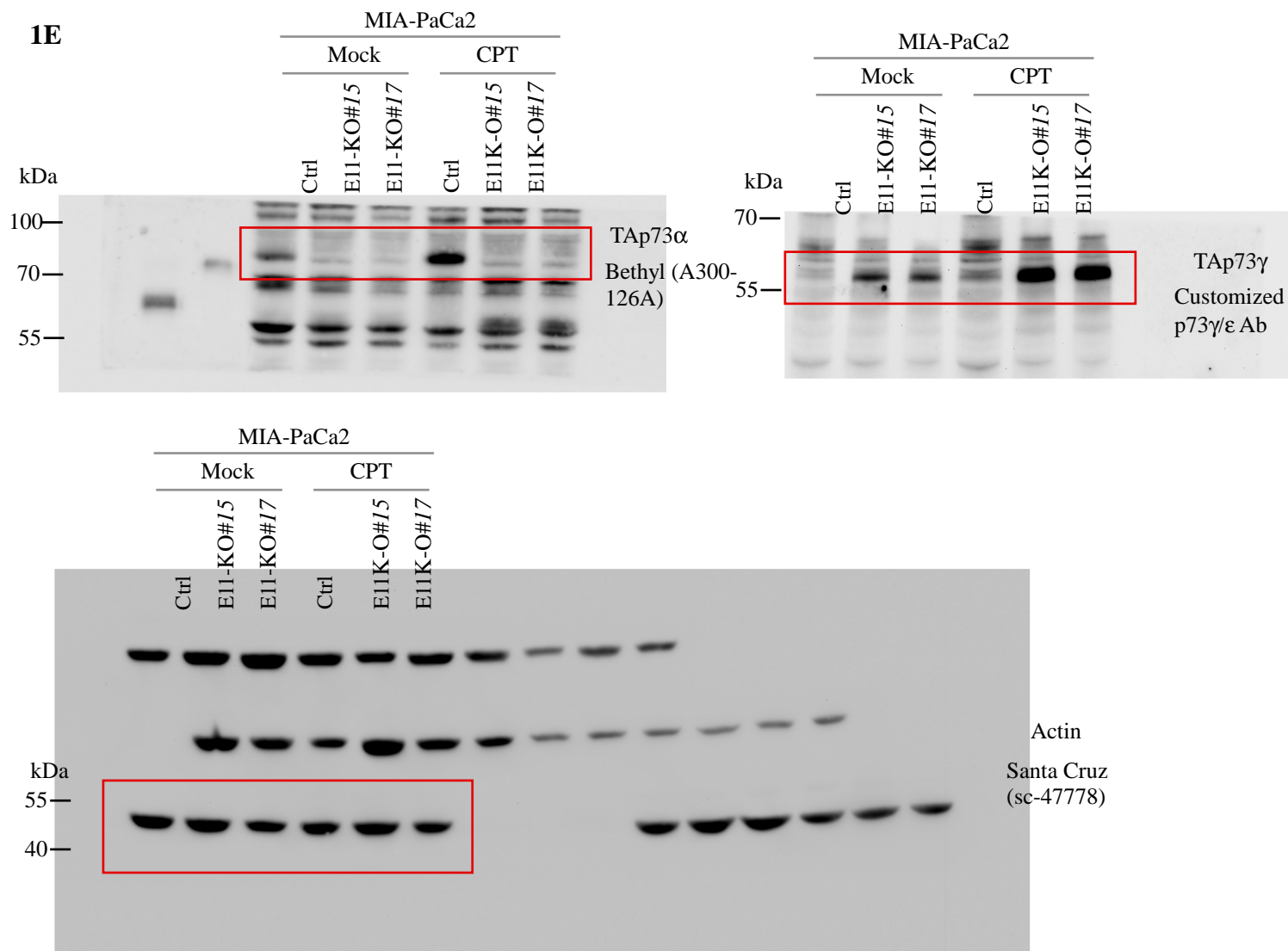

**1F**

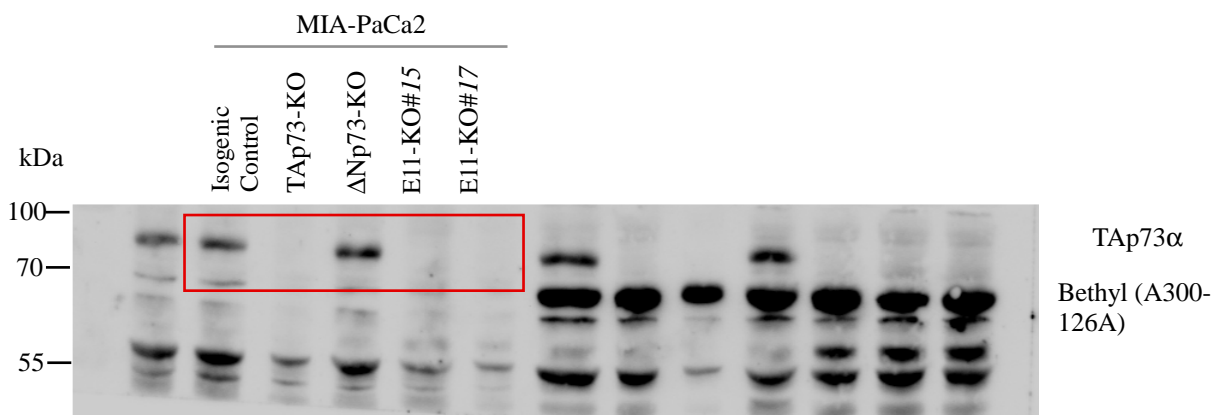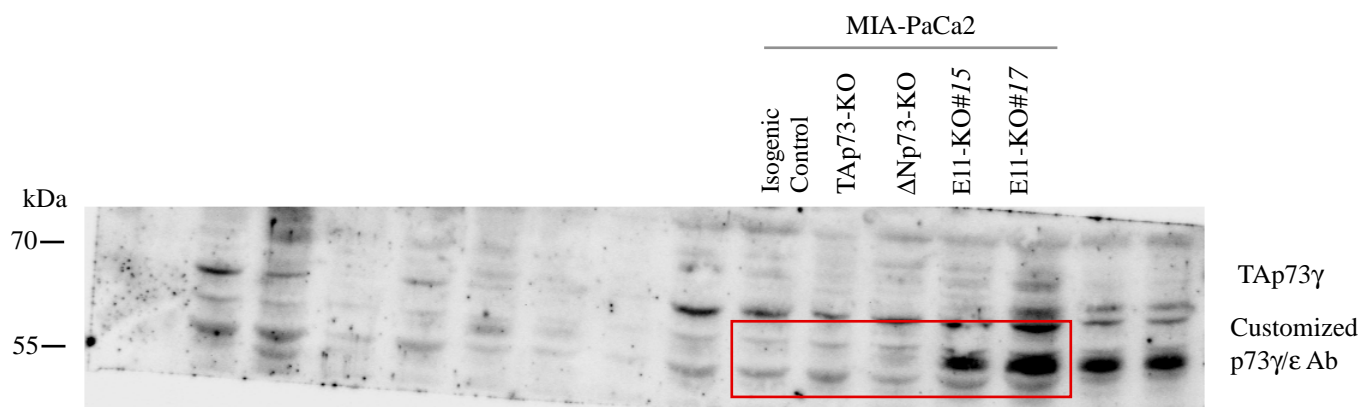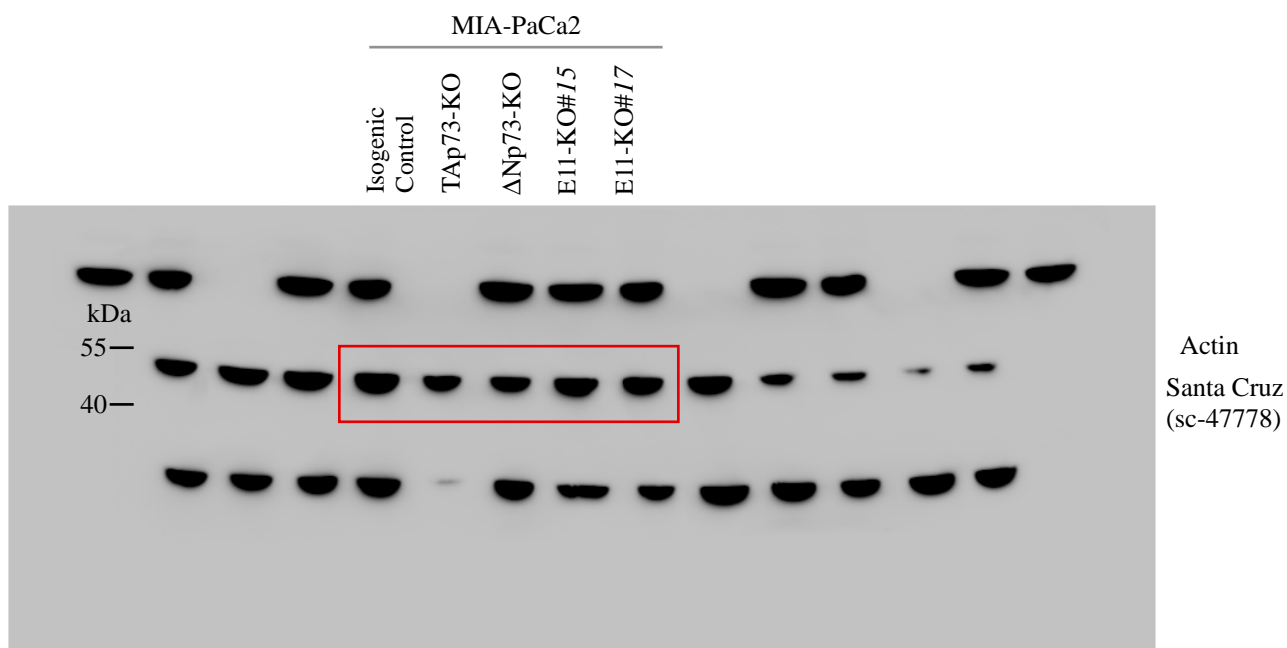

1J

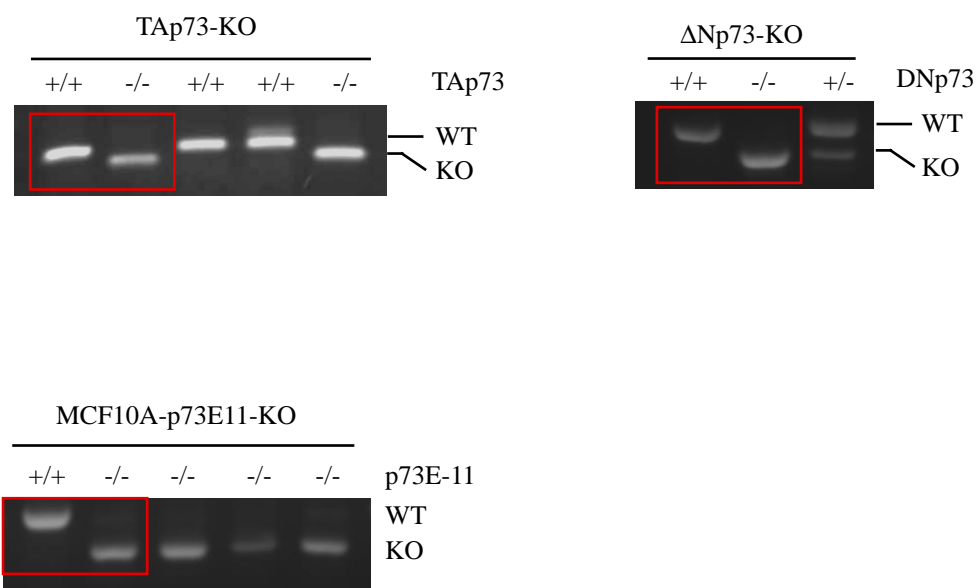

Supplement: Figure 2—figure supplement 1—source data 1. [file elife-82115-fig2-figsupp1-data1.pdf]

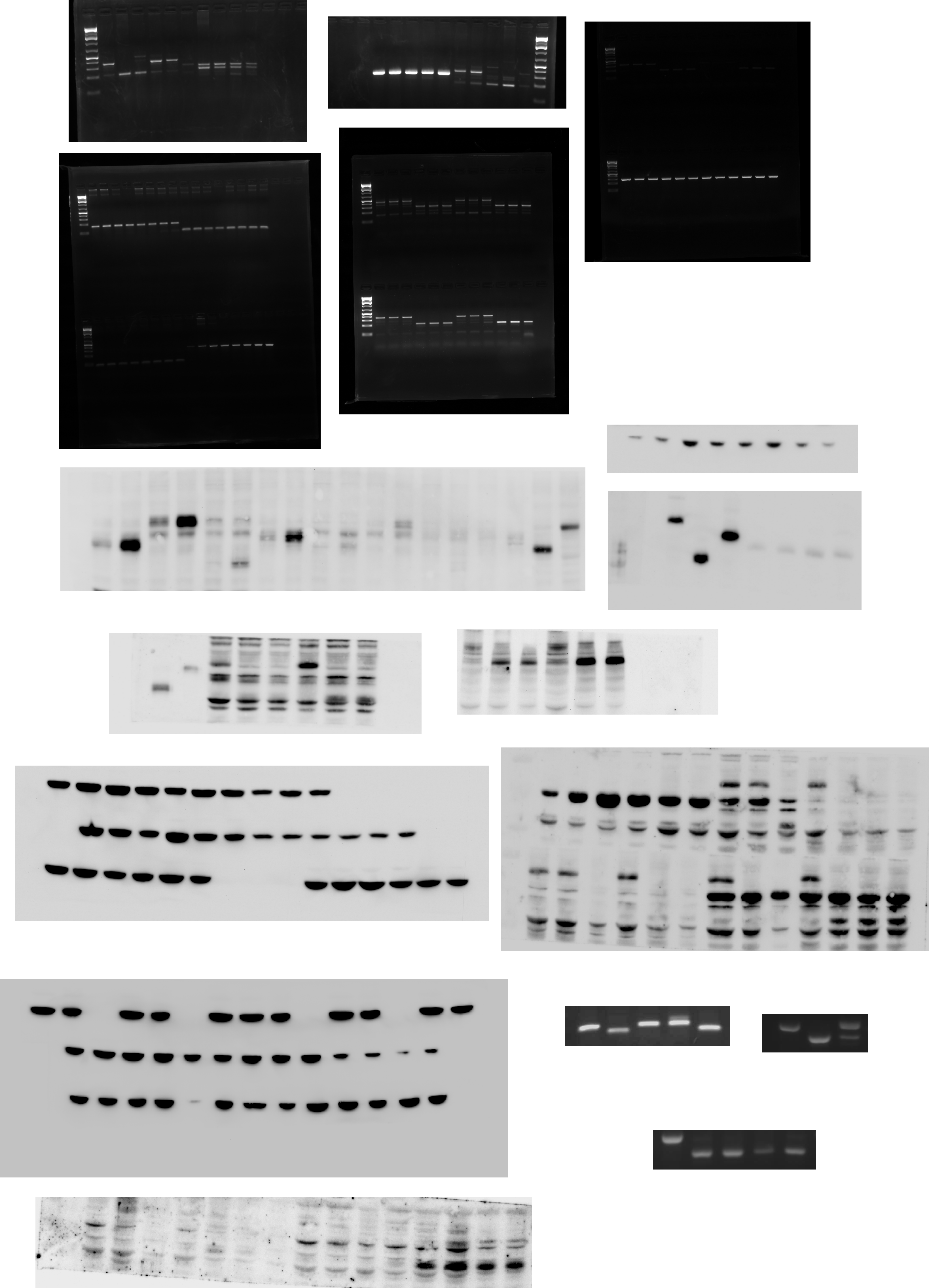

Supplement: Figure 2—figure supplement 1—source data 2. [file elife-82115-fig2-figsupp1-data2.tif]

2A

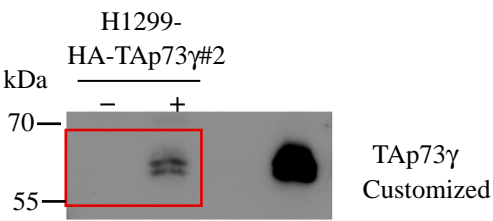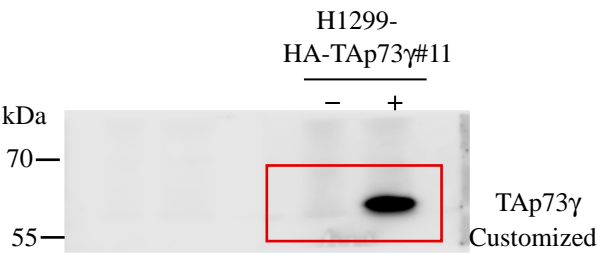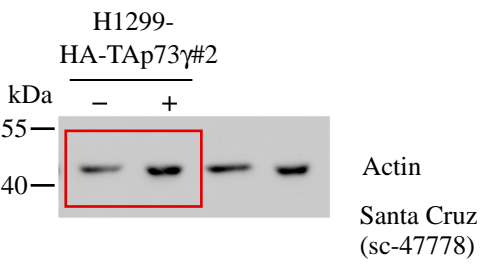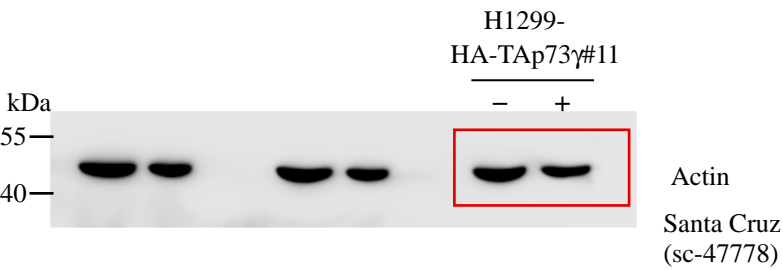

Supplement: Figure 2—figure supplement 2—source data 1. [file elife-82115-fig2-figsupp2-data1.pdf]

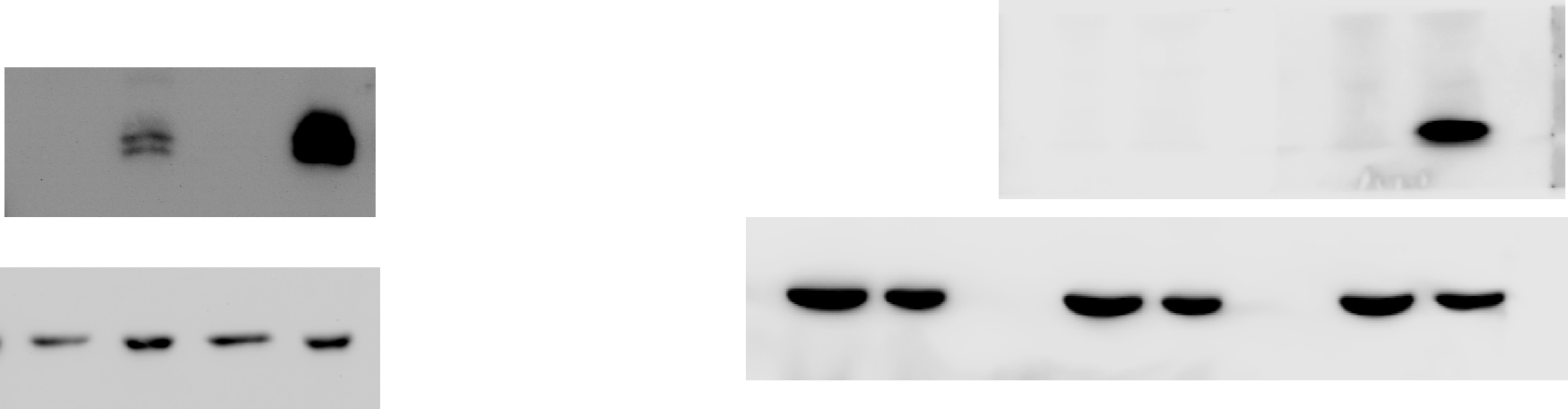

Supplement: Figure 2—figure supplement 2—source data 2. [file elife-82115-fig2-figsupp2-data2.tif]

3A

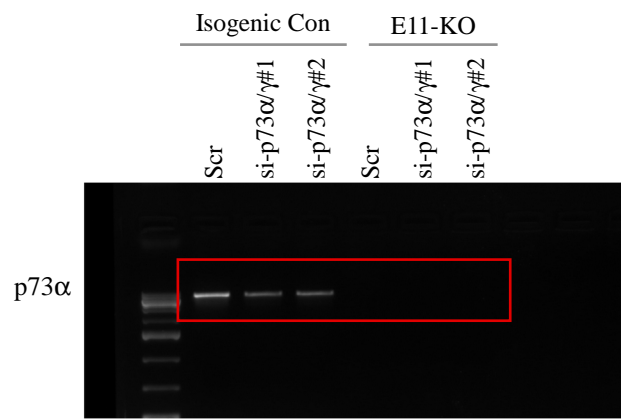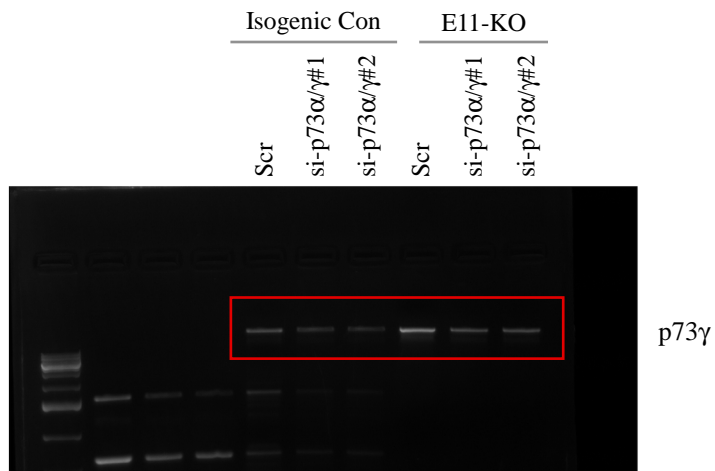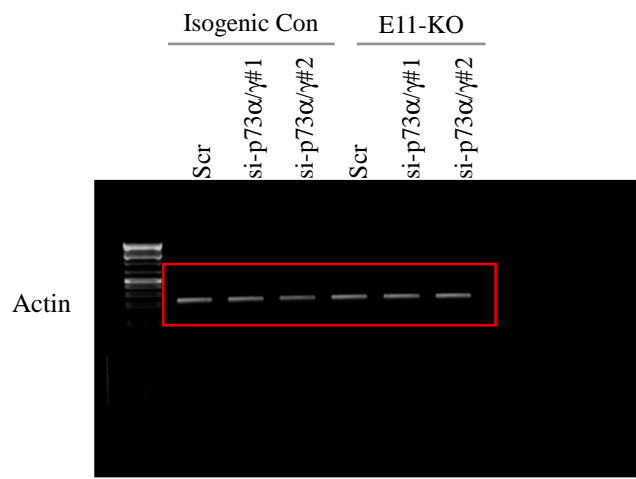

3B

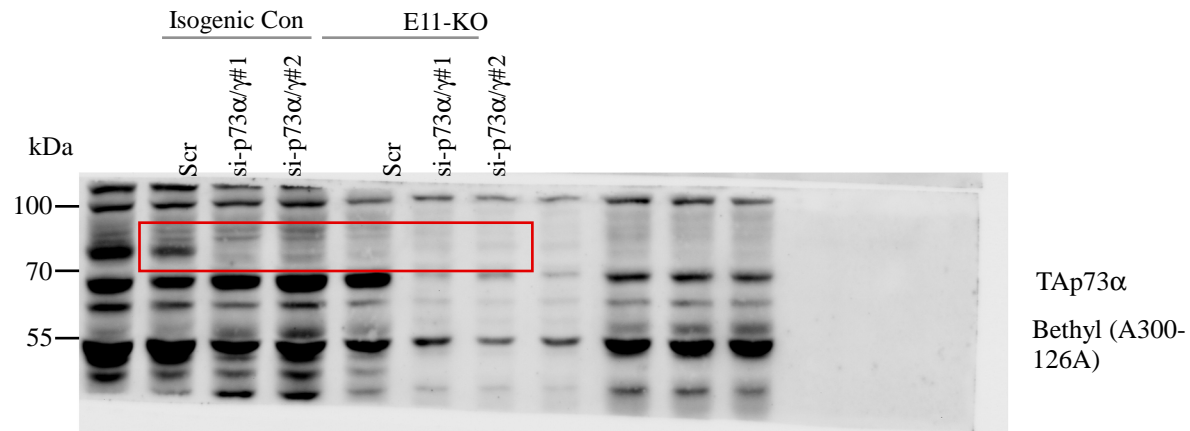

TAp73 $\alpha$   
Bethyl (A300-126A)

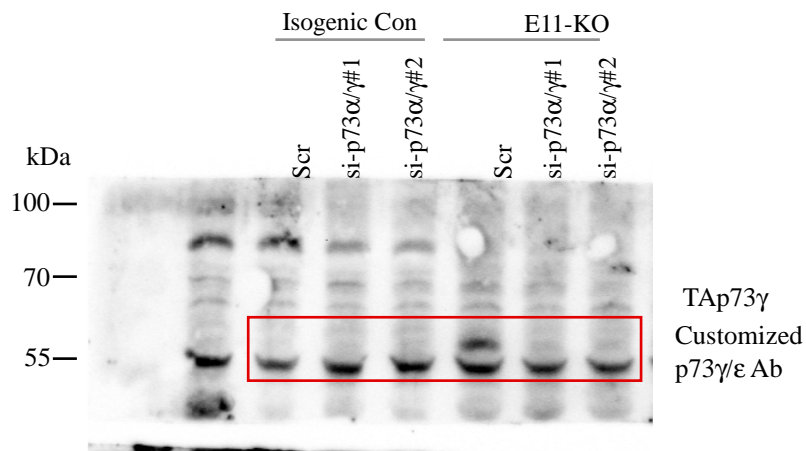

TAp73 $\gamma$   
Customized  
p73 $\gamma$ /ε Ab

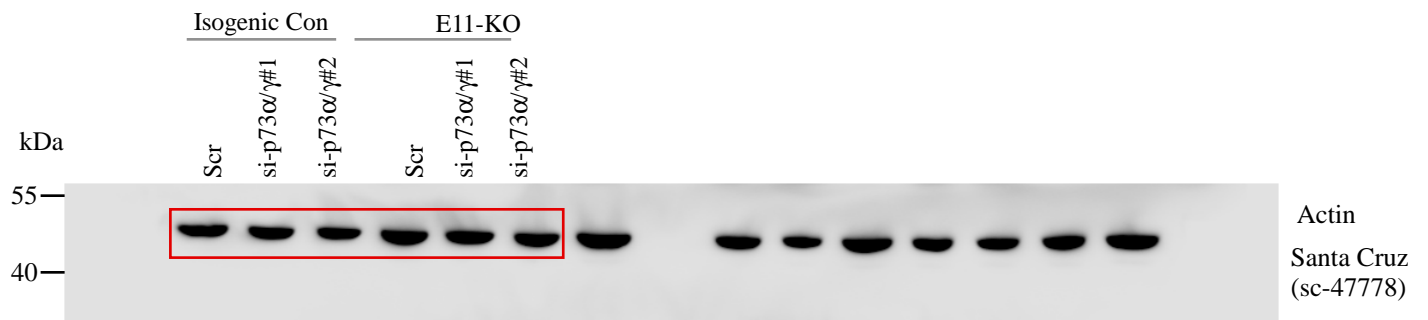

Actin  
Santa Cruz  
(sc-47778)

3D

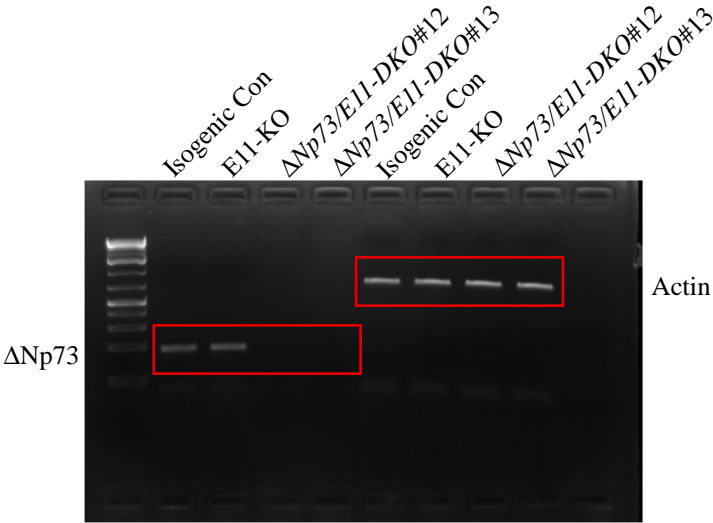

3E

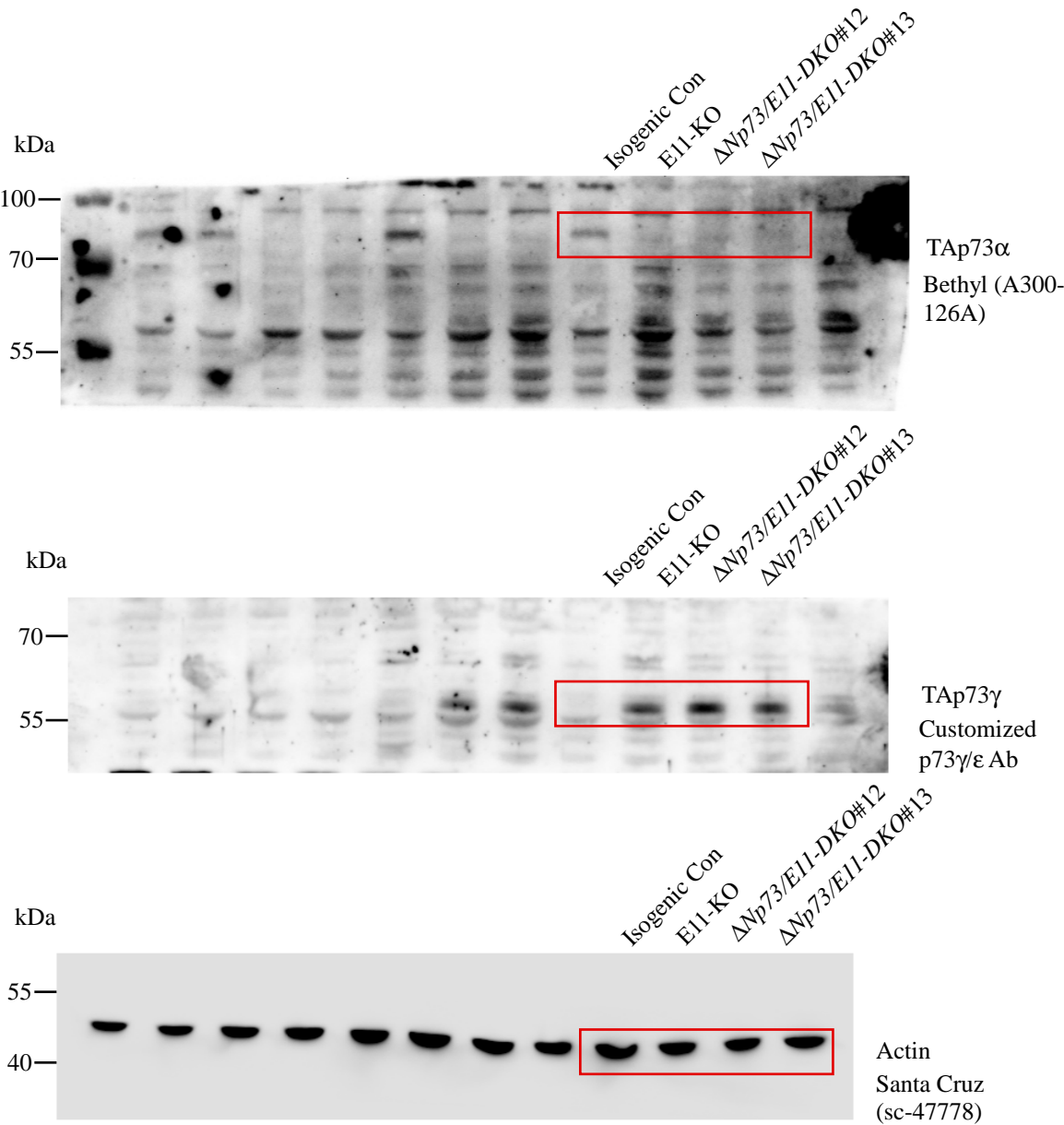

Supplement: Figure 3—source data 1. [file elife-82115-fig3-data1.pdf]

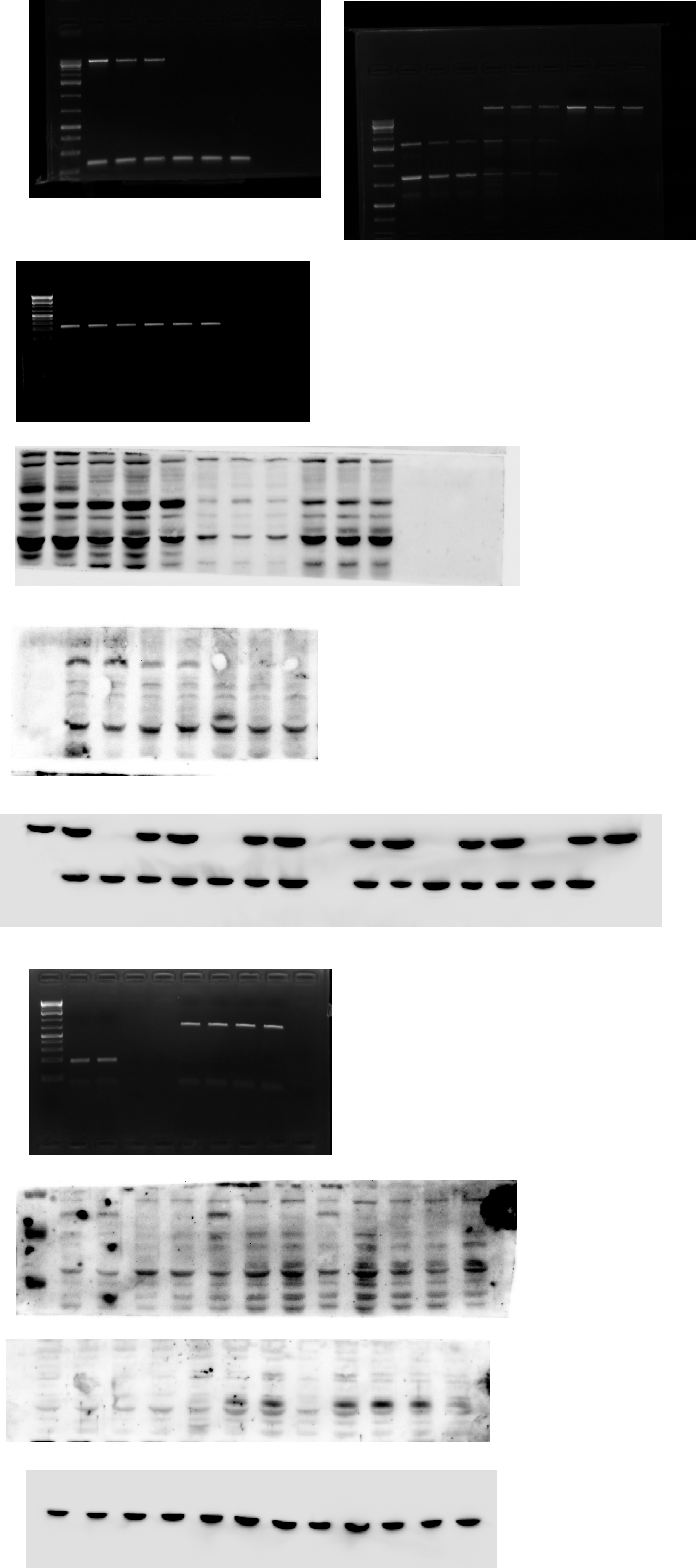

Supplement: Figure 3—source data 2. [file elife-82115-fig3-data2.tif]

3D

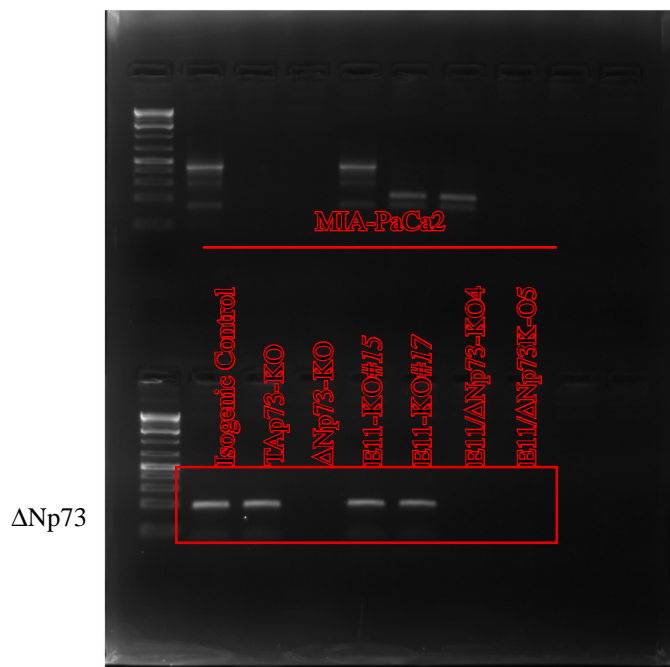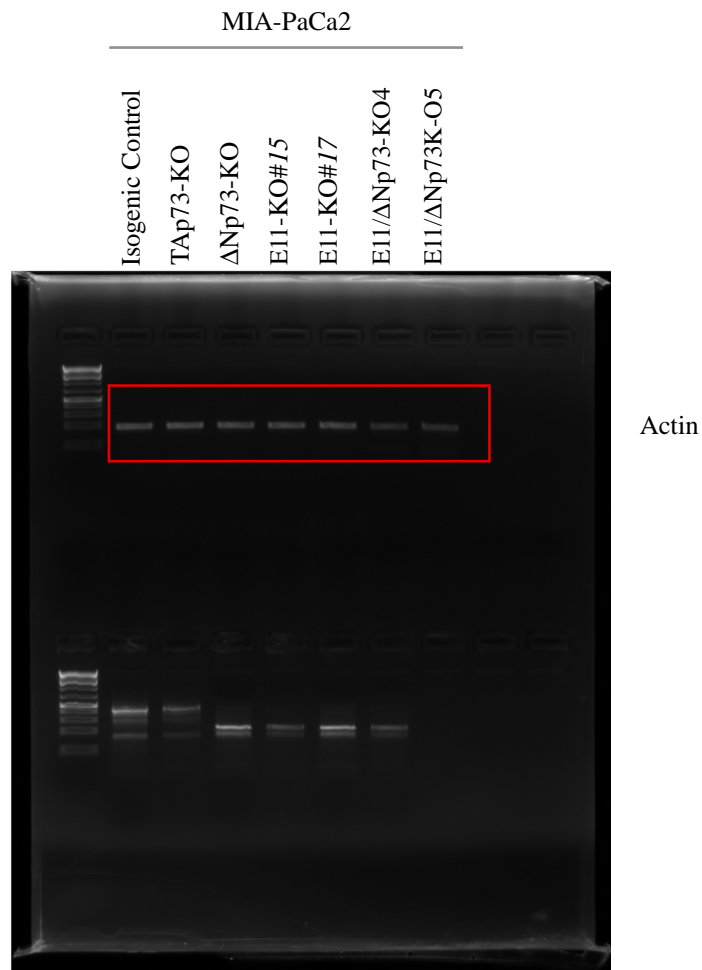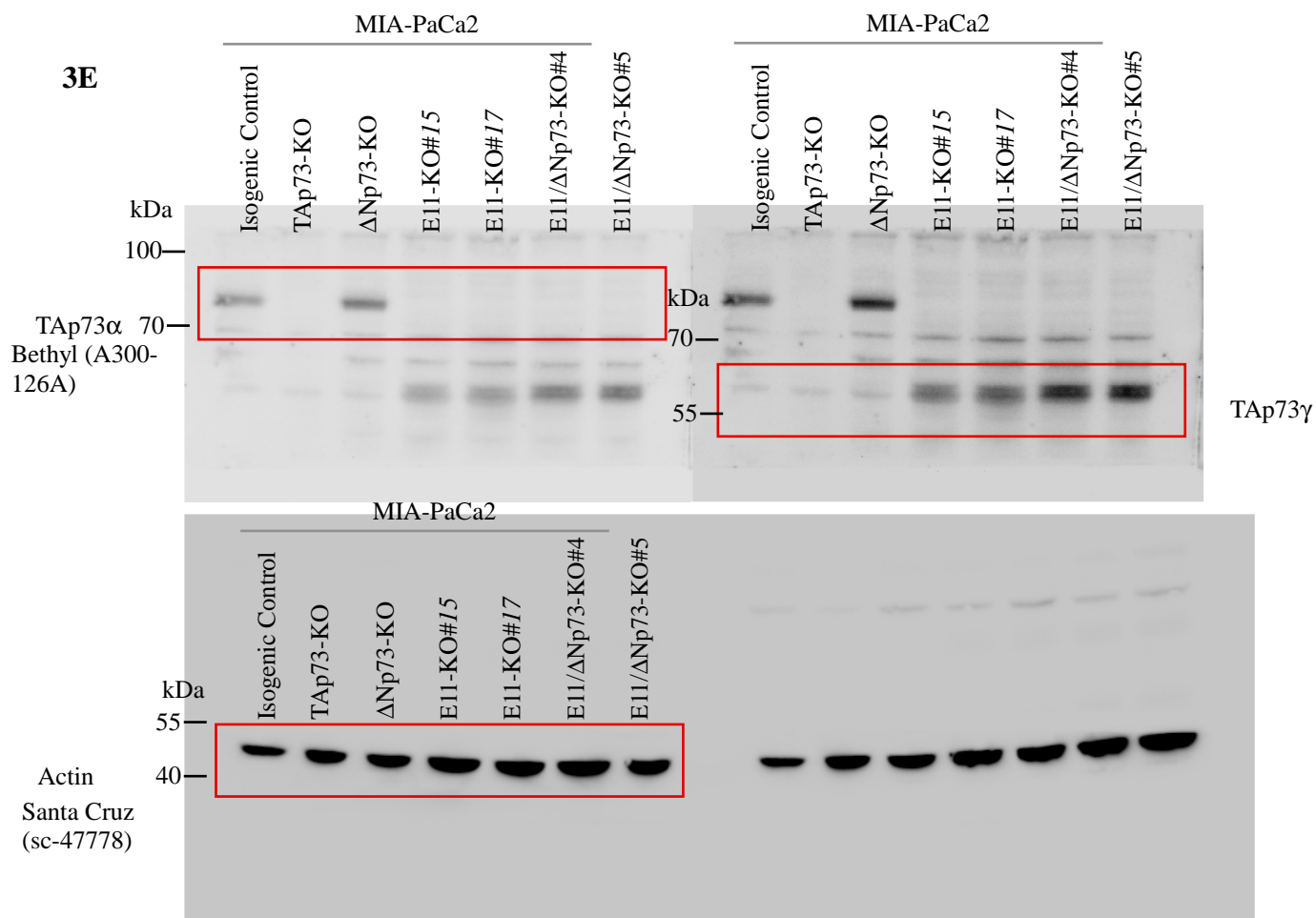

Supplement: Figure 3—figure supplement 1—source data 1. [file elife-82115-fig3-figsupp1-data1.pdf]

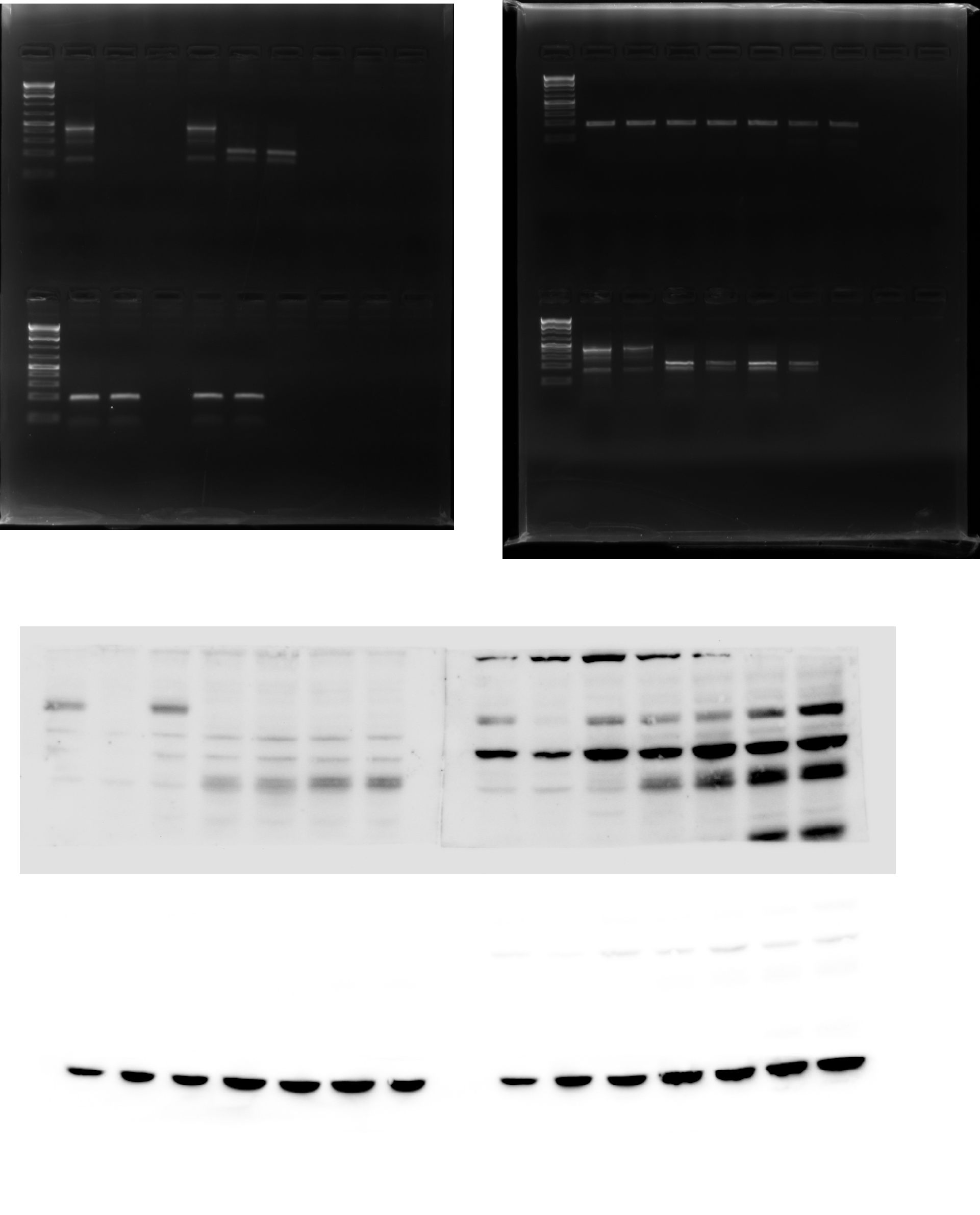

Supplement: Figure 3—figure supplement 1—source data 2. [file elife-82115-fig3-figsupp1-data2.tif]

4A

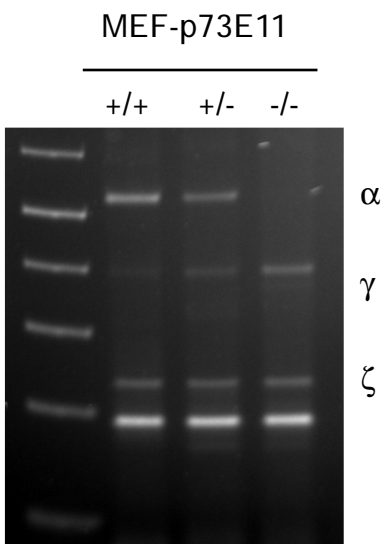

4C

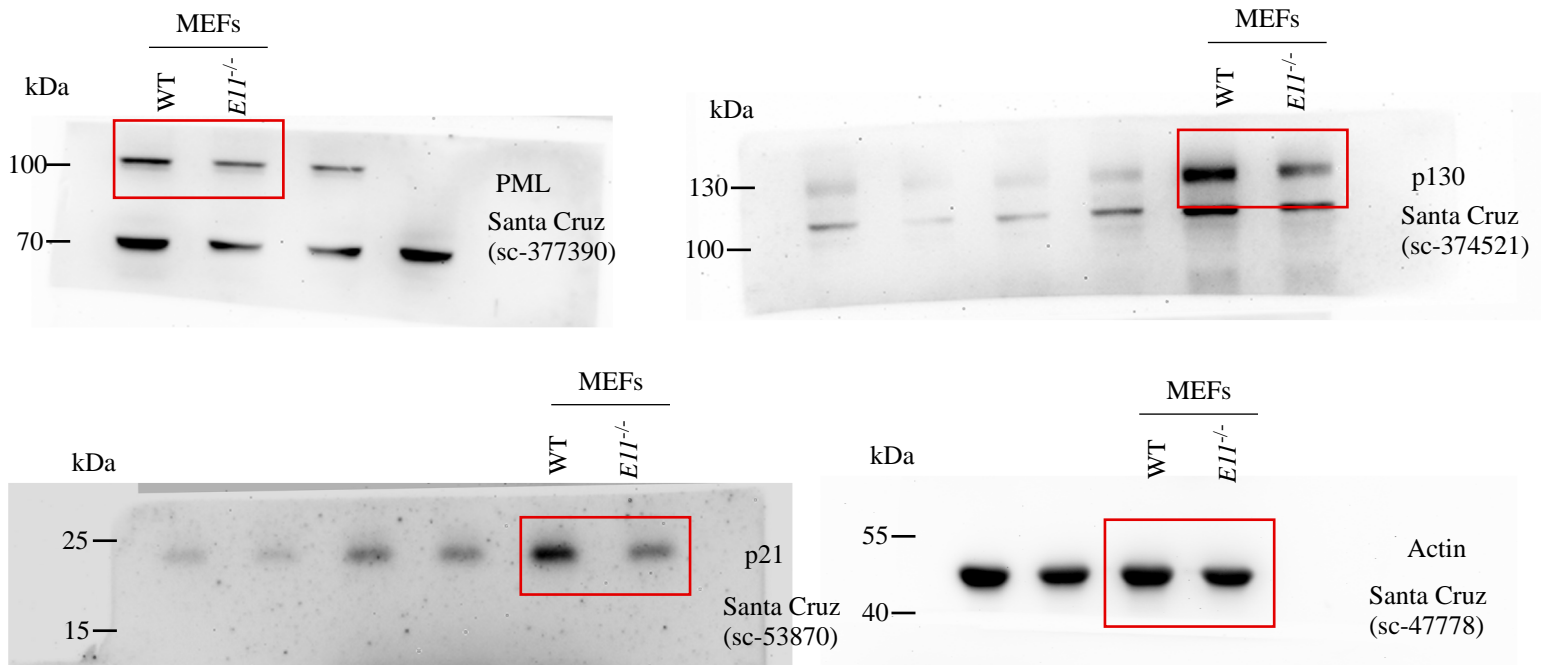

Supplement: Figure 4—source data 1. [file elife-82115-fig4-data1.pdf]

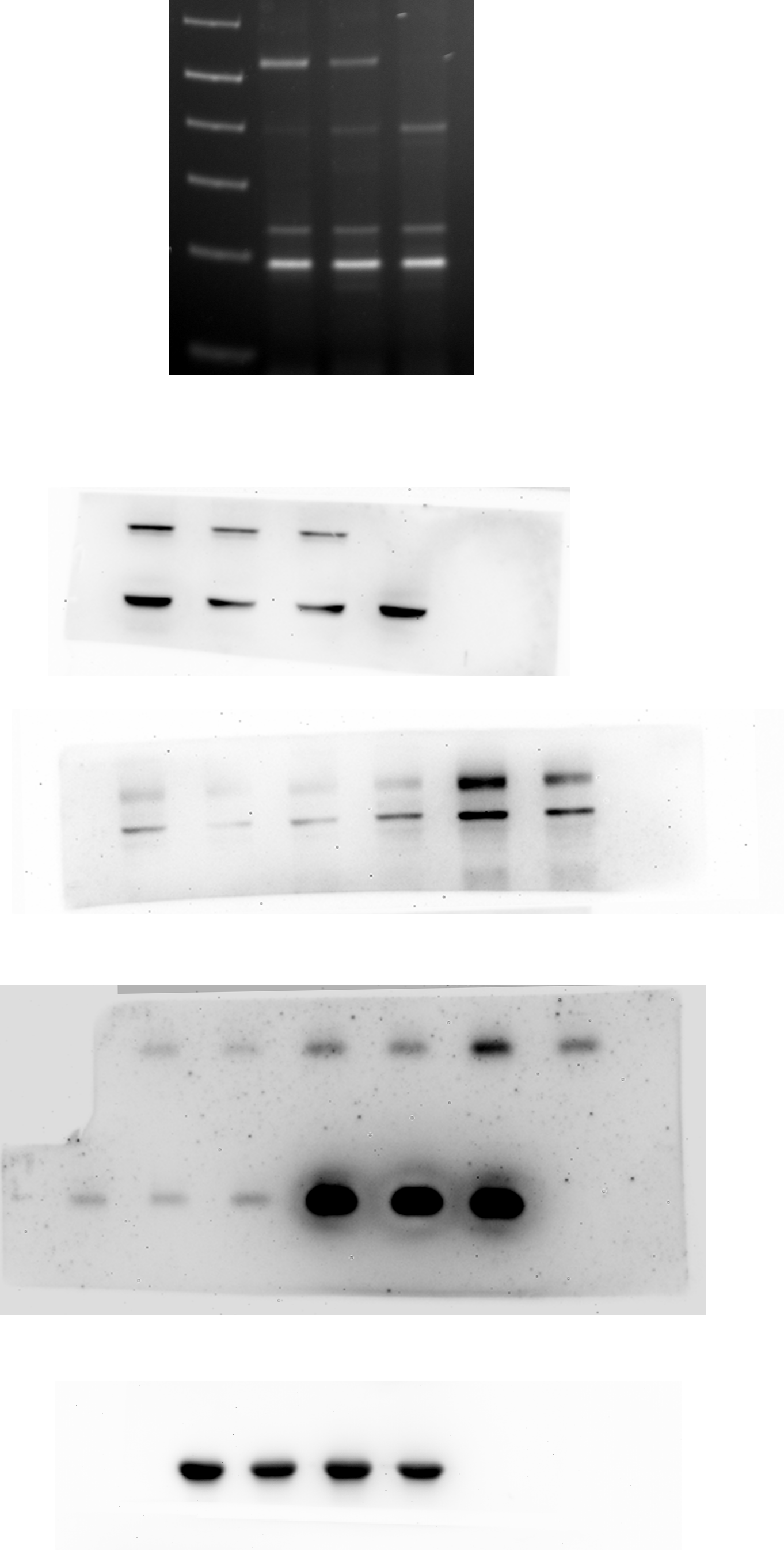

Supplement: Figure 4—source data 2. [file elife-82115-fig4-data2.tif]

4B

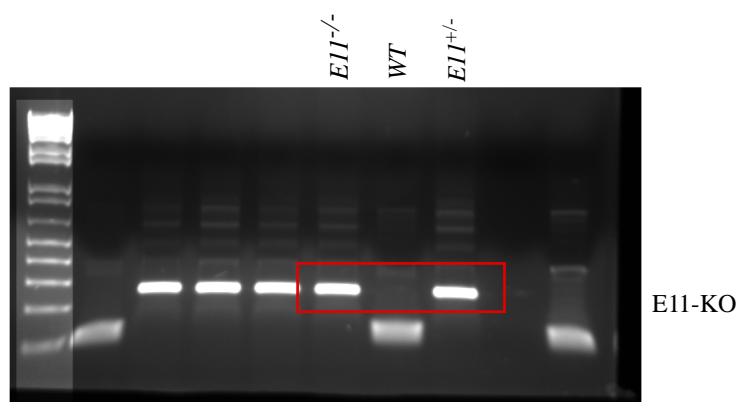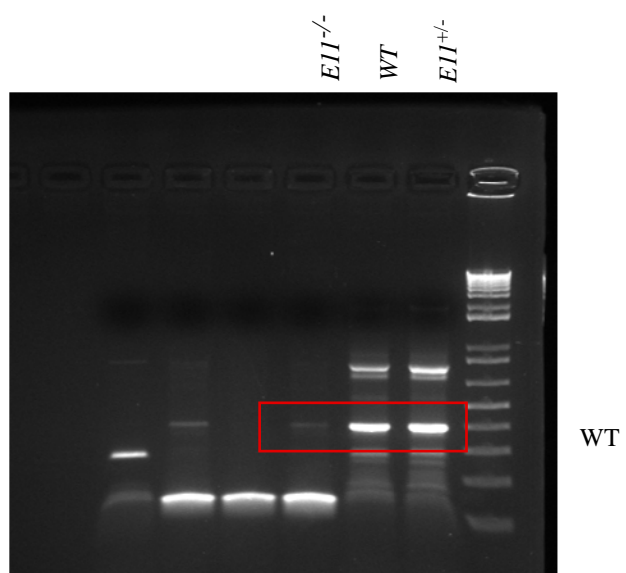

Supplement: Figure 4—figure supplement 1—source data 1. [file elife-82115-fig4-figsupp1-data1.pdf]

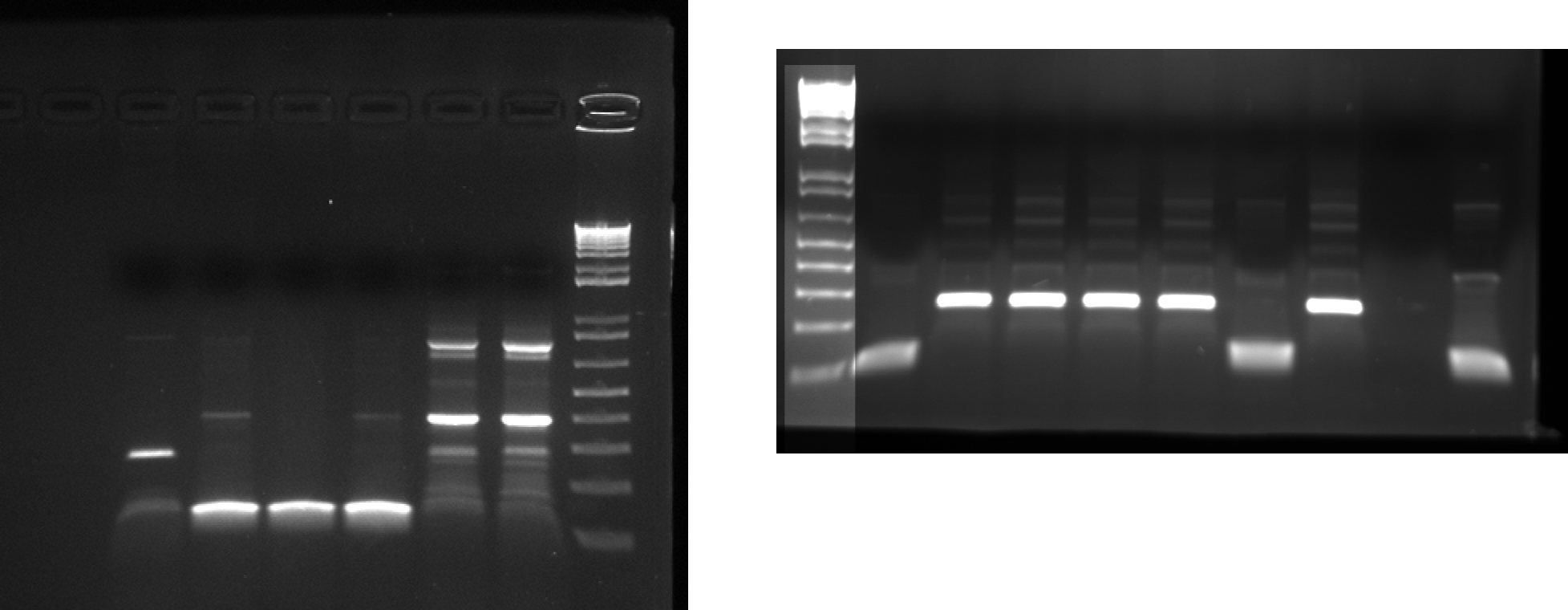

Supplement: Figure 4—figure supplement 1—source data 2. [file elife-82115-fig4-figsupp1-data2.tif]

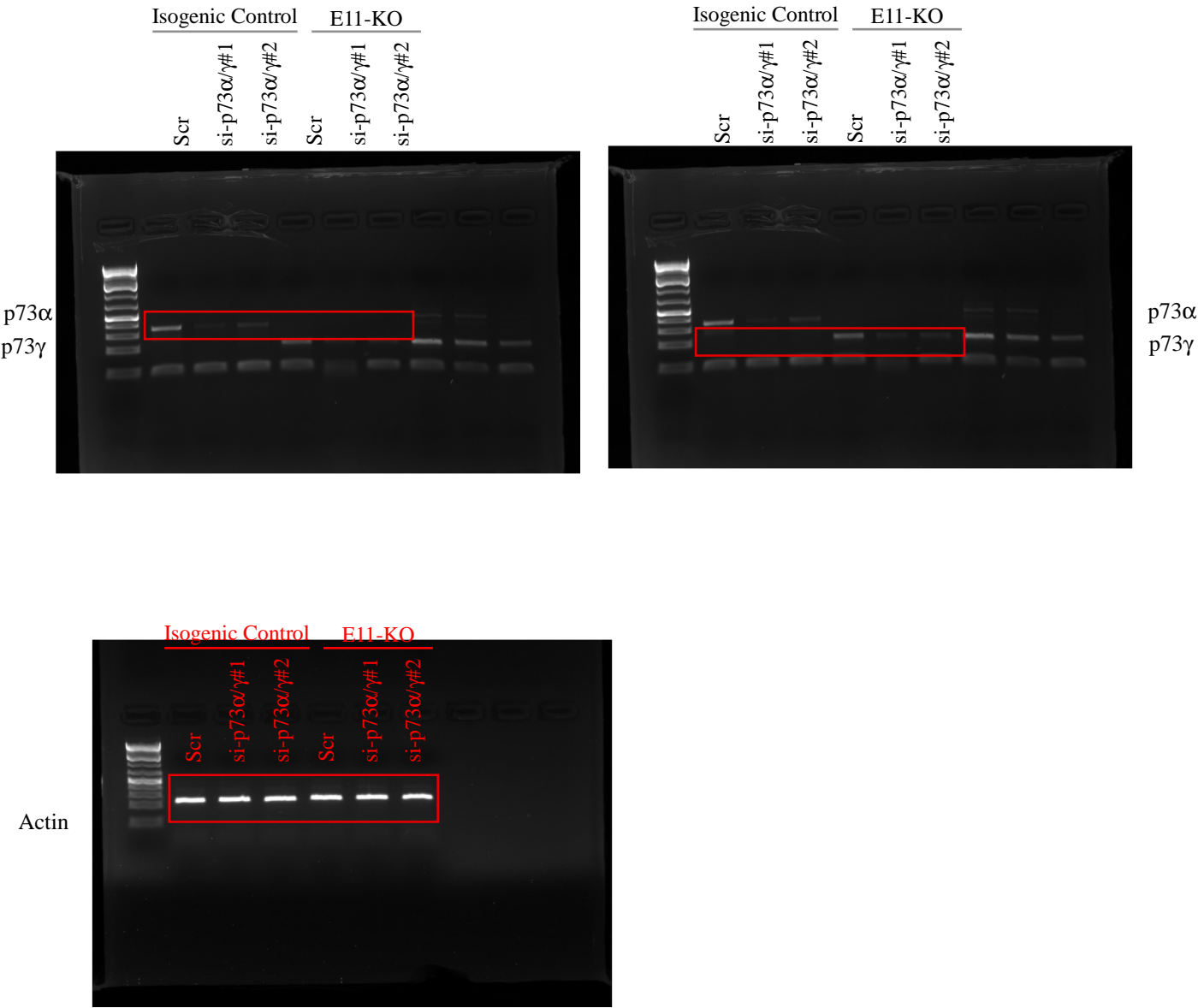

Supplement: Figure 5—source data 1. [file elife-82115-fig5-data1.pdf]

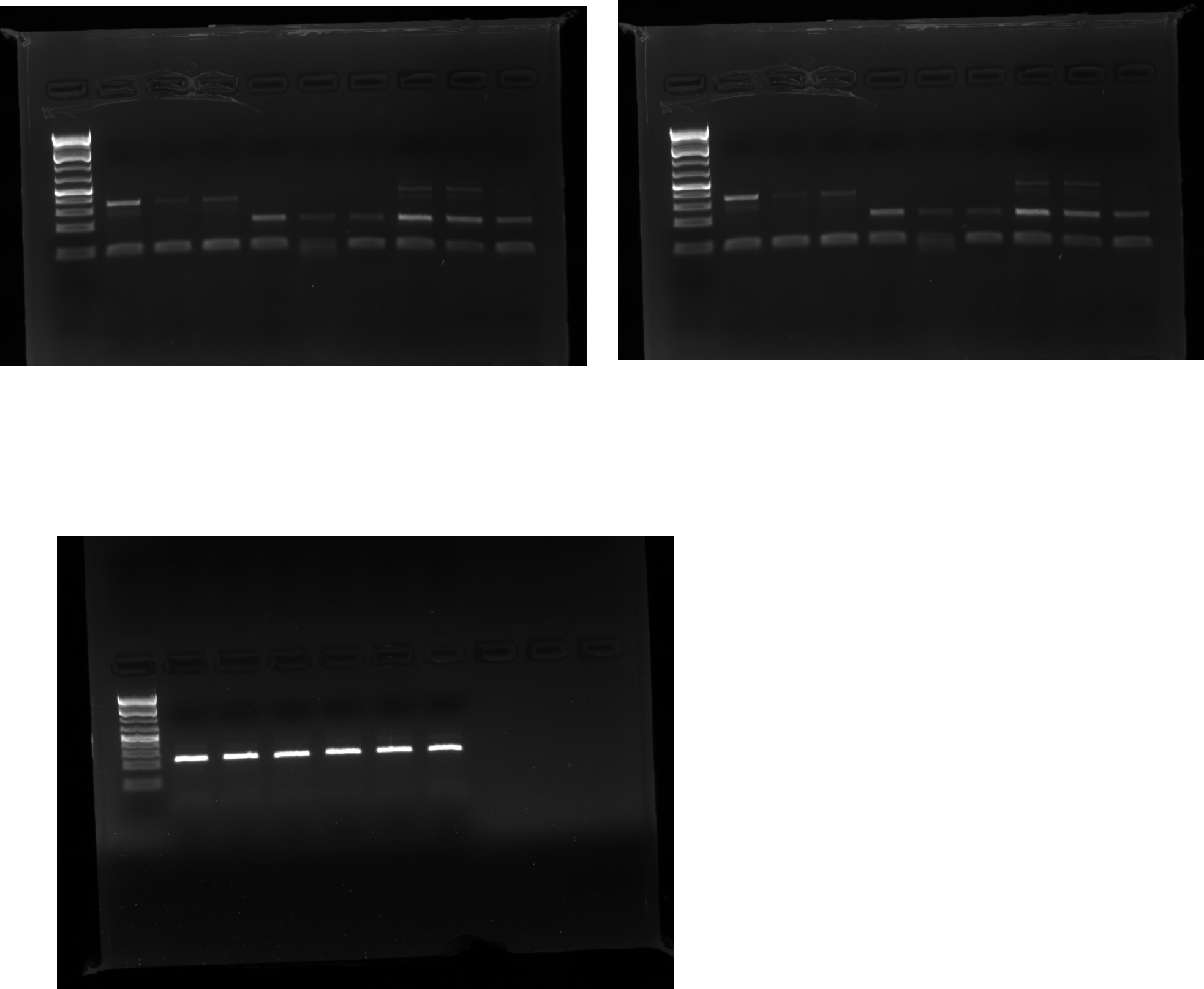

Supplement: Figure 5—source data 2. [file elife-82115-fig5-data2.tif]

5A

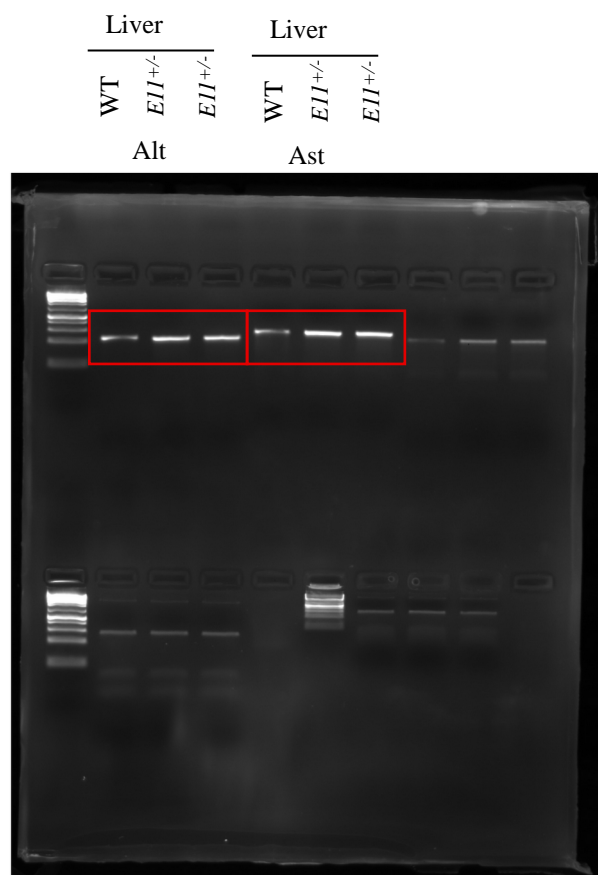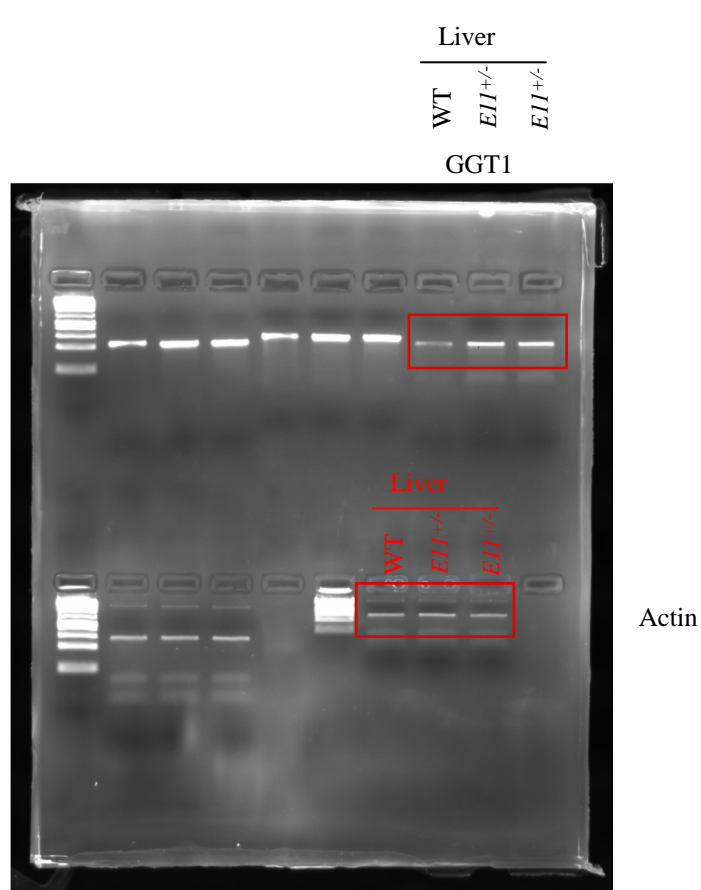

5B

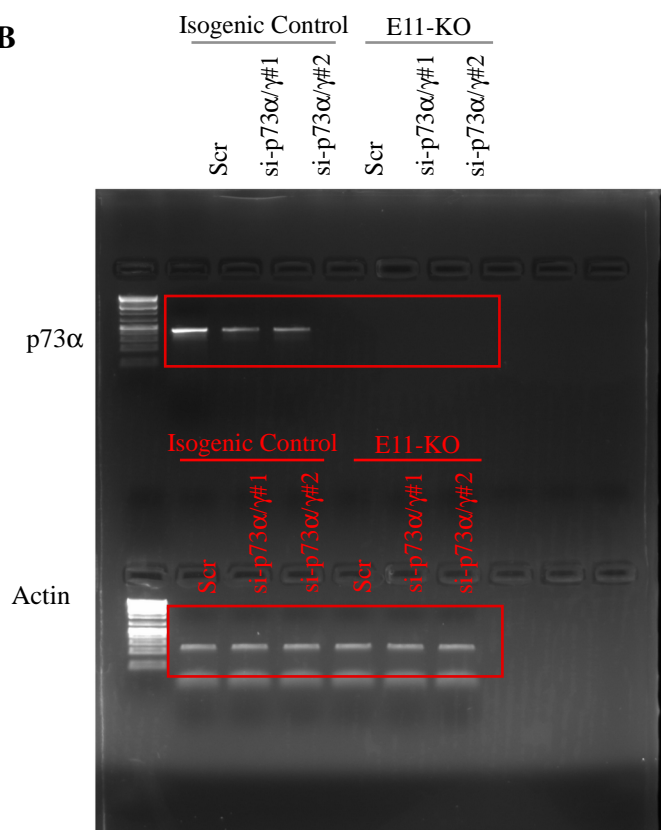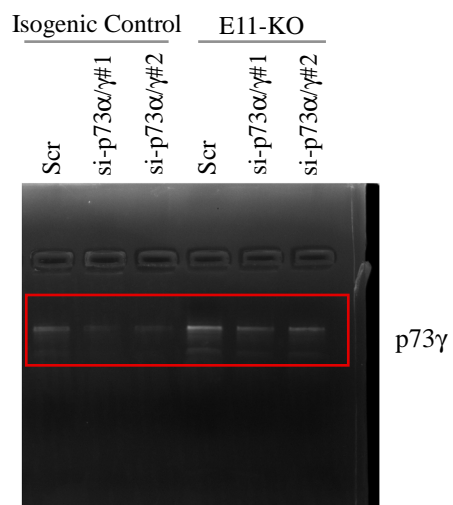

Supplement: Figure 5—figure supplement 1—source data 1. [file elife-82115-fig5-figsupp1-data1.pdf]

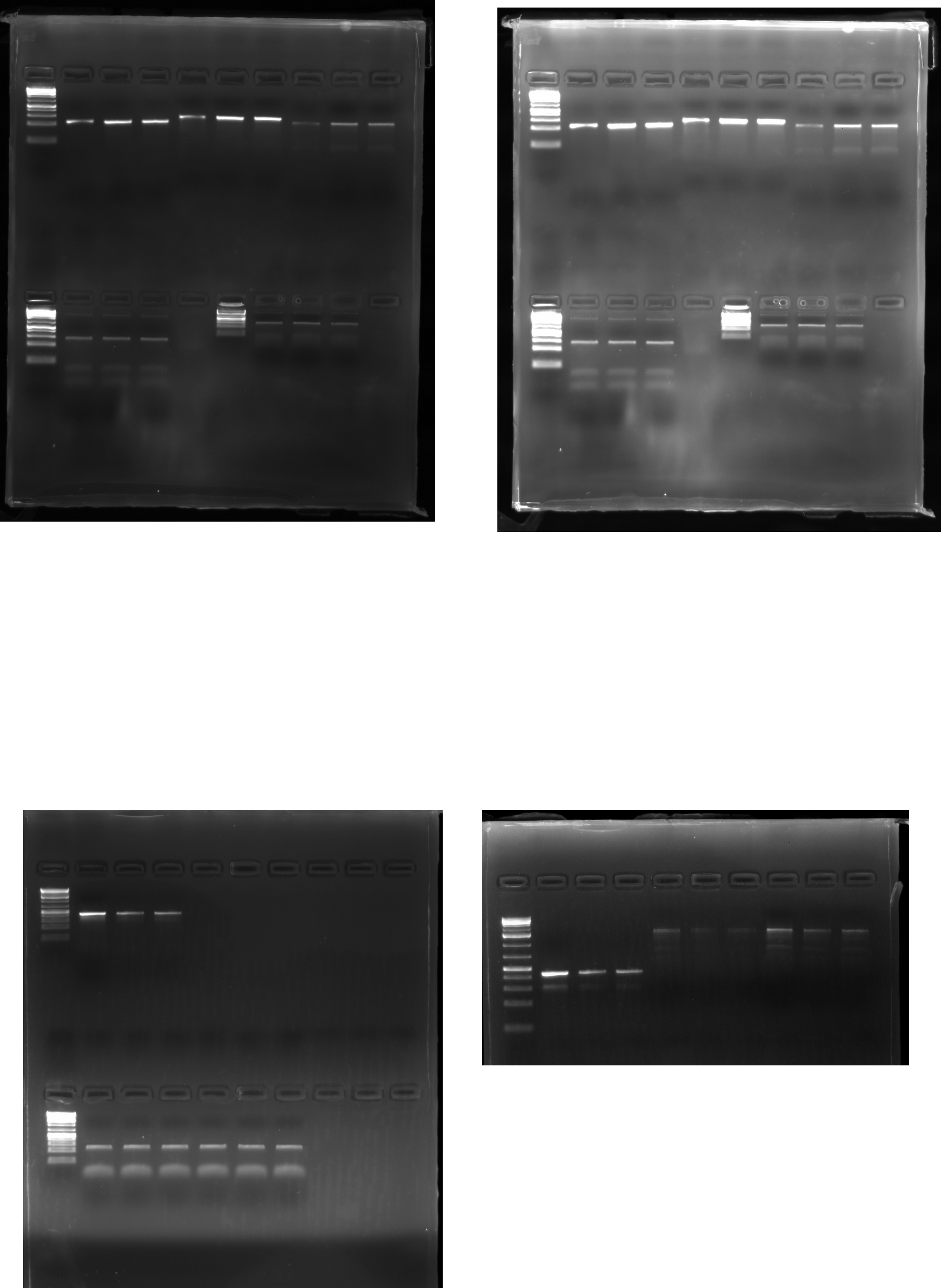

Supplement: Figure 5—figure supplement 1—source data 2. [file elife-82115-fig5-figsupp1-data2.tif]

6A

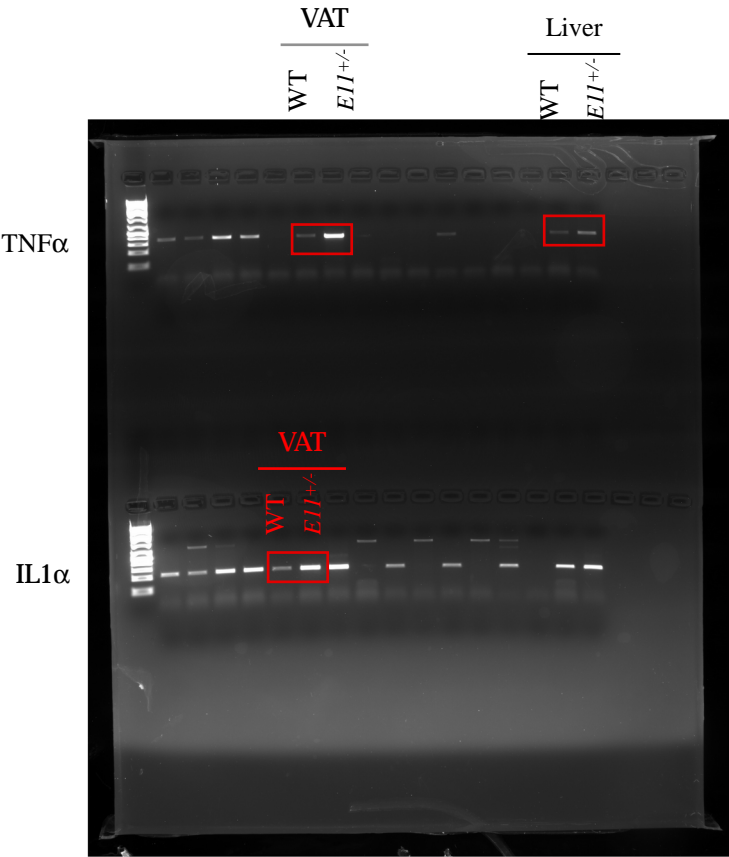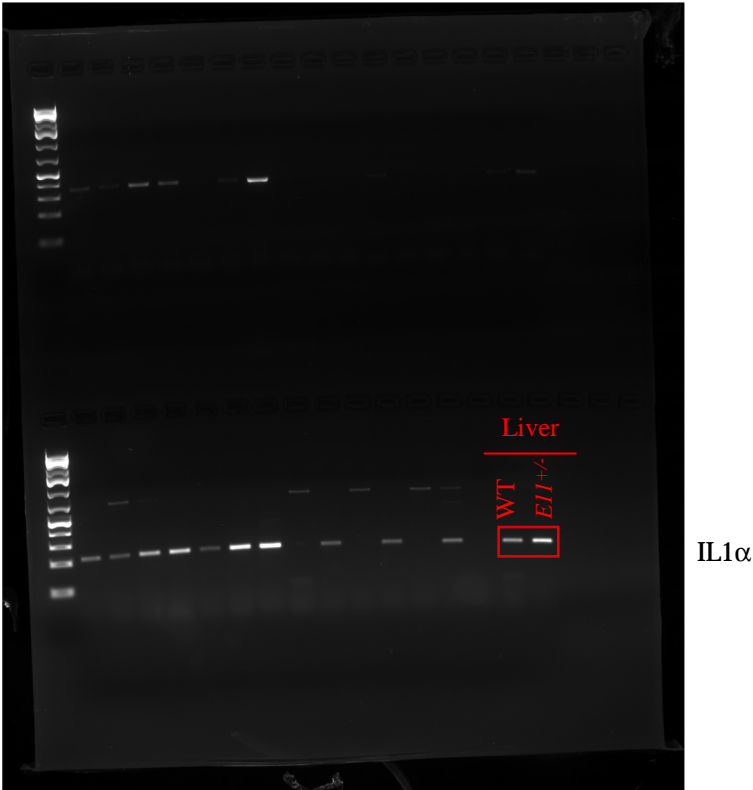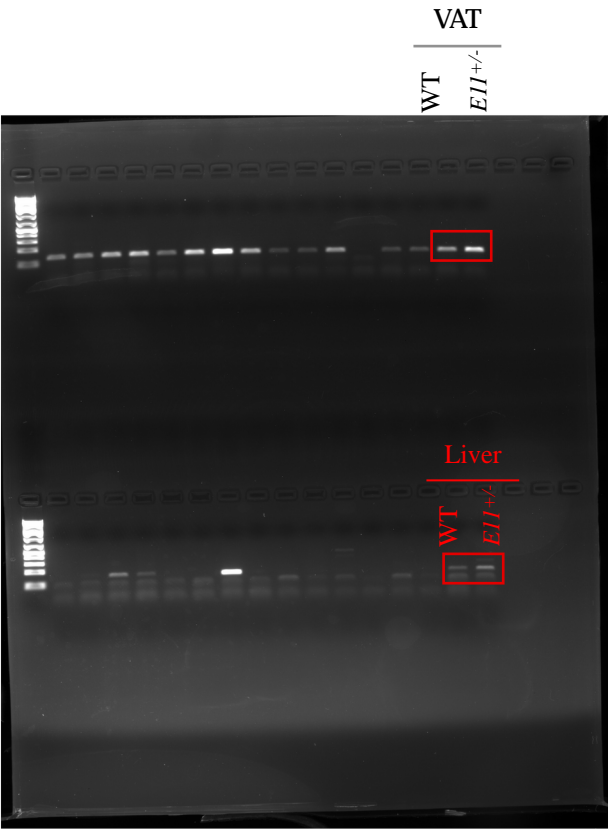

IL6

Leptin

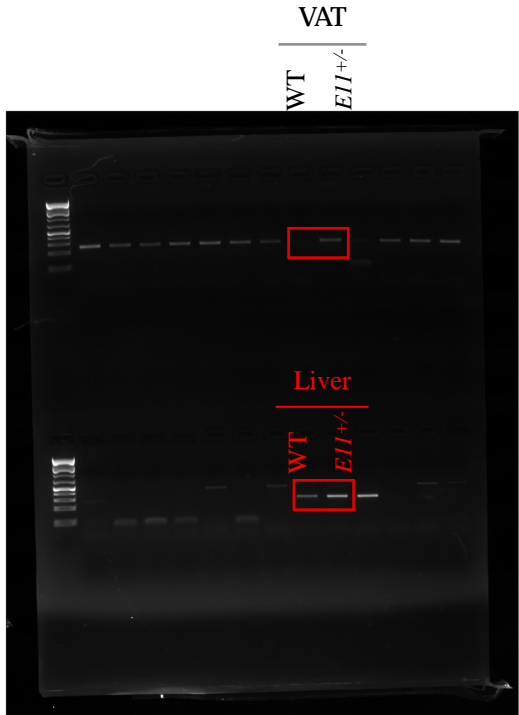

6A

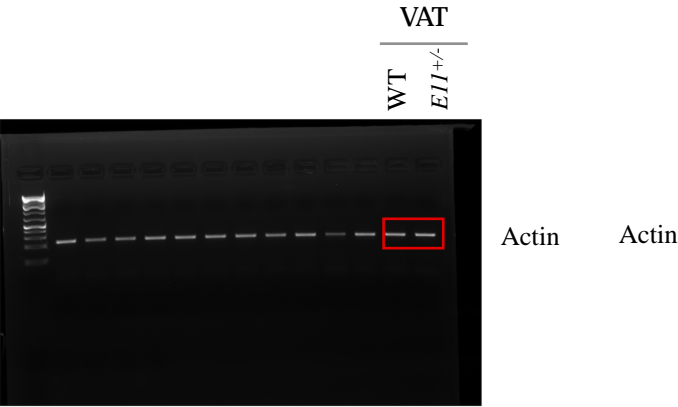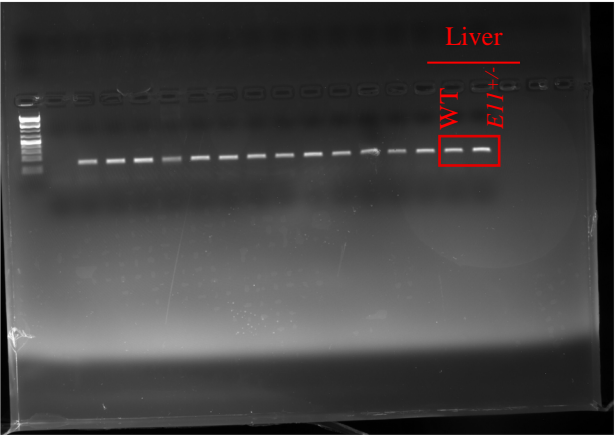

6F

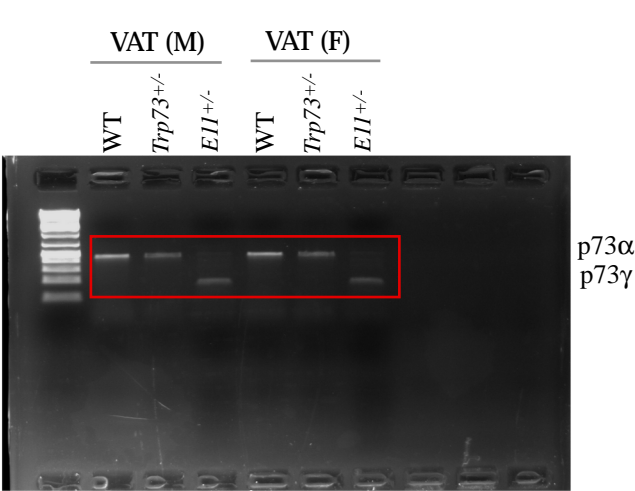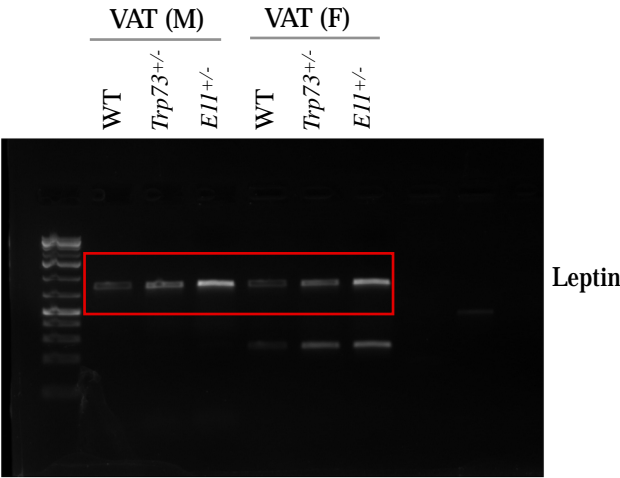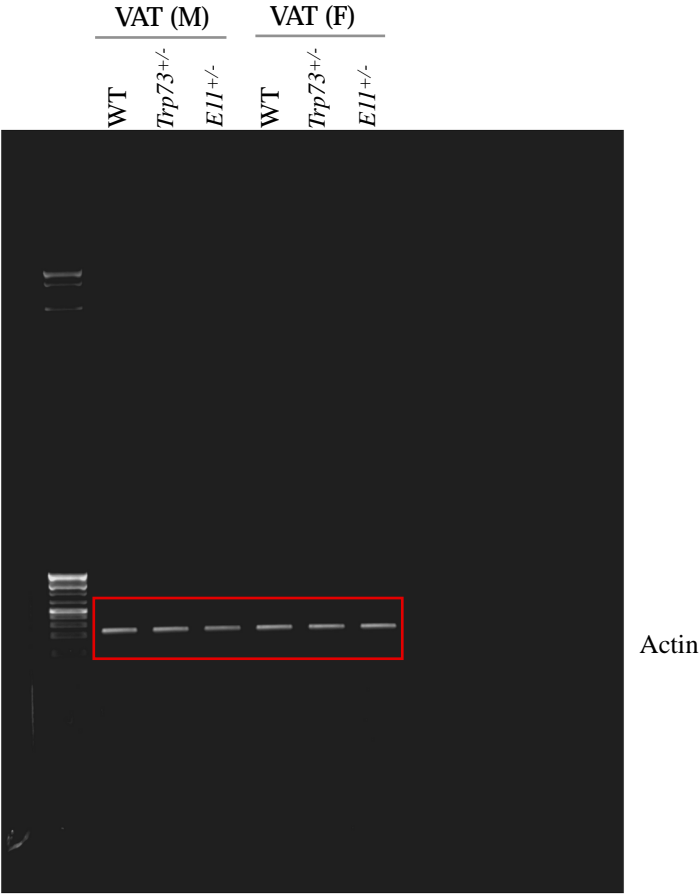

6G

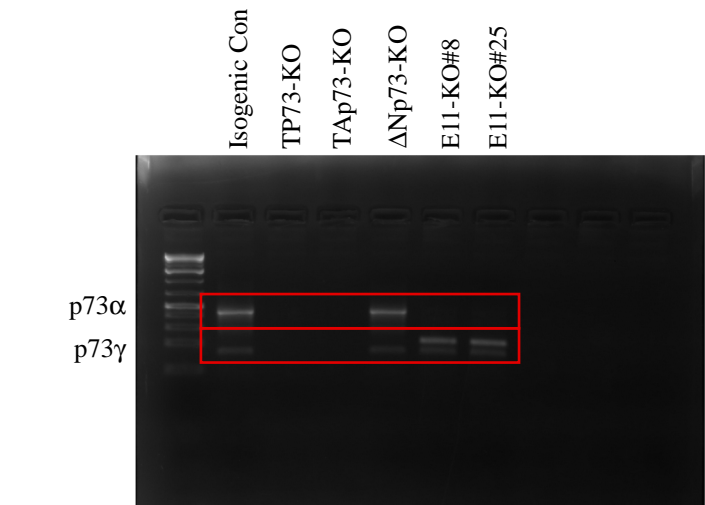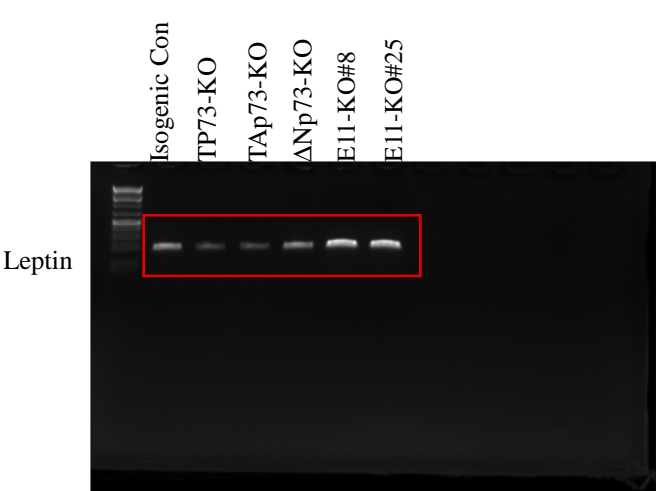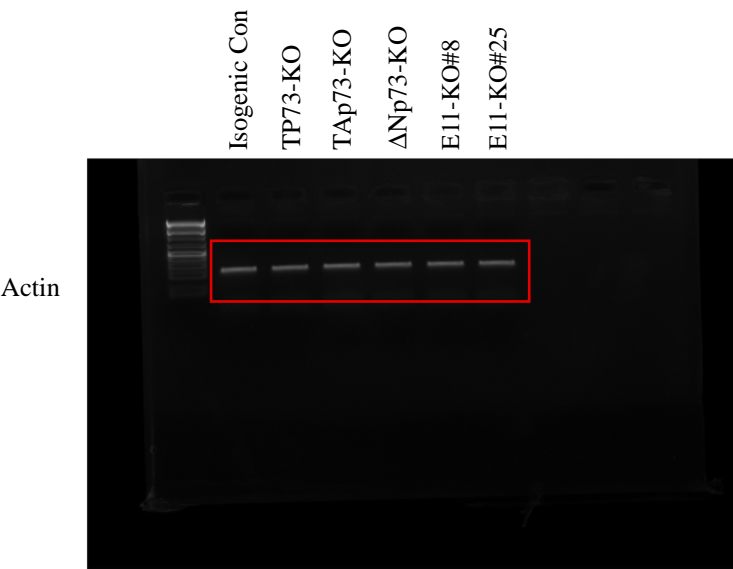

6H

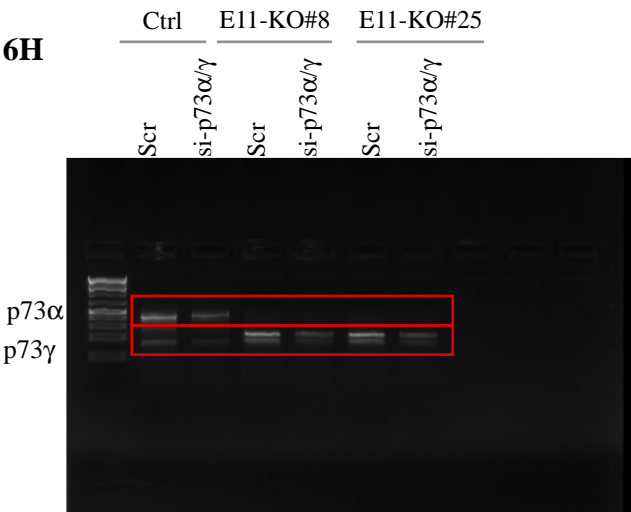

6H

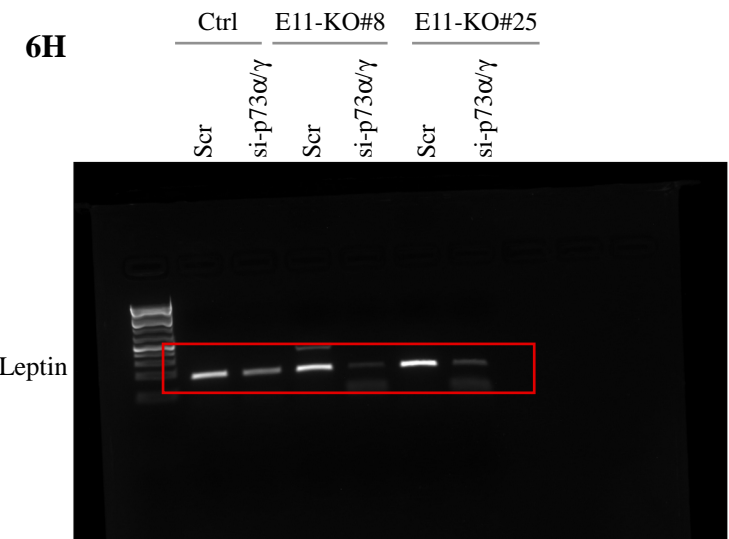

6H

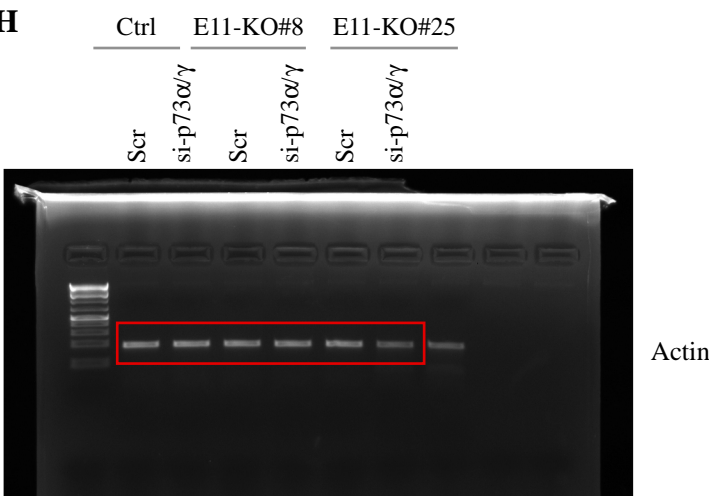

6J

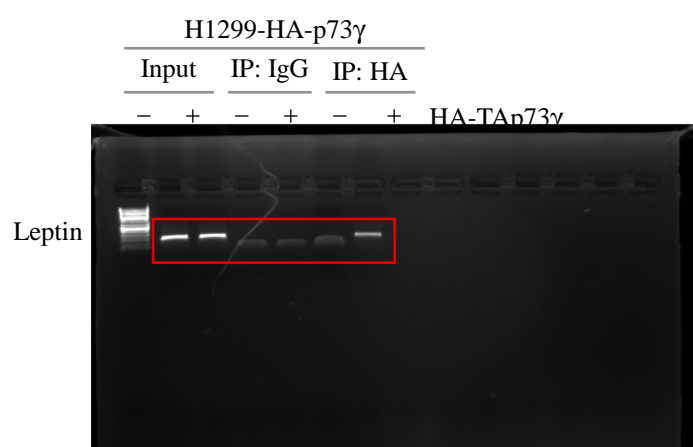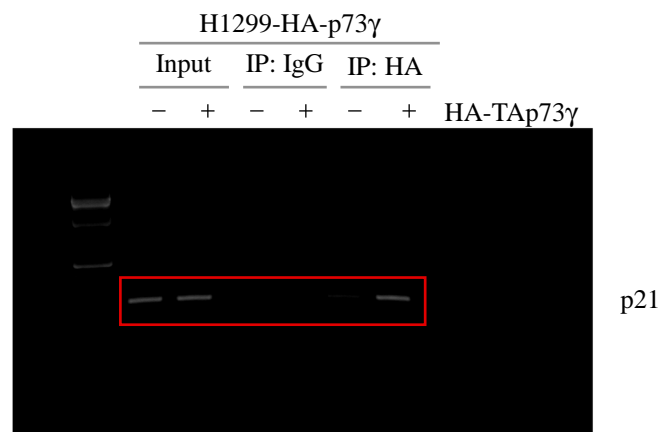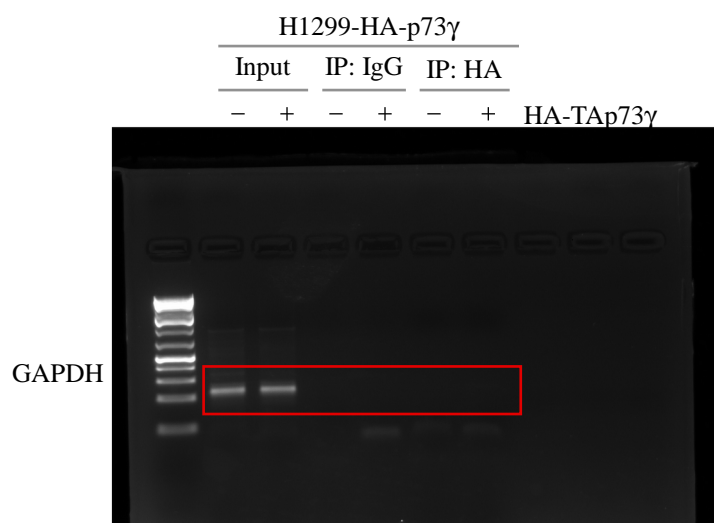

6K

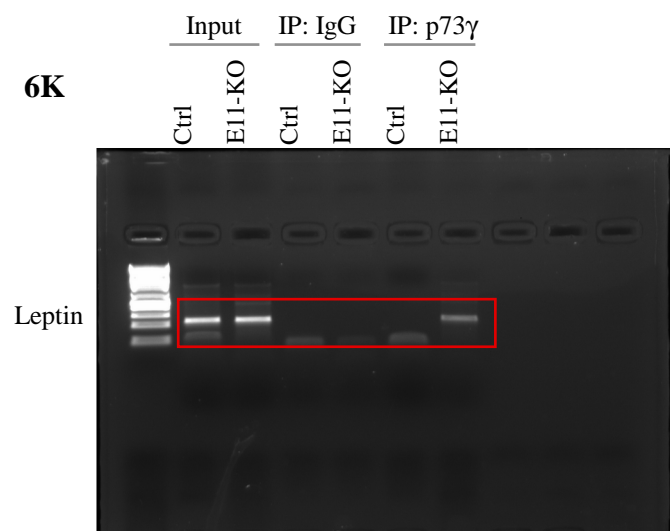

6K

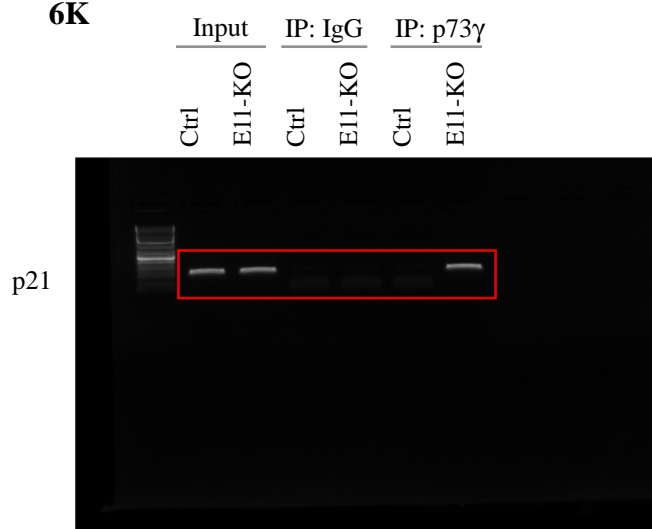

6K

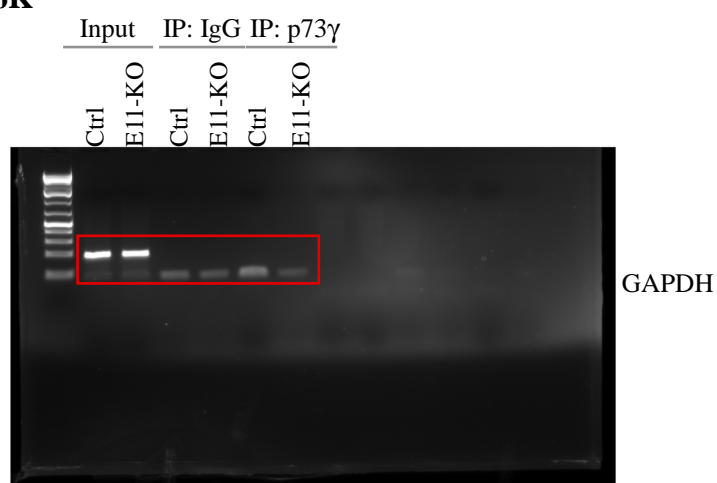

Supplement: Figure 6—source data 1. [file elife-82115-fig6-data1.pdf]

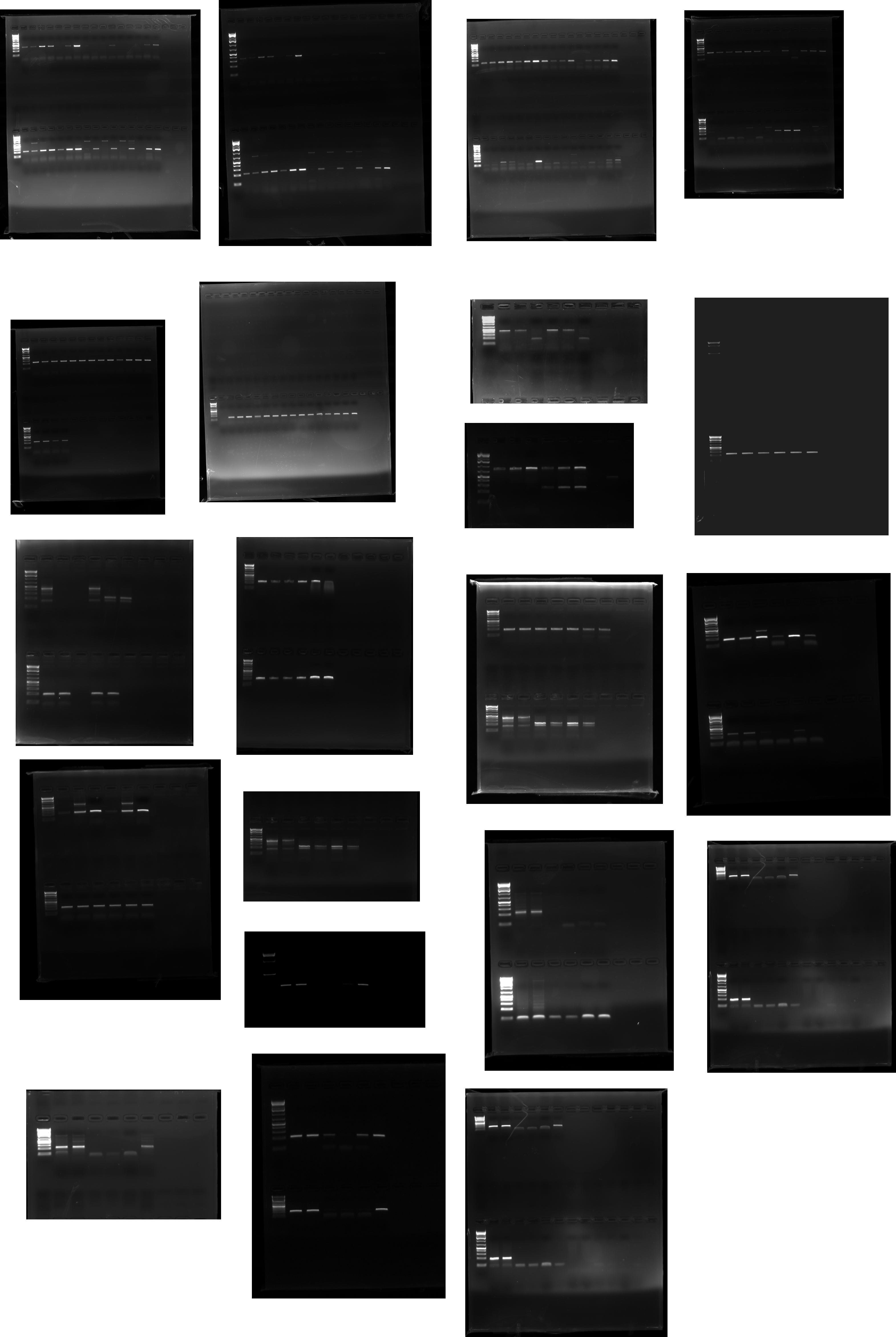

Supplement: Figure 6—source data 2. [file elife-82115-fig6-data2.tif]

6B

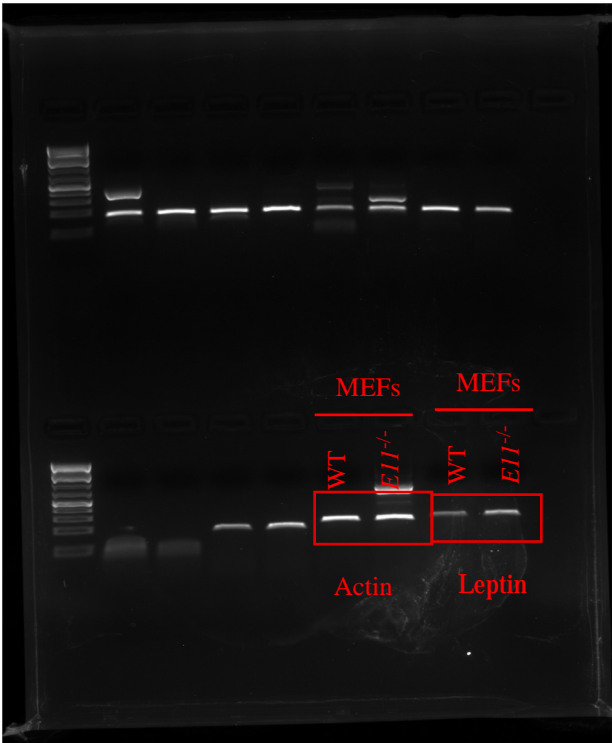

6D

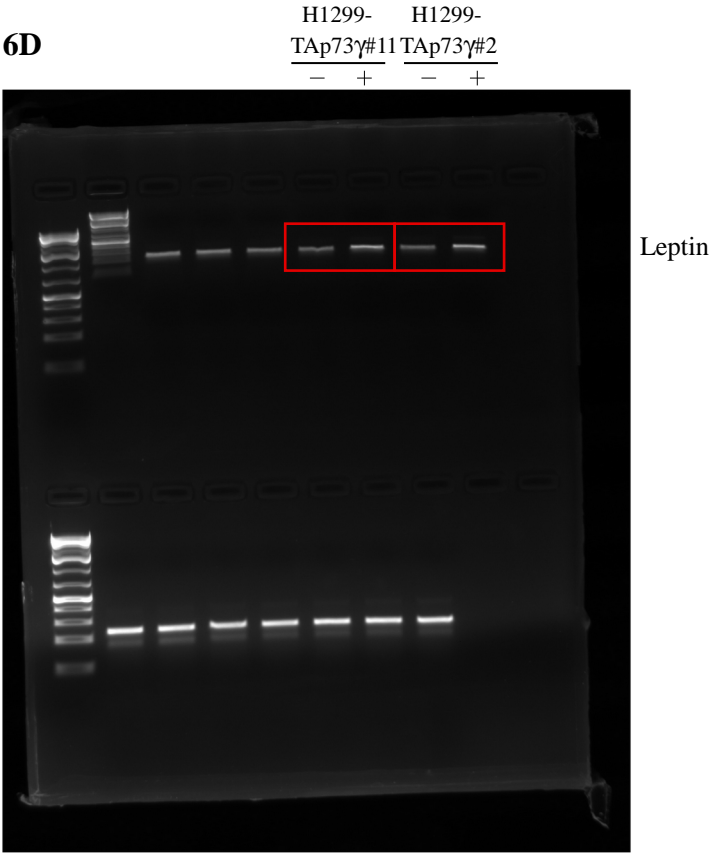

6D

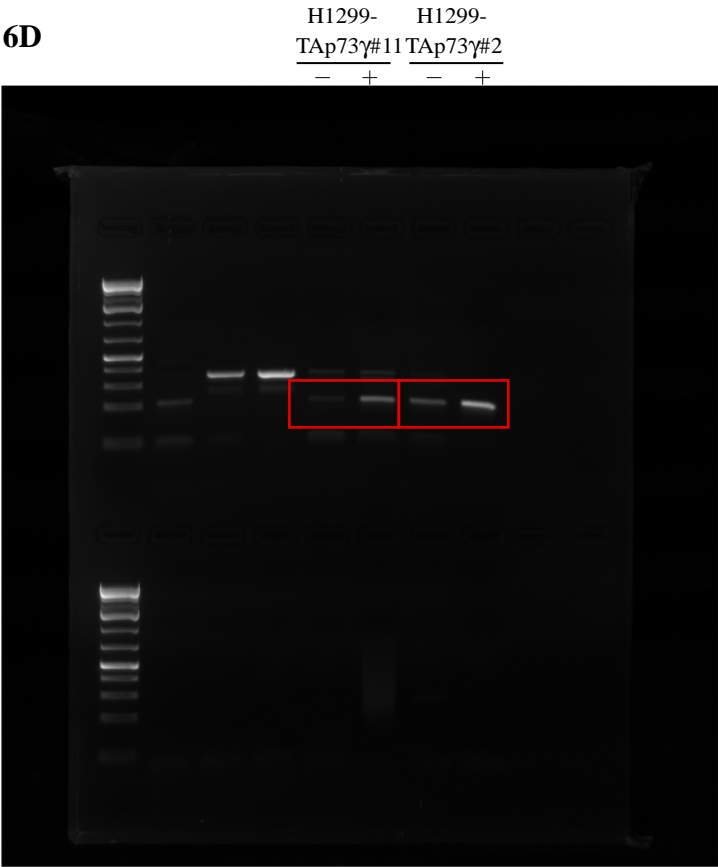

6D

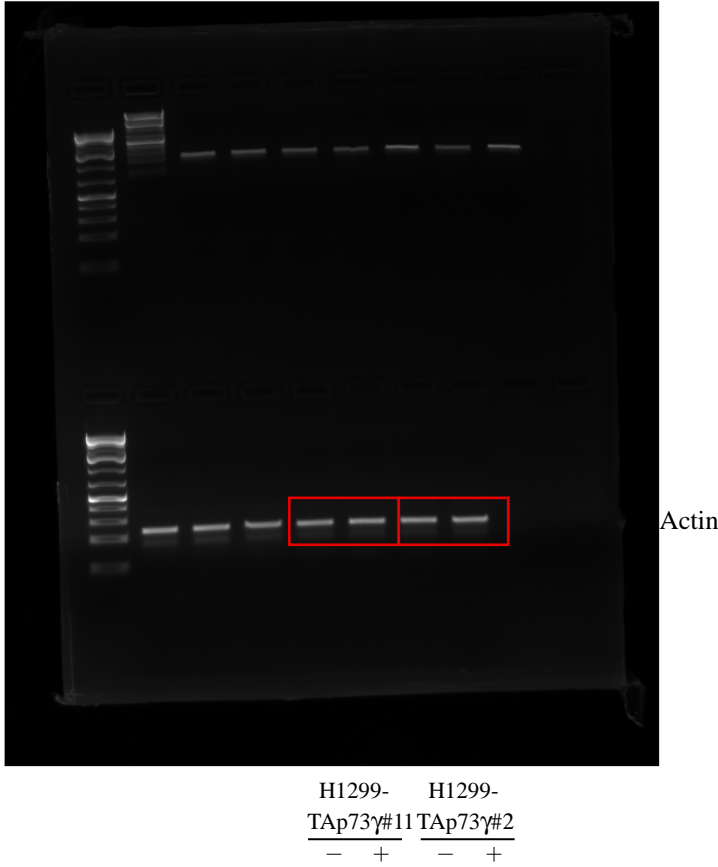

6E

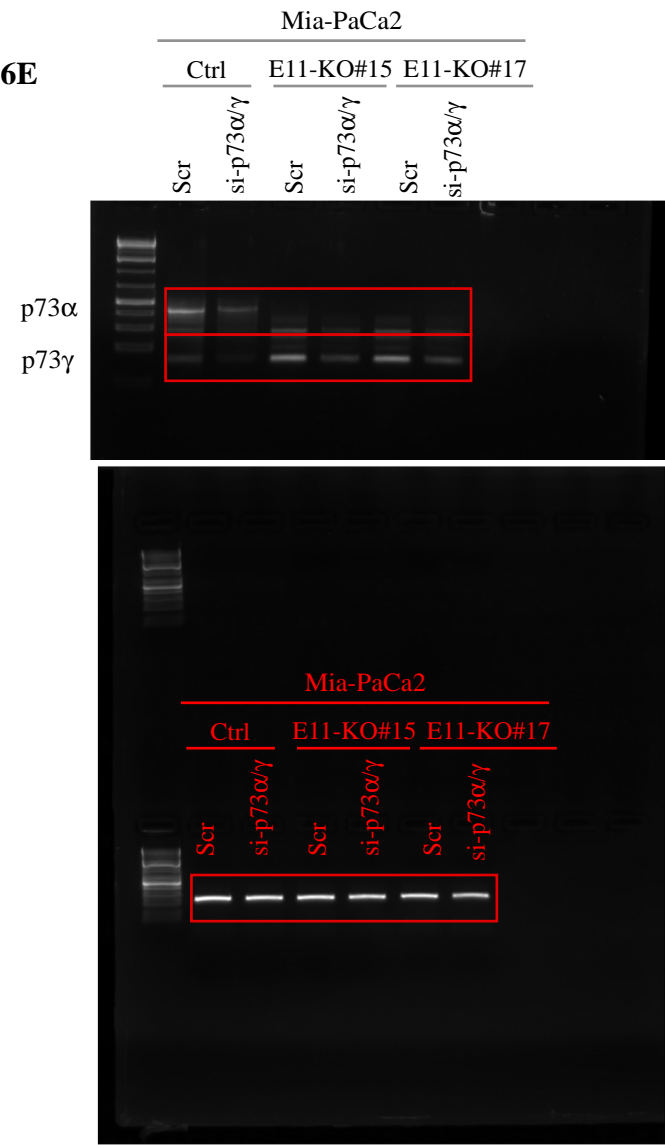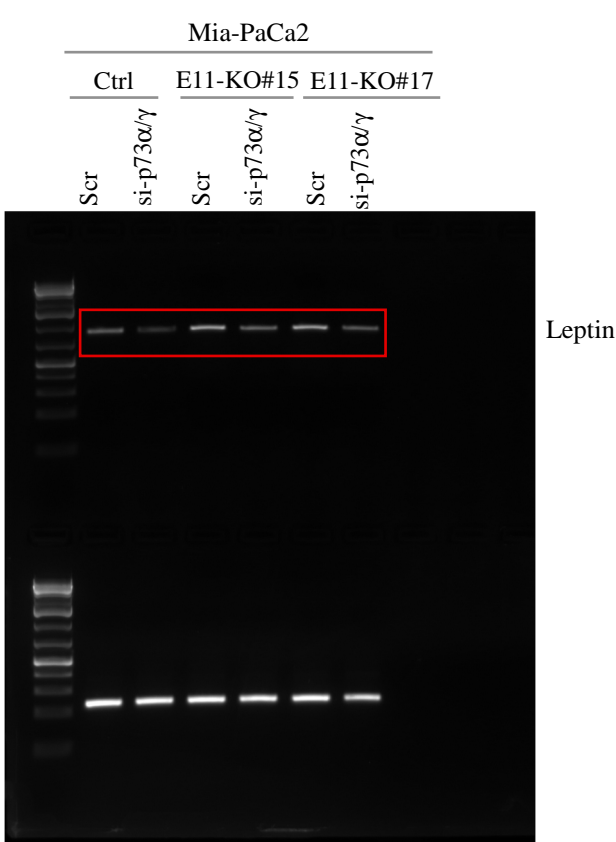

6F

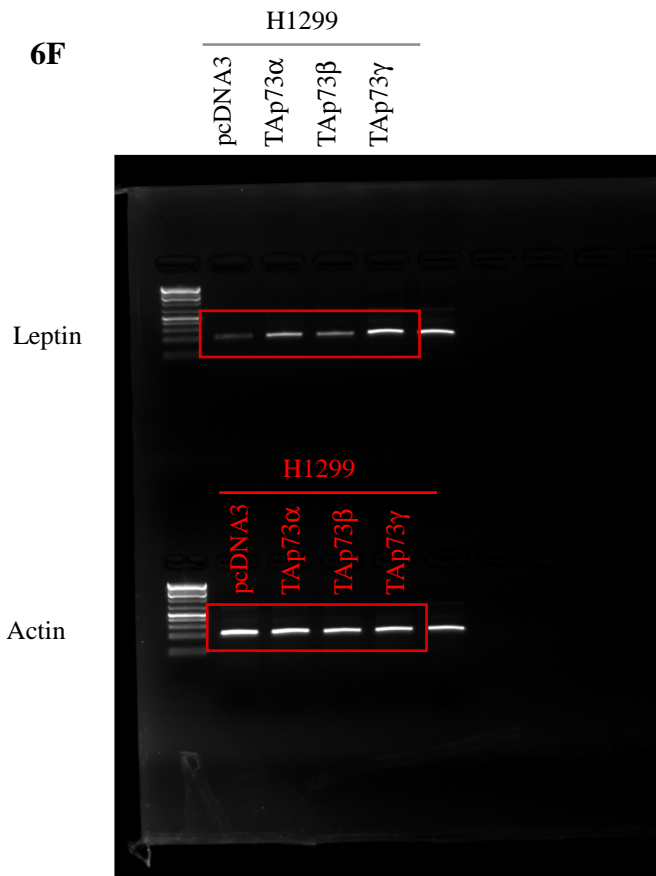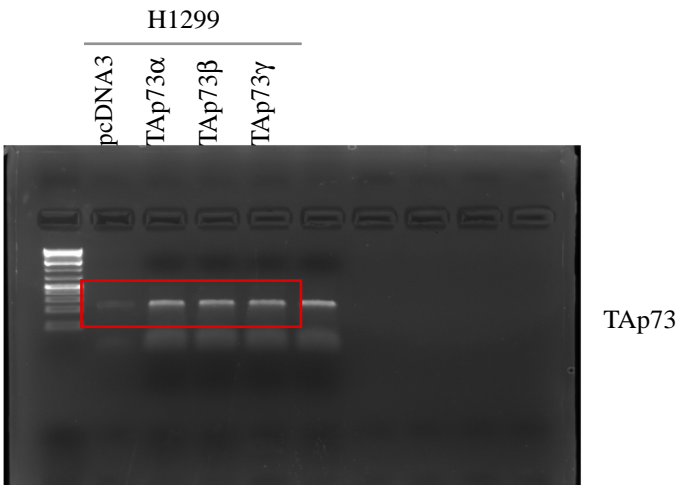

Supplement: Figure 6—figure supplement 1—source data 1. [file elife-82115-fig6-figsupp1-data1.pdf]

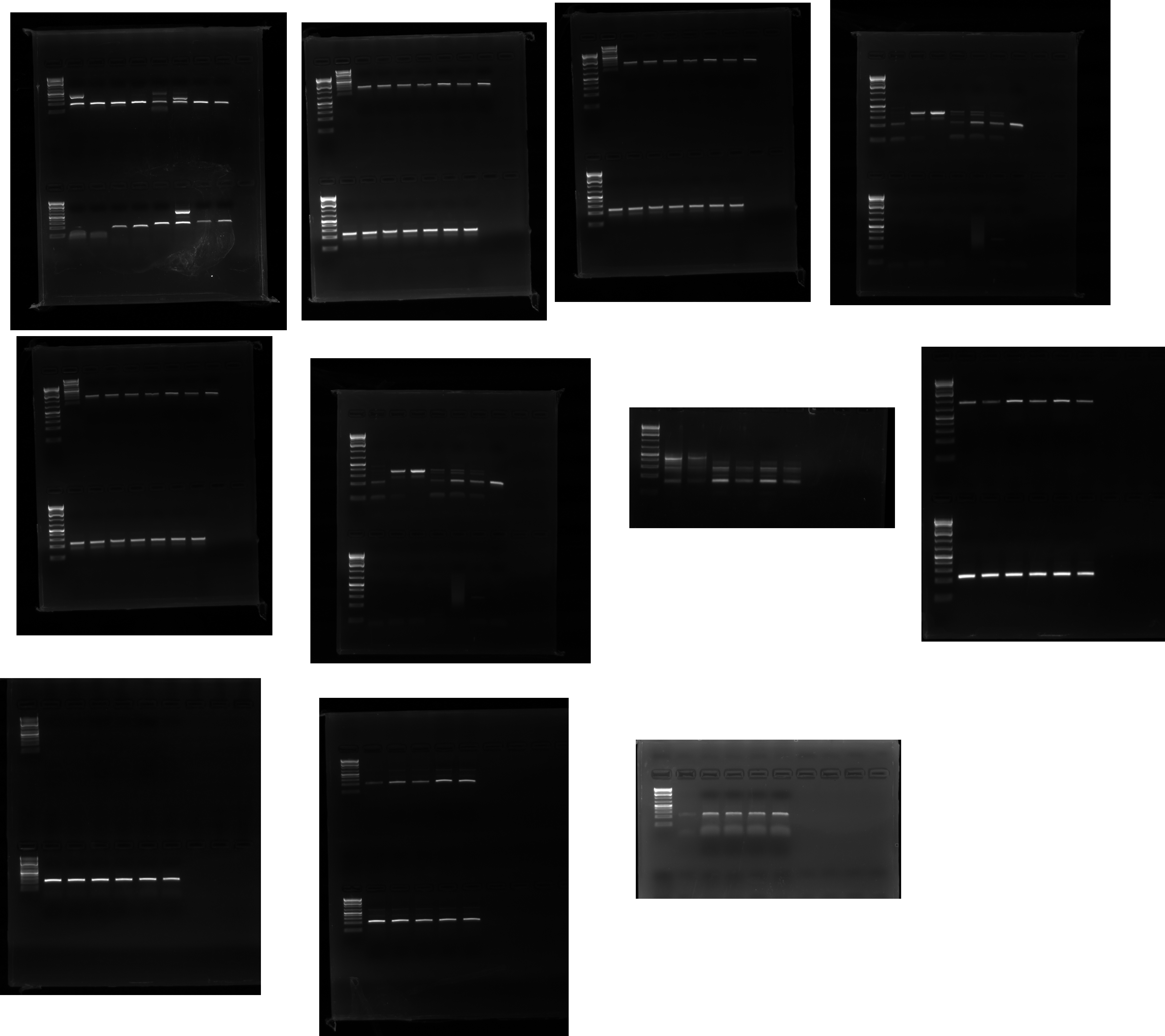

Supplement: Figure 6—figure supplement 1—source data 2. [file elife-82115-fig6-figsupp1-data2.tif]

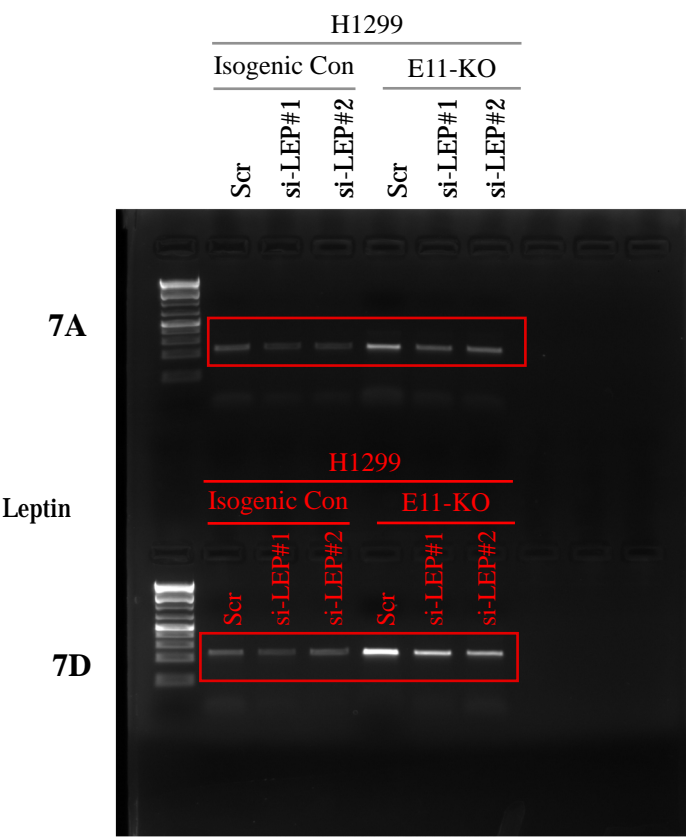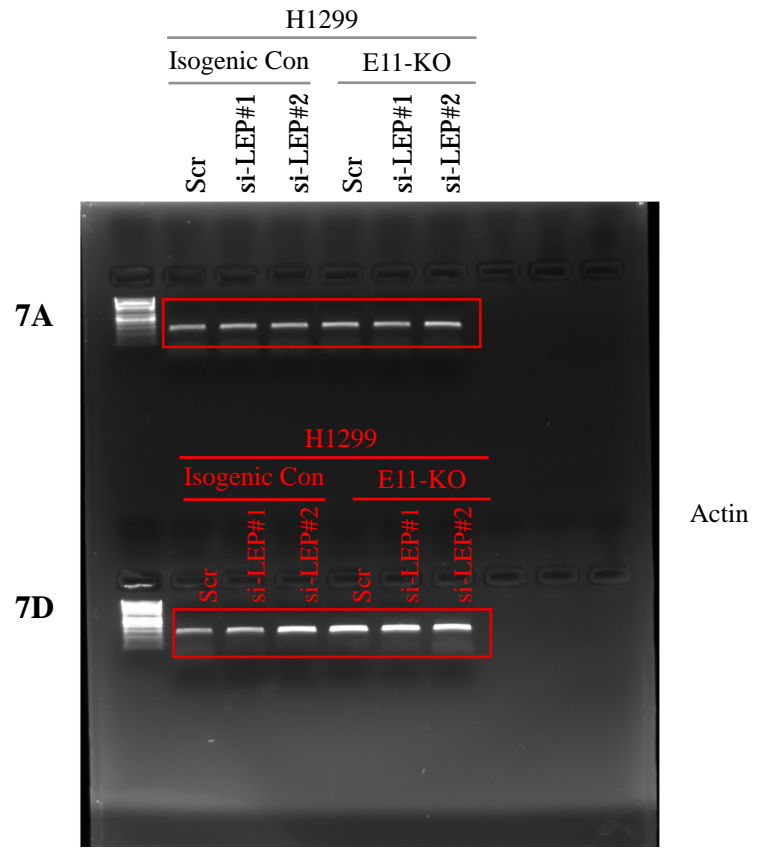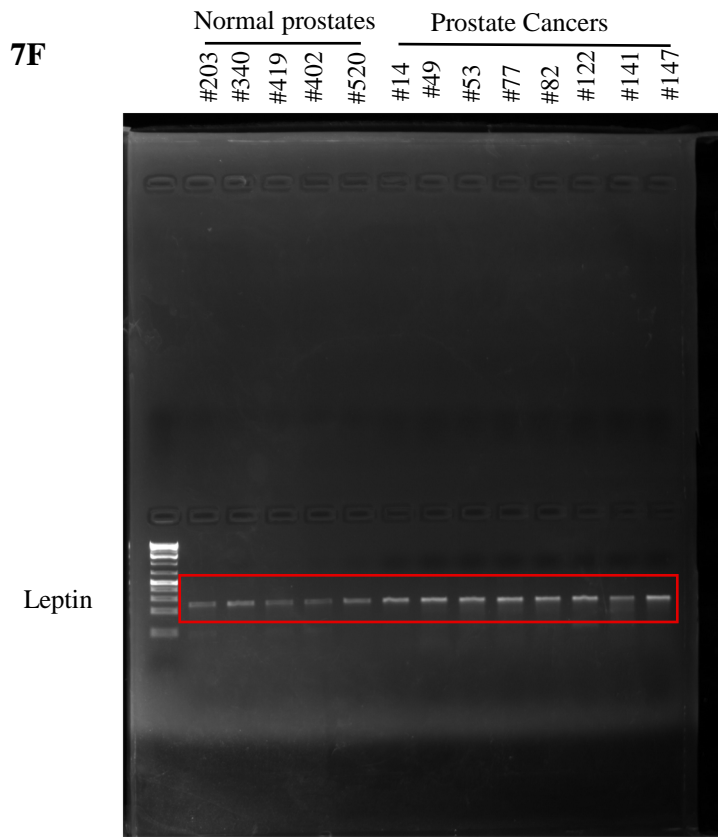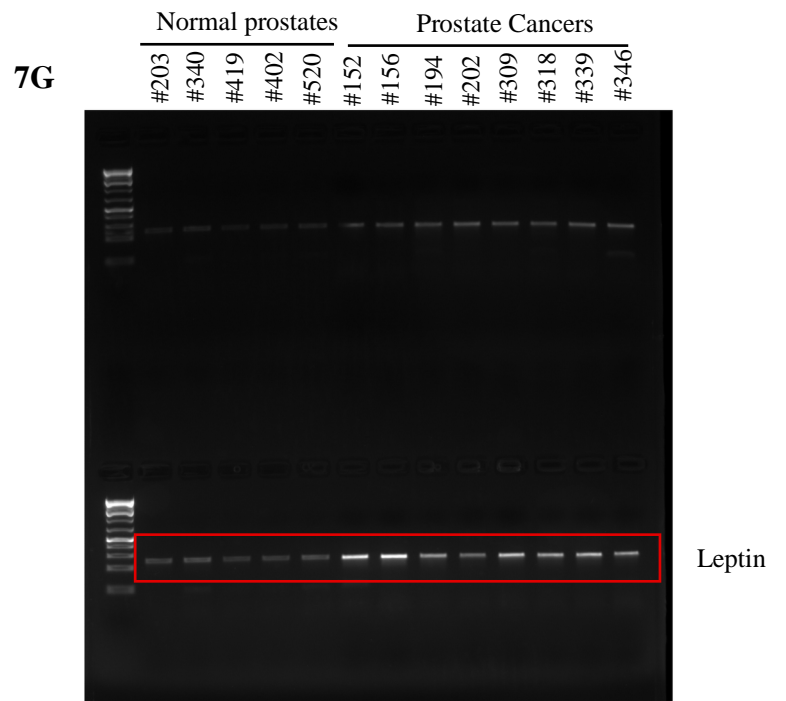

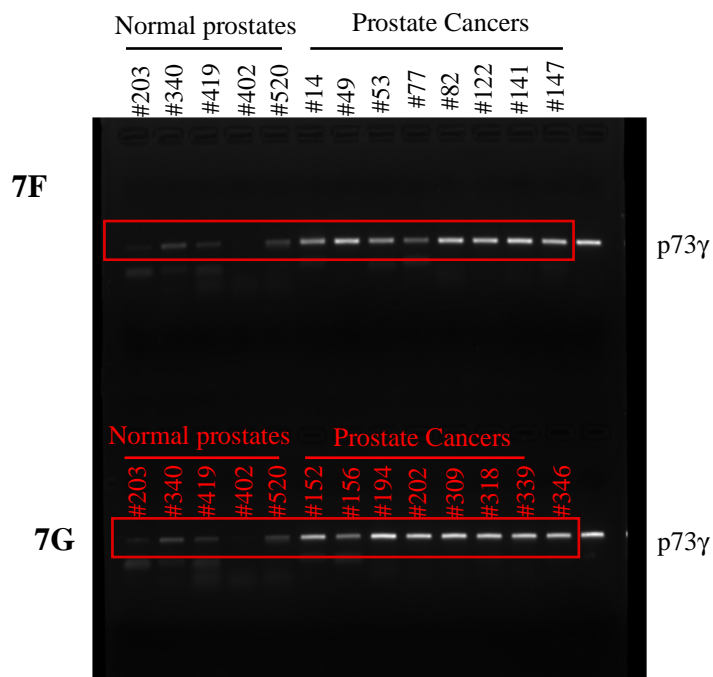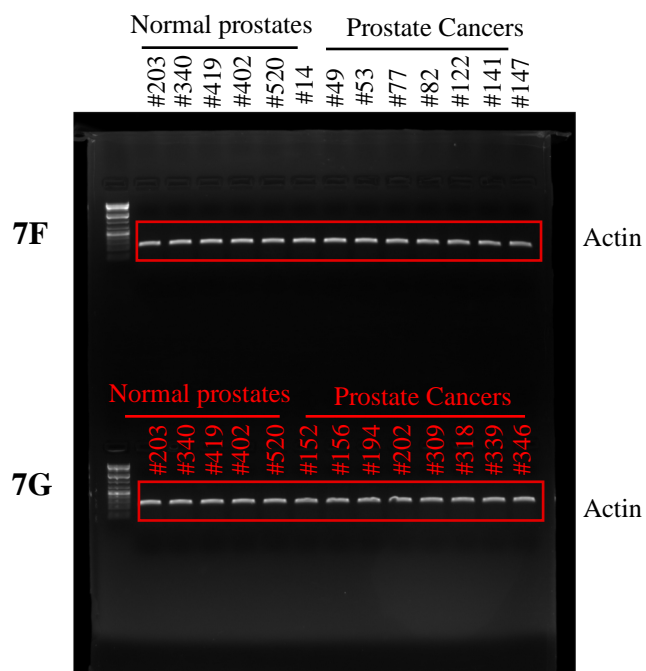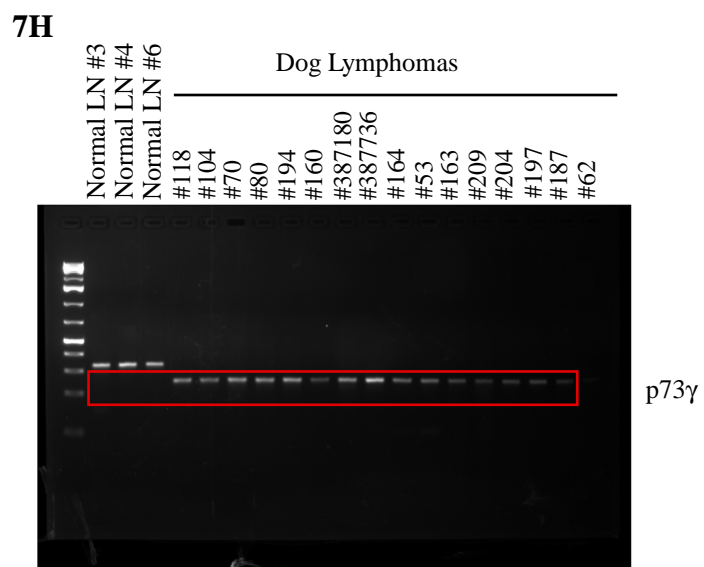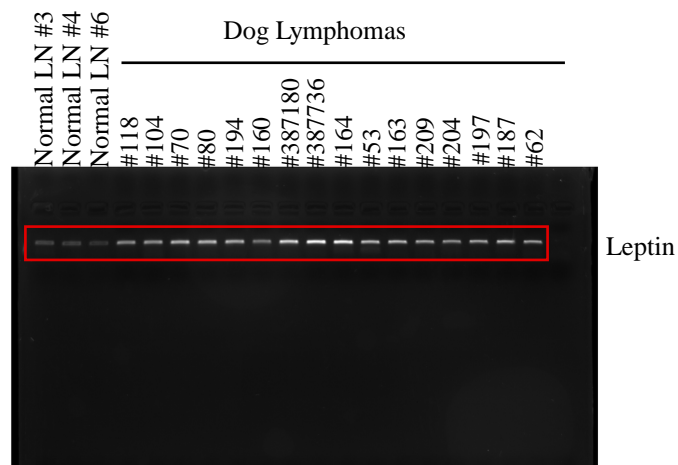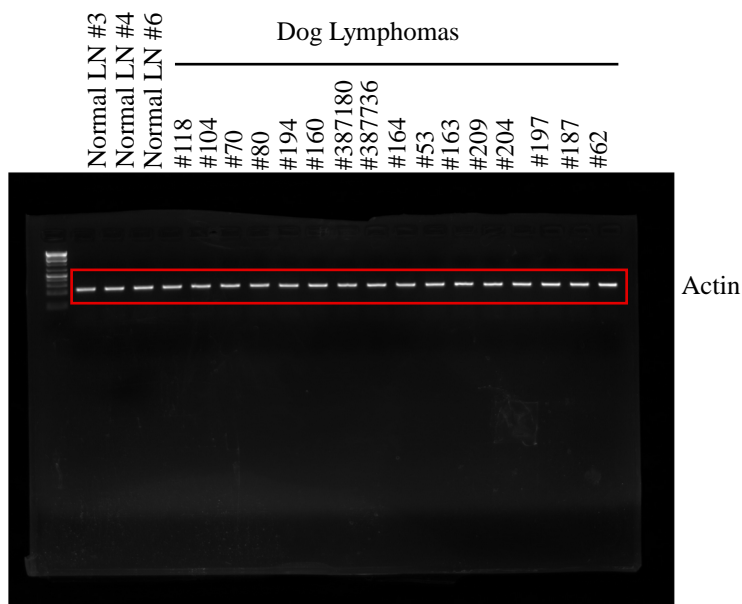

Supplement: Figure 7—source data 1. [file elife-82115-fig7-data1.pdf]

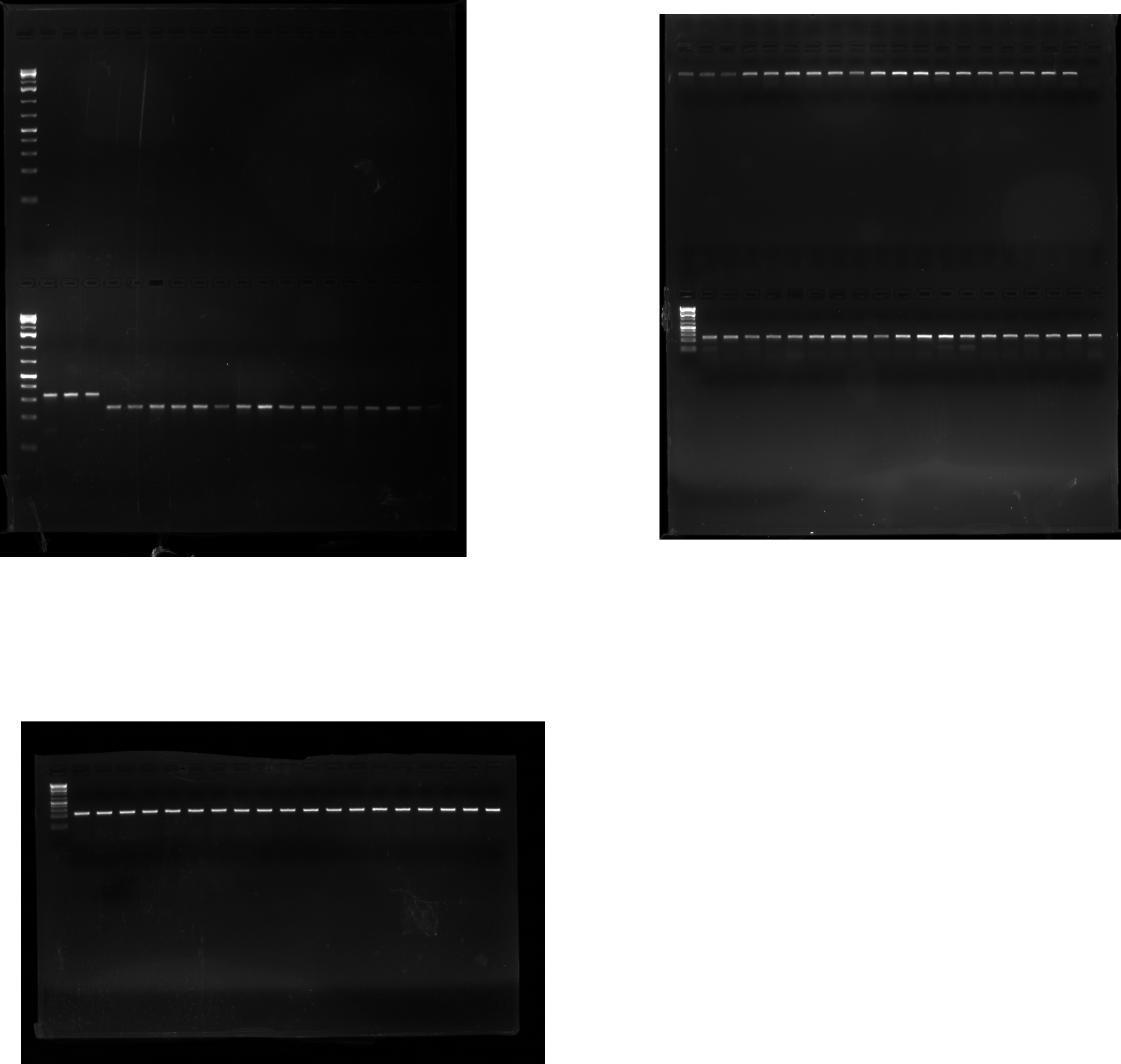

Supplement: Figure 7—source data 2. [file elife-82115-fig7-data2.zip › Figure 7- Source data 2a.tif]

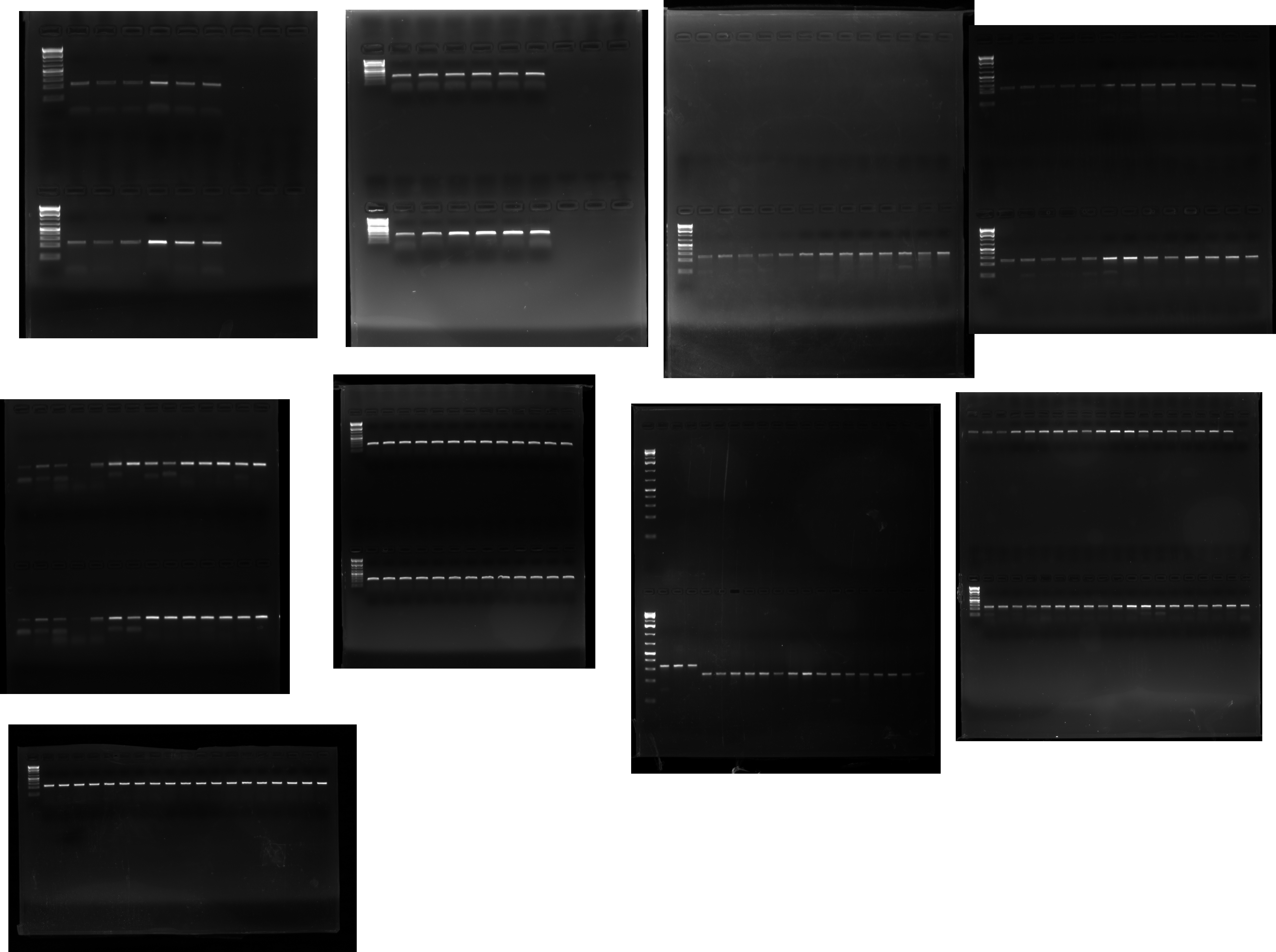

Supplement: Figure 7—source data 2. [file elife-82115-fig7-data2.zip › Figure 7- Source data 2b.tif]

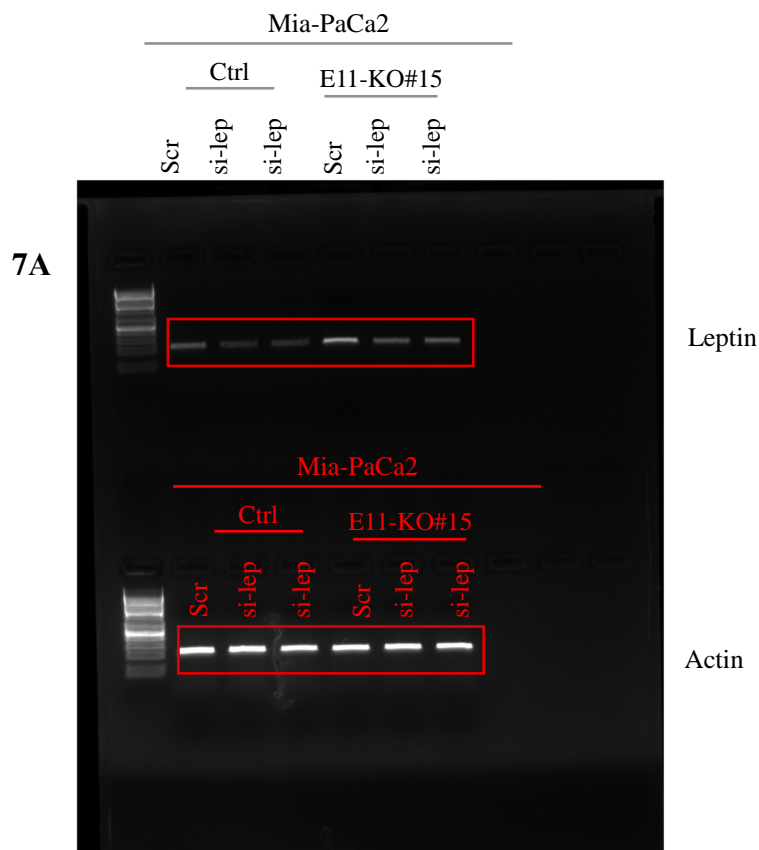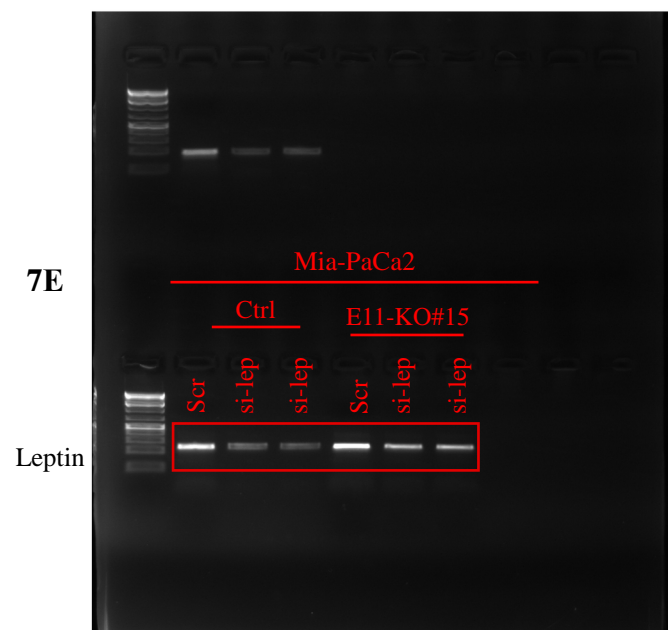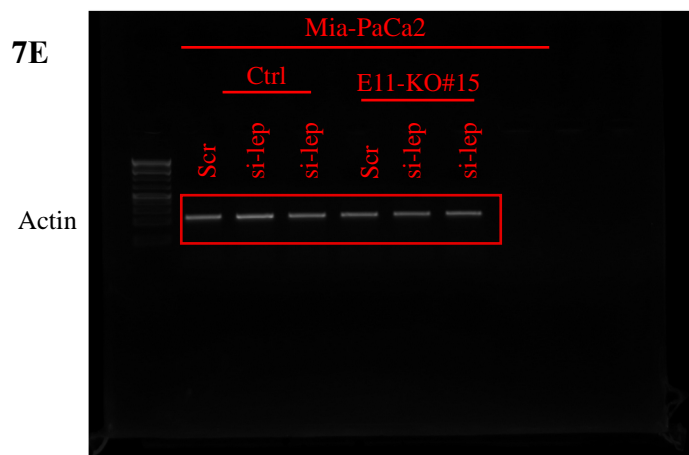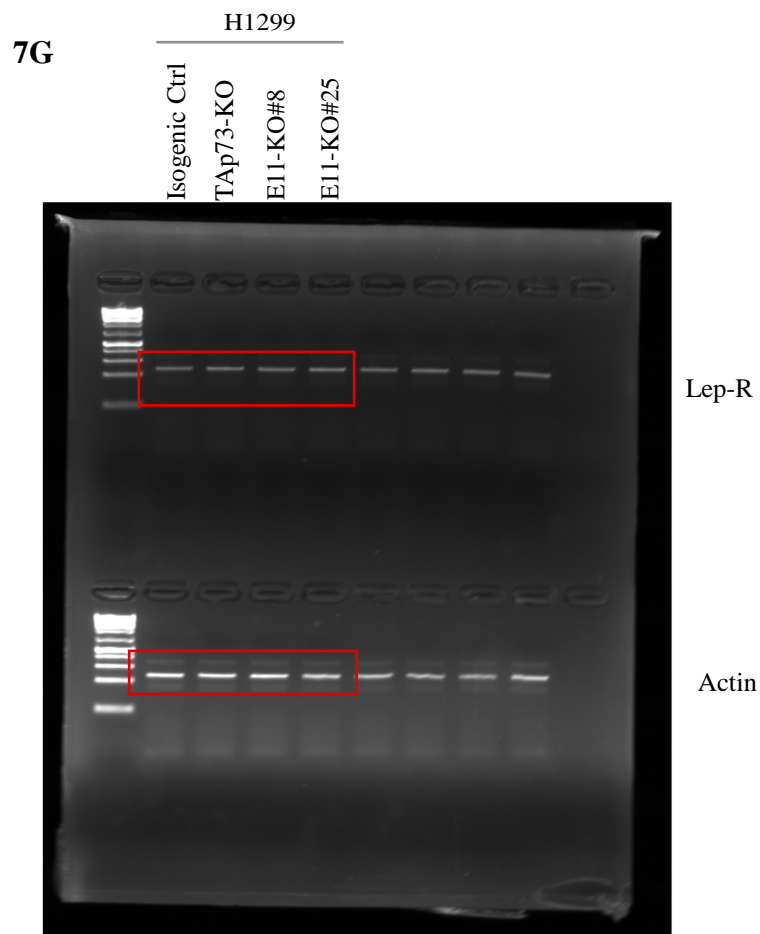

Supplement: Figure 7—figure supplement 1—source data 1. [file elife-82115-fig7-figsupp1-data1.pdf]

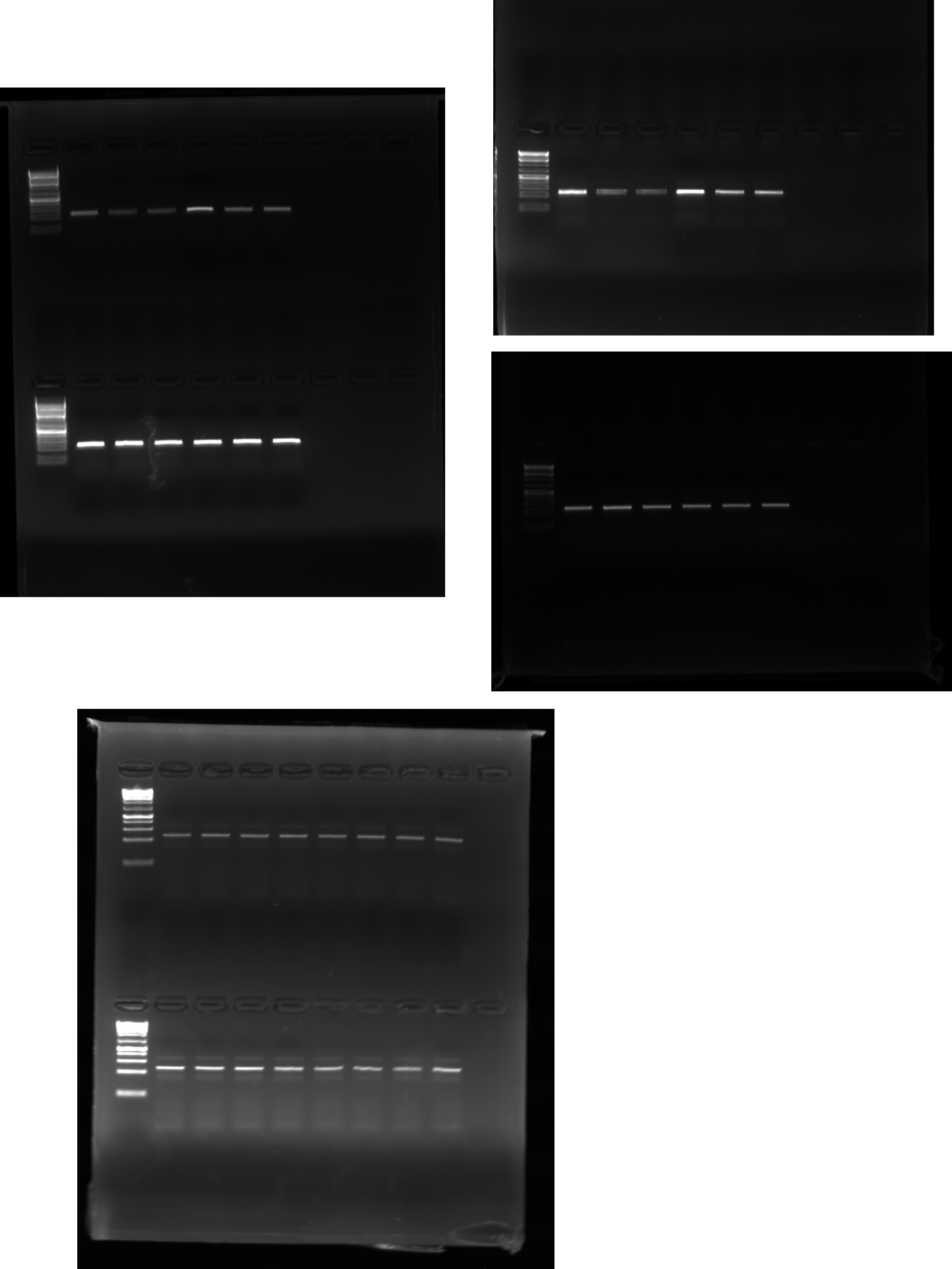

Supplement: Figure 7—figure supplement 1—source data 2. [file elife-82115-fig7-figsupp1-data2.tif]

8D

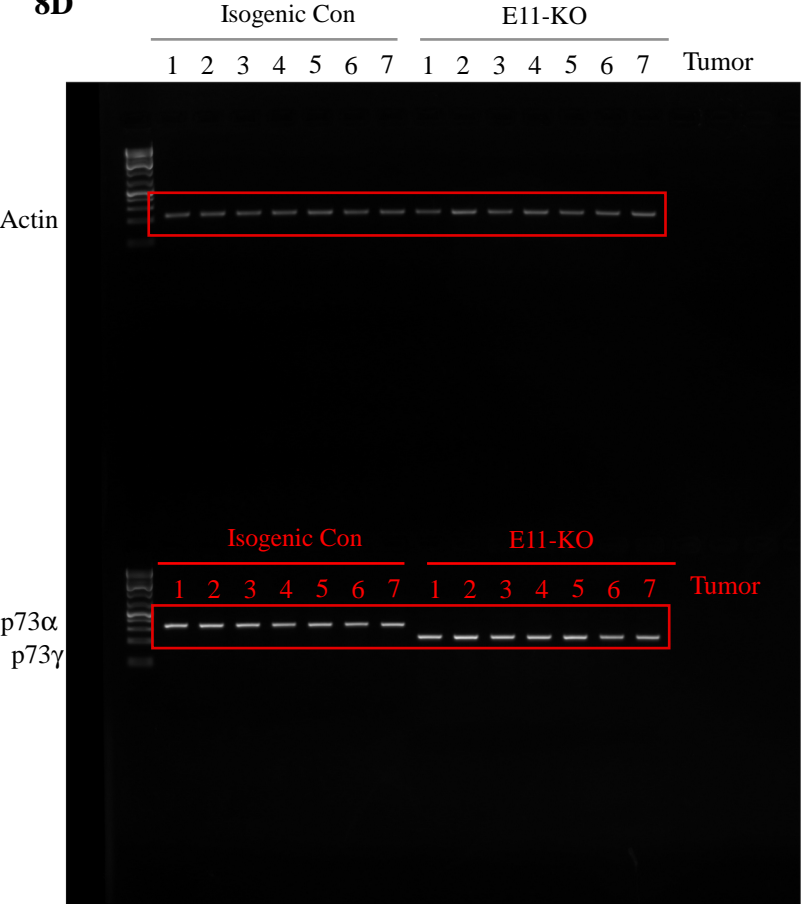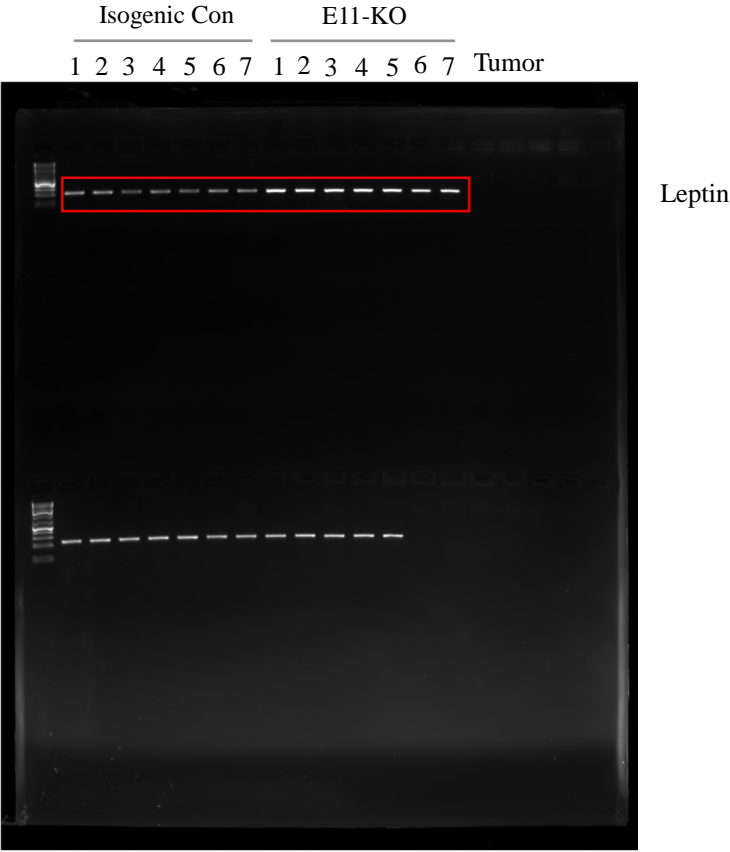

8I

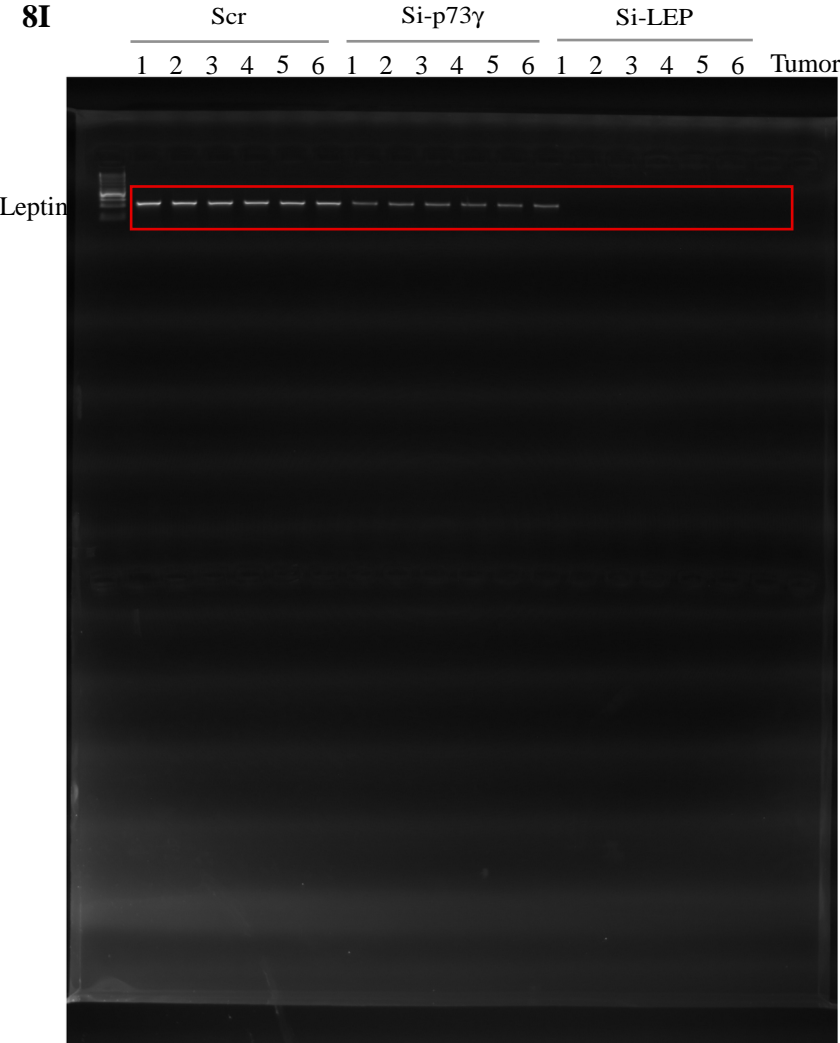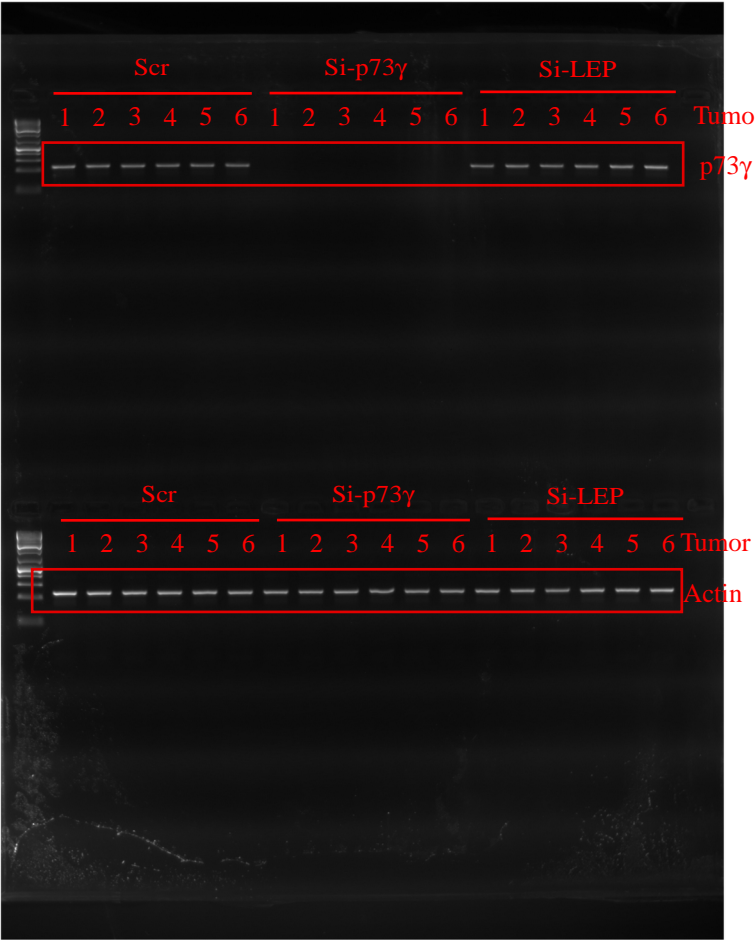

Supplement: Figure 8—source data 1. [file elife-82115-fig8-data1.pdf]

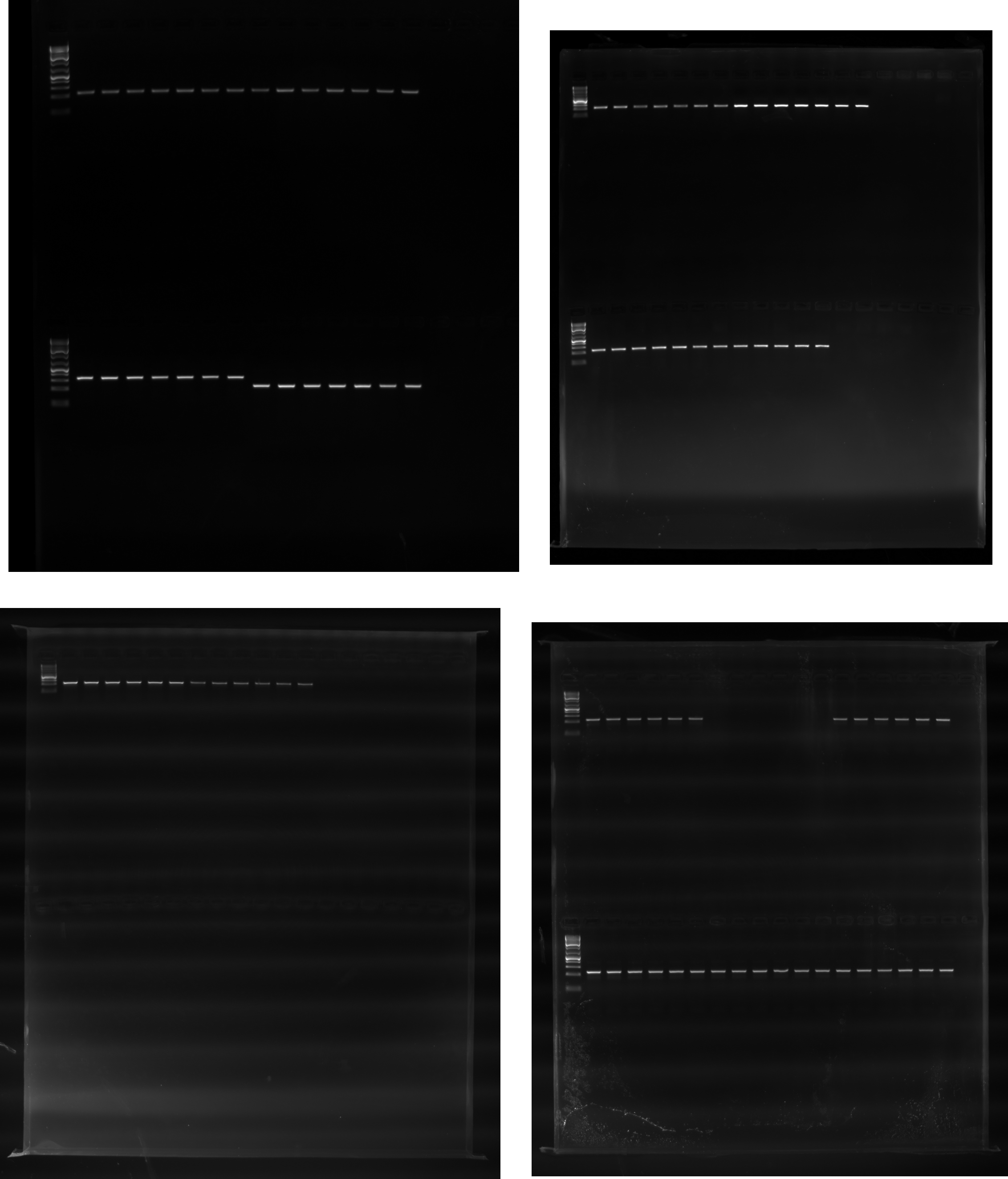

Supplement: Figure 8—source data 2. [file elife-82115-fig8-data2.tif]

8A

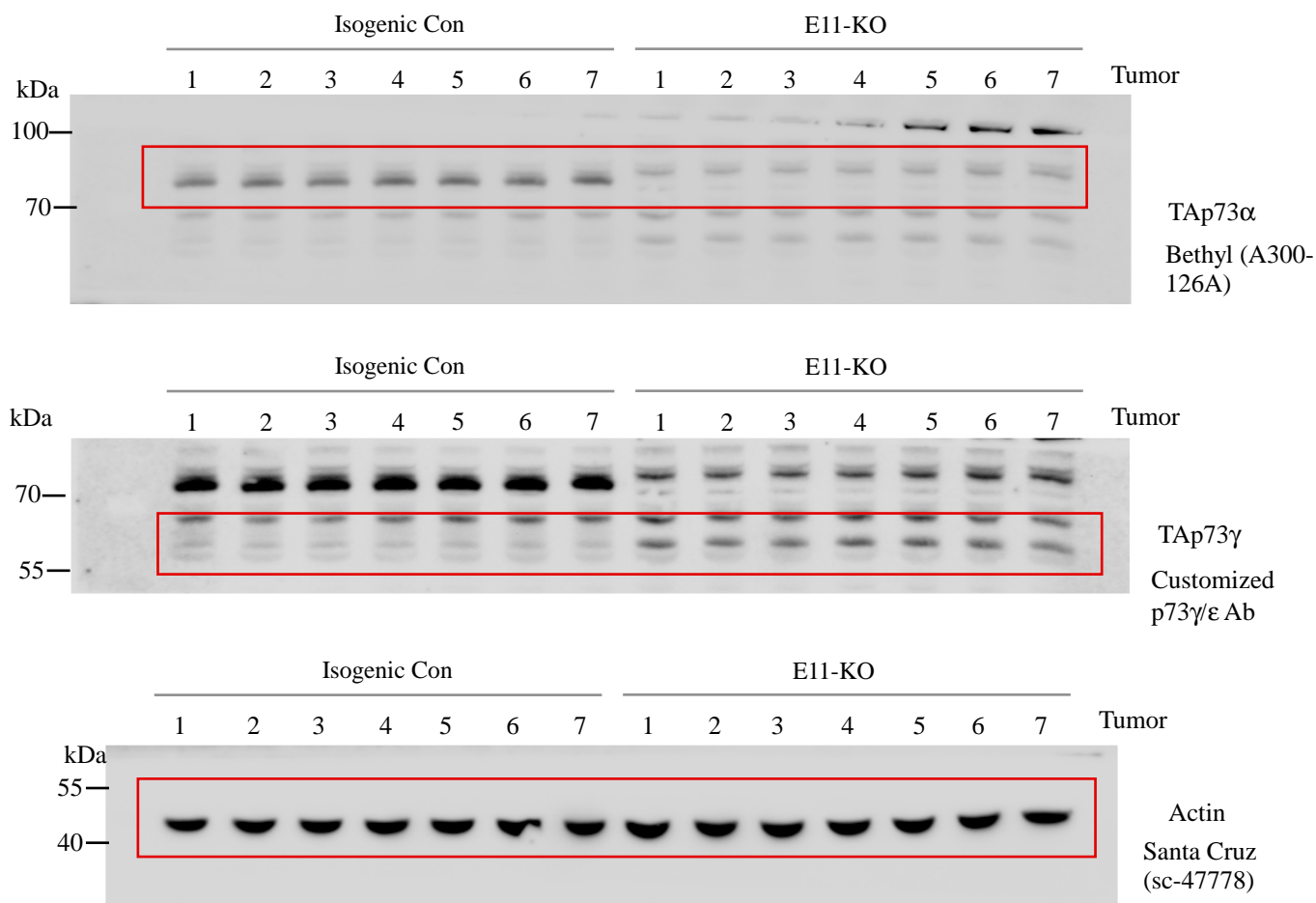

8B

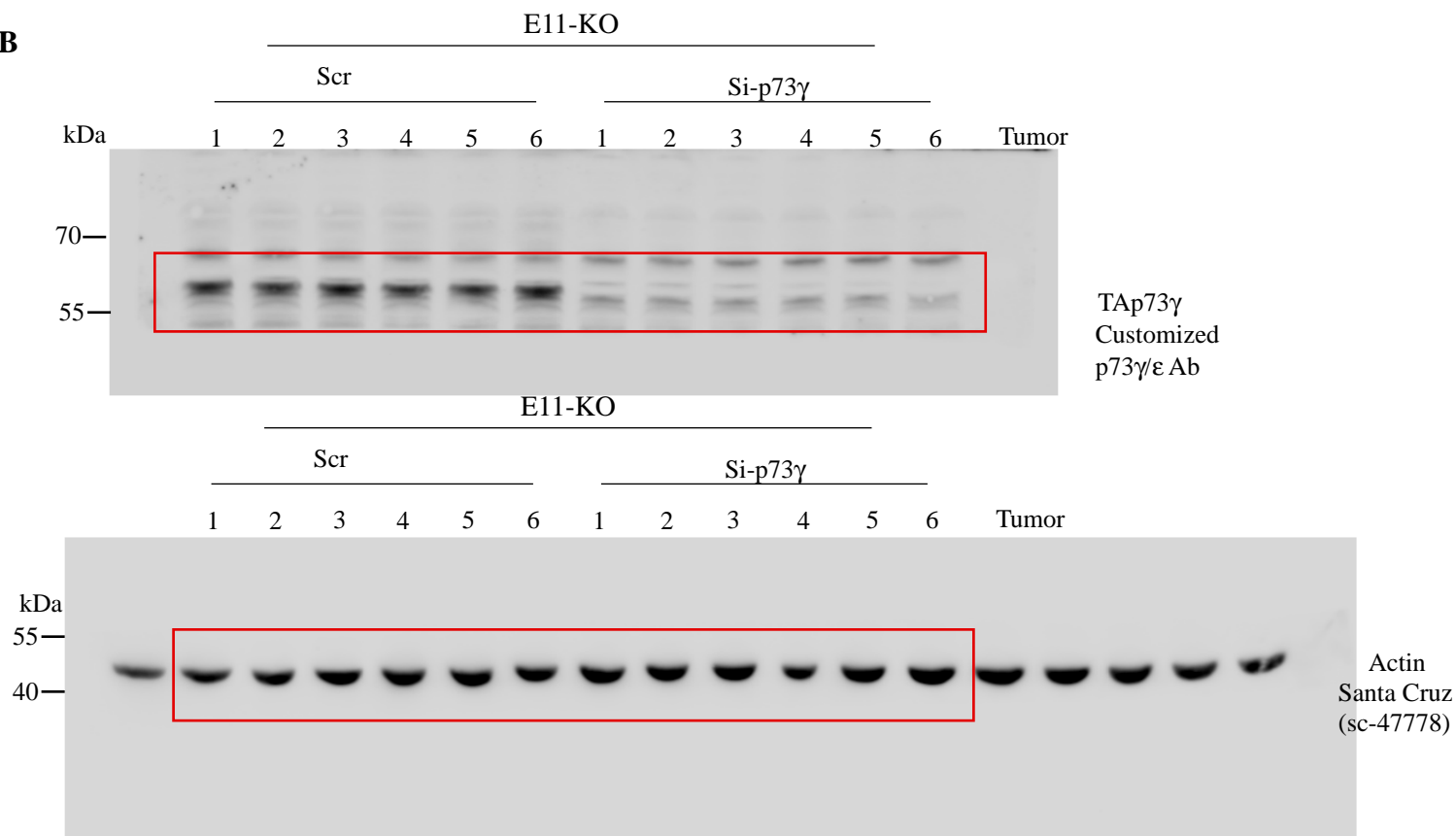

8C

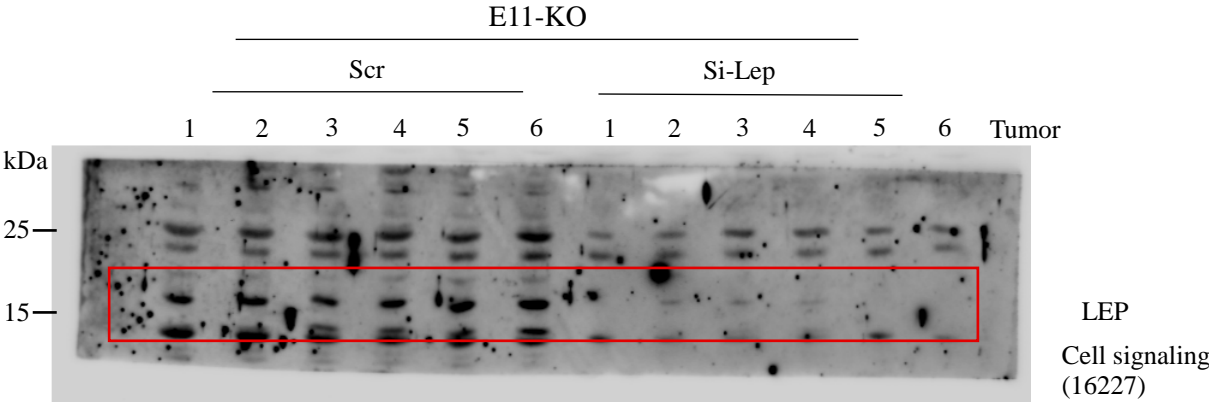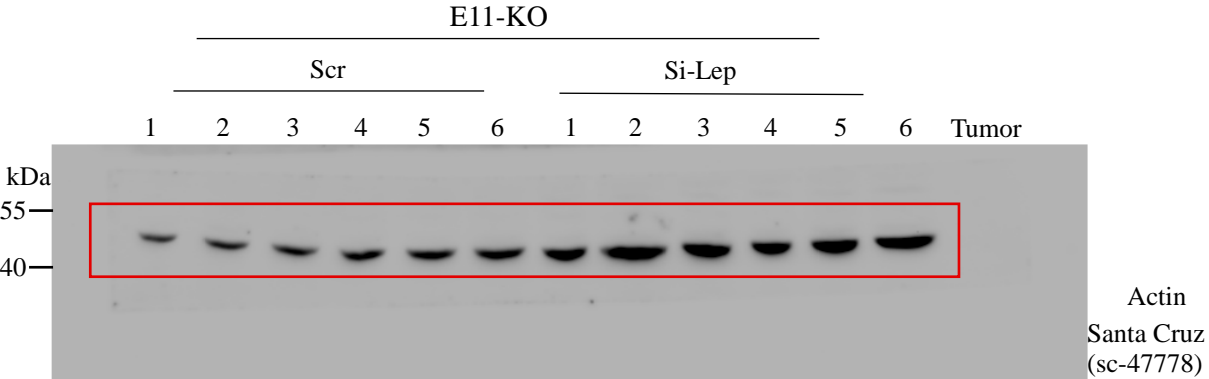

Supplement: Figure 8—figure supplement 1—source data 1. [file elife-82115-fig8-figsupp1-data1.pdf]

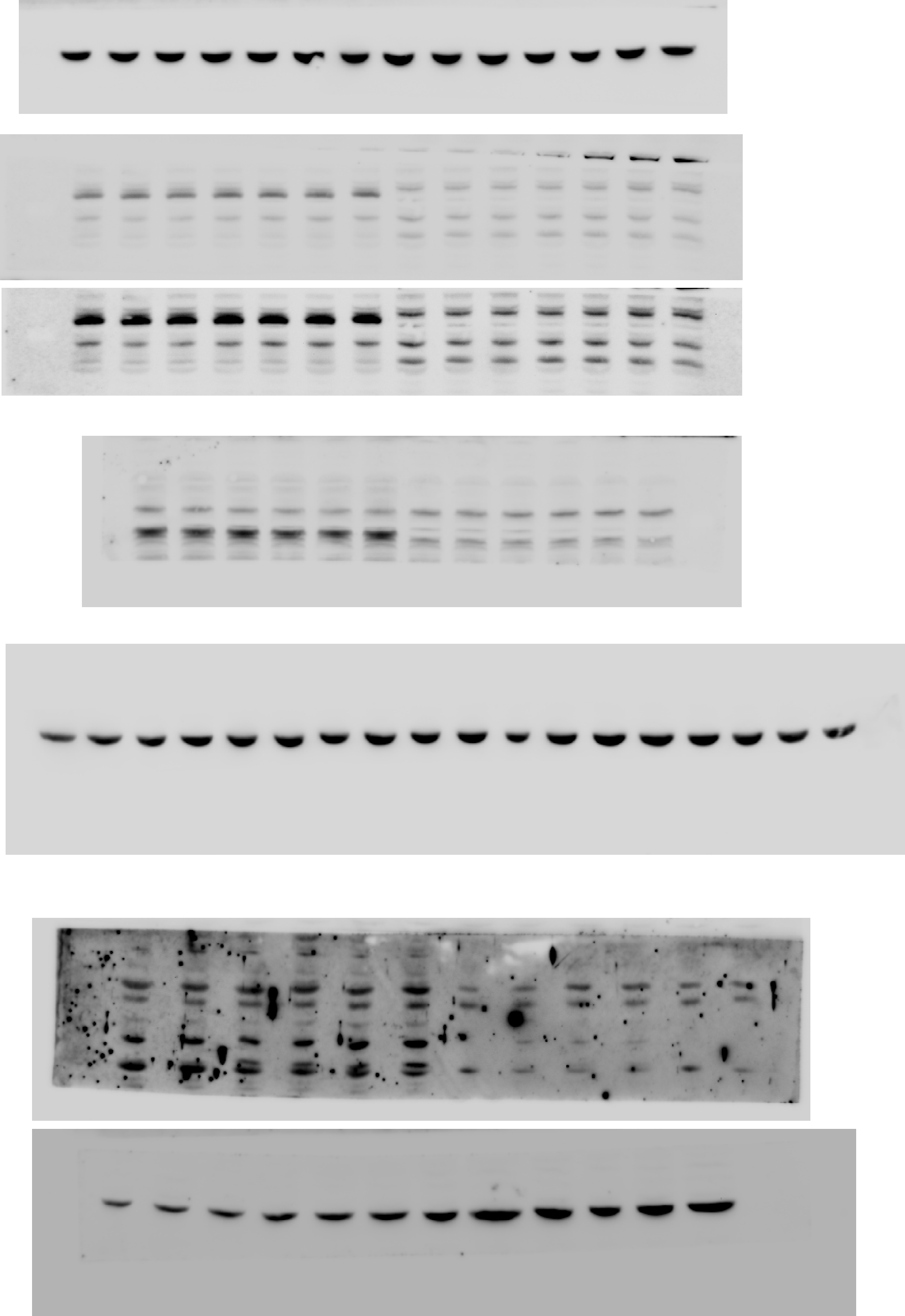

Supplement: Figure 8—figure supplement 1—source data 2. [file elife-82115-fig8-figsupp1-data2.tif]
