# Supplementary material for: Isoform-specific disruption of the TP73 gene reveals a critical role for TAp73γ in tumorigenesis via leptin
Source: eLife. 2023 Aug 31;12:e82115. doi: 10.7554/eLife.82115 (PMC10471163; doi:10.7554/eLife.82115)
Supplement: Supplementary file 1. — (a) Wild-type (WT) mice (n = 56) – survival time, tumor spectrum, steatosis, inflammation, and other abnormalities. (b) Trp73+/- mice (n = 30) – survival time, tumor spectrum, steatosis, inflammation, and other abnormalities. (c) E11-HET mice (n = 30) – survival time, tumor spectrum, steatosis, inflammation, and other abnormalities. [file elife-82115-supp1.docx]

Supplementary file 1a: Wild-type (WT) mice (n=56) - survival time, tumor spectrum, steatosis, inflammation, and other abnormalities


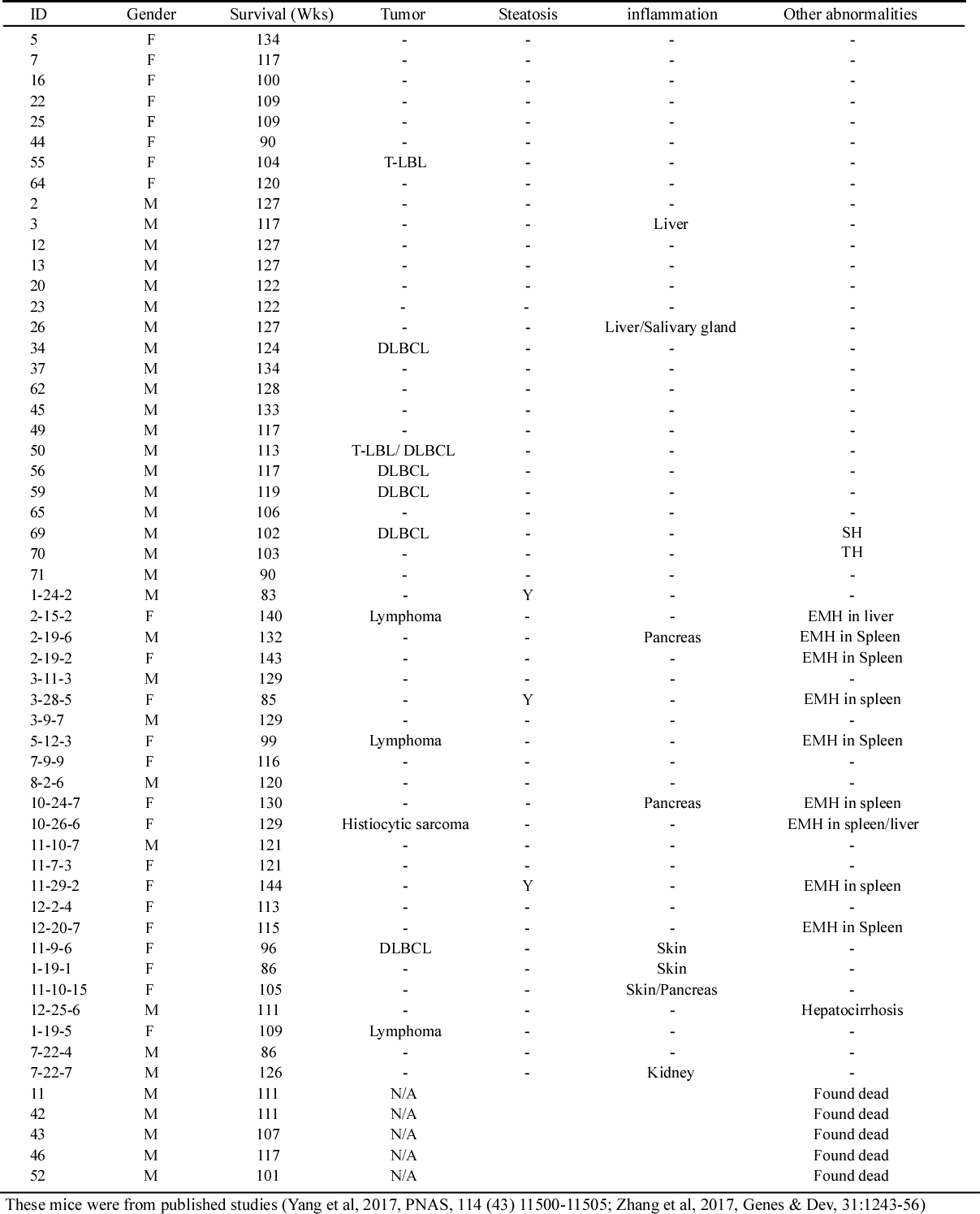


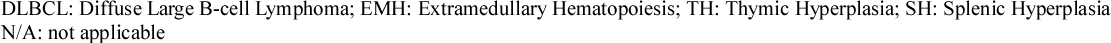


Supplementary file 1b: *Trp73^+/-^* mice (n=30) - survival time, tumor spectrum, steatosis, inflammation, and other abnormalities


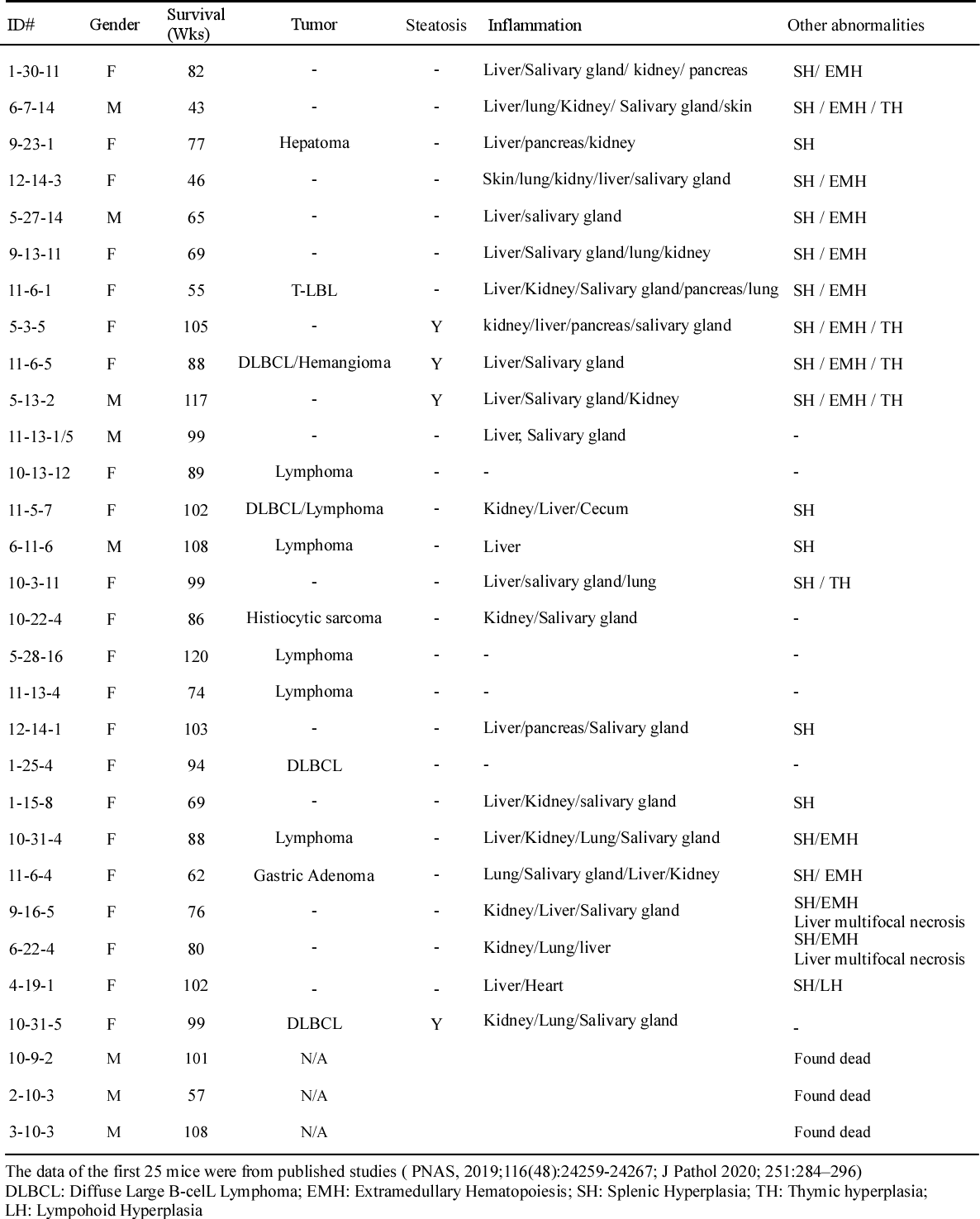


Supplemental File 1C: E11-HET mice (n=30) - Survival time, tumor spectrum, steatosis, inflammation, and other abnormalities


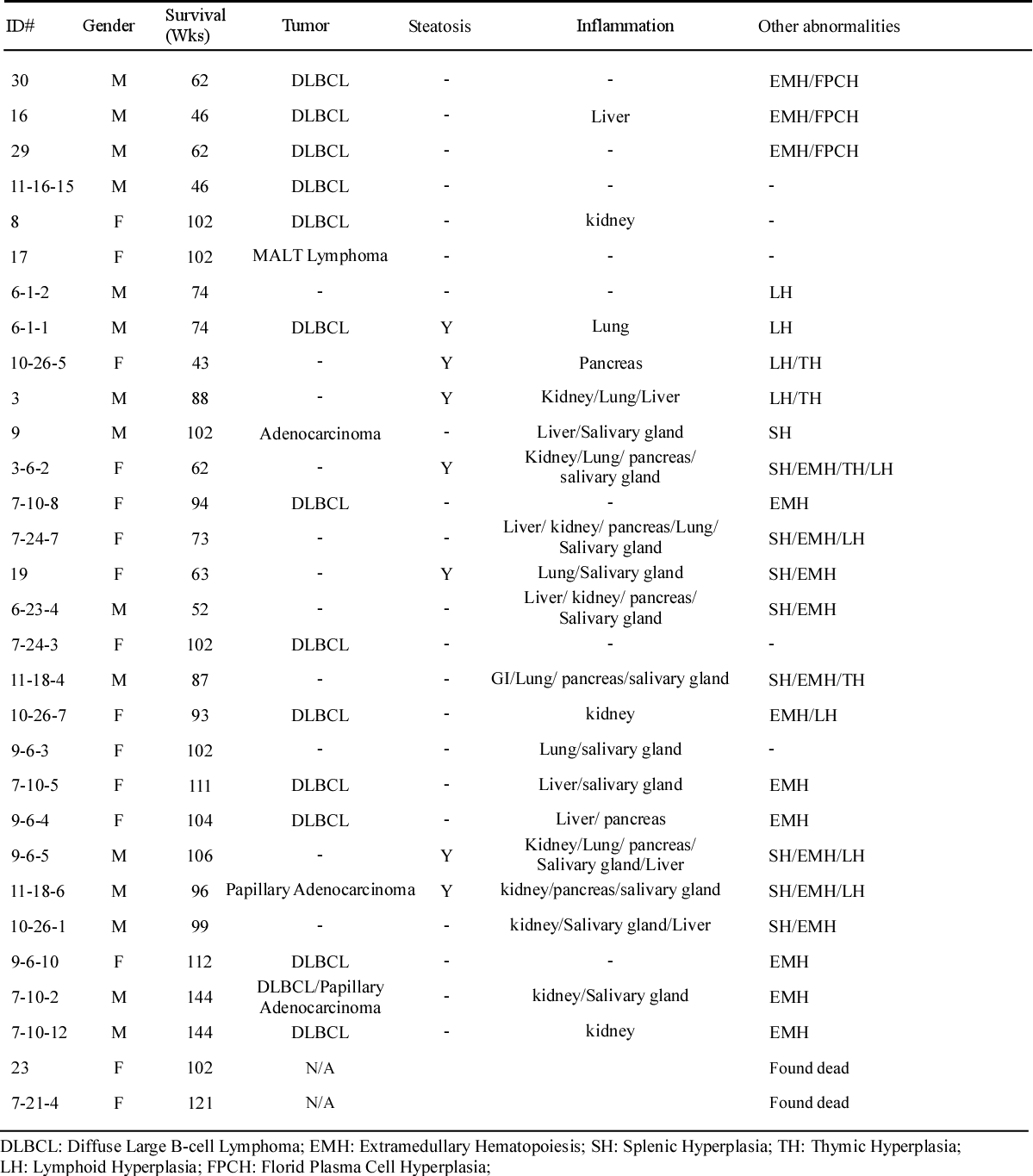


**Supplemental File 2a**. The primers used to generate plasmids expression vectors

| Name | Oligonucleotides | Sequence |
| --- | --- | --- |
| TAp73-sgRNA-1 | Sense | 5’-ACCGCTTCCCCACGCCGGCCTCCG-3’ |
|  | Antisense | 5`-AAACCGGAGGCCGGCGTGGGGAAGC-3` |
| TAp73-sgRNA-2 | Sense | 5`-CACCGTCAAACGTGGTGCCCCCATC-3` |
|  | Antisense | 5`-AAACGATGGGGGCACCACGTTTGAC-3` |
| ΔNp73-sgRNA-1 | Sense | 5`-CACCGTACAGCATGGTAGGCGCCG-3` |
|  | Antisense | 5`-AAACCGGCGCCTACCATGCTGTAC-3` |
| ΔNp73-sgRNA-2 | Sense | 5`-CACCGCGTCACACCTACCGTGGCG-3` |
|  | Antisense | 5`-AAACCGCCACGGTAGGTGTGACGC-3` |
| Total p73-sgRNA | Sense | 5`-CACCGCACCTTCGACACCATGTCGC-3` |
|  | Antisense | 5`-AAACGCGACATGGTGTCGAAGGTGC-3` |
| p73-E11-gRNA-1 | Sense | 5`-CACCGTCAGAAGCAACGGCTCGCAT-3` |
|  | Antisense | 5`-AAACATGCGAGCCGTTGCTTCTGAC-3` |
| p73-E11-gRNA-2 | Sense | 5`-CACCGACAGCCCCCGTCCTACGGGC-3` |
|  | Antisense | 5`-AAACGCCCGTAGGACGGGGGCTGTC-3` |
| WT Leptin promoter | Forward | 5'- CCC CCC GGG TCG CTG GAC CTT AGA TTC CTC ATC C -3' |
|  | Reverse | 5'- CCC AAG CTT CTT GCA ACC GCT GGC GCT G3’ |
| Mut Leptin promoter | Forward | 5'- CCC CCC GGG TCG CTG GAC CTT AGA TTC CTC ATC C -3' |
|  | Reverse | 5'- CTT CCA CCT GGG CTG GTC AGG TCG CCA -3' |
| TAp73γ | Forward | 5'- GAA TTC GTG GGG AGG GAA TTC ACC -3' |
|  | Reverse | 5'- CTC GAG TCA ATG GTC AGG TTC TGC AG -3' |

**Supplemental File 2b**. The primers used for genotyping

|  | Oligonucleotides | Sequence |
| --- | --- | --- |
| *P73-*KO  mouse genotyping | Sense 1 (*p73*KO-F) | 5′‐ CATGTCTGGATCCGGAATAACTAACT ‐3′ |
|  | Sense 2 (WT-F) | 5′‐ CACGAGCTTGGAAGGGACTT ‐3′ |
|  | Antisense (common-R) | 5`- TC TTTAGGTGGCCTTTGAGG -3` |
| *E11-*KO  mouse genotyping | Sense | 5`- CCGAGATCACAAACAGGATG -3` |
|  | Antisense | 5`- CTACAGCTGCCTCTCAATG -3` |
| *E11-*KO  cell line genotyping | Sense | 5`- TCCACCCATTCGCAGCATG -3` |
|  | Antisense | 5`- CACTGCCCAAGGGACTCAC -3` |
| *ΔNp73-*KO  cell line genotyping | Sense | 5`- ACTAGCTGCGGAGCCTCTC -3` |
|  | Antisense | 5`- GCGTCACACCTACCGTGG -3` |
| *TAp73-*KO  cell line genotyping | Sense | 5`- GAGGCTGTCACAGGAGGAC -3` |
|  | Antisense | 5`- TCTGGCCAGCCAAGCGCAC -3` |

**Supplemental File 2c**. The primers used for RT-PCR and CHIP

| Name | Oligonucleotides | Sequence | Fragment |
| --- | --- | --- | --- |
| *TP73*(human) | Sense | 5’- CAGACAGCACCTACTTCGAC -3’ |  |
|  | Antisense | 5`- CTGCTCATCTGGTCCATGG -3` | 255bp |
| LEP (human) | Sense | 5`- TCACACACGCAGTCAGTCTC -3` |  |
|  | Antisense | 5`- GAGGTTCTCCAGGTCGTTGG -3` | 177bp |
| p73α/ γ (human) | Sense | 5`- CAGCAGCAGCAGCTCCTACA -3` |  |
|  | Antisense | 5`- GAAATACTCGATGCAGTTTG -3` | 207/356bp |
| *TP73-*All (human) | Sense | 5`- CAGCAGCAGCAGCTCCTACA -3` |  |
|  | Antisense | 5`- TACTGCTCGGGGATCTTCAG -3` | 94~440bp |
| *ΔNp73* (human) | Sense | 5`- GATCCATGCCTCGTCCCAC -3` |  |
|  | Antisense | 5`- CTGCTCATCTGGTCCATGG -3` | 155bp |
| GAPDH (human) | Sense | 5`- AGCCTCAAGATCATCAGCAATG -3` |  |
|  | Antisense | 5`- ATGGACTGTGTCATGAGTCCTT -3` | 198bp |
| *TP73* (mouse) | Sense | 5`- AAGGGACTAGCGAGGCATCA -3` |  |
|  | Antisense | 5`- CCGGGGTAGTCGGTATTGGA -3` | 232bp |
| Il6 (mouse) | Sense | 5`- GAGGATACCACTCCCAACAGACC -3` |  |
|  | Antisense | 5`- AAGTGCATCATCGTTGTTCATACA -3` | 284bp |
| Lep (mouse) | Sense | 5`- GGTCACTGGCTTGGACTTCA -3` |  |
|  | Antisense | 5`- CAGATGGAGGAGGTCTCGGA -3` | 163bp |
| TNFα (mouse) | Sense | 5`- TGGCCTCCCTCTCATCAGTT -3` |  |
|  | Antisense | 5`- ACAAGGTACAACCCATCGGC -3` | 165bp |
| IL1a (mouse) | Sense | 5`- CTCTAGAGCTCCATGCTACAGAC -3` |  |
|  | Antisense | 5`- TGGAATCCAGGGGAAACACTG -3` | 267bp |
| p73α/ γ(mouse) | Sense | 5`- GTGCCCCAGCCTTTGGTTGAC -3` |  |
|  | Antisense | 5`- GTGAACTCCTCTTTGATGGG -3` | 198/346bp |
| Actin (All) | Sense | 5`- CTGAAGTACCCCATCGAGCACGGCA -3` |  |
|  | Antisense | 5`- GGATAGCACAGCCTGGATAGCAACG -3` | 221bp |
| p73α/ γ (dog) | Sense | 5`- GGGGCATCATGTGGAAATG -3` |  |
|  | Antisense | 5`- TCTCGCTCTCCGTGAACTC -3` | 192/361bp |
| LEP (dog) | Sense | 5`- CTCCTCCAAACAGAGGGTCG -3` |  |
|  | Antisense | 5`- CCCAGGCTCTCAAAGGTCTC -3` | 243bp |
| GAPDH-chip | Sense | 5`- AAAAGCGGGGAGAAAGTAGG -3` |  |
|  | Antisense | 5`- AAGAAGATGCGGCTGACTGT -3` | 202bp |
| Leptin-chip | Sense | 5`- AGGGCCGTCGGGGCCGAGTCC -3` |  |
|  | Antisense | 5`- AAATCCTTGATGTCCCTCC -3` | 210bp |
| P21-chip | Sense | 5`- GGAAGACCATGTGGACCTGT -3` |  |
|  | Antisense | 5`- GGCGTTTGGAGTGGTAGAAA -3` | 225bp |
| Alt (mouse) | Sense | 5`- CGCGTCTTTCCACTCAGTCT -3` |  |
|  | Antisense | 5`- ACCTGCTCCGTGAGTTTAGC -3` | 270bp |
| Ast (mouse) | Sense | 5`- CAGGGAGAATCGGGTTGGAG -3` |  |
|  | Antisense | 5`- CTTCTCCGCATCCCAGTAGC -3` | 211bp |
| Ggt1 (mouse) | Sense | 5`- TCTGTTGCACTGGCCATCAT -3` |  |
|  | Antisense | 5`- CTCGAACGACAGCCTGAACT -3` | 211bp |
| LepR (human) | Sense | 5`- CTGTGCCAACAGCCAAACTC -3` |  |
|  | Antisense | 5`- ATGGTACCAATGGTGGGCTG -3` | 198bp |
